# Repeats Influence Structural DNA Properties Around Functional Annotations Associated with 3D Organization and Transcription

**DOI:** 10.3390/genes16091082

**Published:** 2025-09-15

**Authors:** Aaron Sievers, Michael Hausmann, Georg Hildenbrand

**Affiliations:** 1 Kirchhoff-Institute for Physics, Heidelberg University, INF 227, 69117 Heidelberg, Germany; aaron.sievers@med.uni-heidelberg.de (A.S.); georg.hildenbrand@th-ab.de (G.H.); 2 Institute for Human Genetics, University Hospital Heidelberg, INF 366, 69117 Heidelberg, Germany; 3 Faculty of Engineering, University of Applied Science Aschaffenburg, Würzburger Str. 45, 63743 Aschaffenburg, Germany

**Keywords:** DNA repeats, tandem repeats, DNA properties, structural DNA properties, DNA dinucleotides, transposons, transcription regulation, chromatin organization

## Abstract

Background: While the fundamental principles of chromatin 3D organization and its interplay with transcriptional regulation are still not completely understood, increasing evidence suggests a considerable role of DNA repeats. Considering the influence of DNA repeats on local dinucleotide contents, the influence of dinucleotide contents on the structural properties of DNA, their influence on histone affinity, and the influence of histone occupancy on chromatin, an indirect influence of repeats on 3D organization, seems worth testing. Methods: In this study, we search for global correlations between annotations associated with transcription and 3D organization, dinucleotide contents, DNA properties, and repeats in human and mouse. In a second step, we search for local peaks in DNA properties around those annotations and derive the influences of the dinucleotide and repeat contents (including tandem repeats (TRs) and transposons). Results: We identify several strong and significant associations between annotations and DNA properties, which are influenced by a variety of different repeats. Consistent with former findings, the Roll property is found to be especially sensitive to the sequence context. Conclusions: Our results suggest a significant effect of repeats on DNA properties and thus an indirect effect on histone occupancy and 3D chromatin organization.

## 1. Introduction

Despite decades of research [1], the major functional relationships between the DNA sequence and transcription regulation remain incompletely understood [2,3,4]. While the presence of sequence features, like genes, promoters, and enhancers, provides a solid framework, the existence of these annotations cannot inherently explain the spatiotemporal regulation of transcriptional activity in living cells [5]. One of the most relevant intermediate layers connecting sequence and function is the 3D chromatin organization [2,6,7]. The organization of chromatin into larger structures, especially into topologically associated domains (TADs), is believed to strongly restrict interactions between regulatory proteins and genes and thus to strongly influence their transcriptional activity [8]. While some recent studies suggest a passive formation of TADs, driven by histone positioning [4], it is widely believed that architectural proteins such as CTCF and cohesin are key factors for TAD formation [2]. In a formation process called loop extrusion, the binding of CTCF to DNA at specific sequence motifs is crucial for determining TAD boundaries and thus for defining major parts of the chromatin 3D organization [9].

The formation and stability of 3D chromatin structures is also dependent on the dynamic properties of the chromatin polymer [10], which mainly consists of DNA wrapped around core histones, connected by stretches of linker segments. Those properties of chromatin are determined by the positioning of histones and the properties of linker segments (e.g., its length and stiffness) [4,10], both of which are influenced or determined by the dynamic and structural properties of the associated DNA molecules (e.g., its Twist, Roll, or persistence length). Linker segments directly consisting of DNA (often A/T-rich, very stiff stretches [11]) and the histone positioning are influenced by the effects that the intrinsic curvature of wrapped DNA and, especially, the Roll property of DNA have on the local binding affinity for histones [12].

Several studies found additional interesting relationships between structural properties of DNA and secondary properties (related to functions) or directly between structural properties and biological function. Many structural properties of DNA (Roll, Tilt, Twist, Shift, Slide, Rise, Propeller, and many groove-related properties) are associated with binding of transcription factors (TFs) [13,14,15]. The Roll, Tilt, Twist, Shift, Slide, and Rise were also found to influence histone affinity [12,16,17], with some sources reporting significant influences of Rise [17]. Some found considerable influences of Twist and Slide [12], and strong influences of the Roll property have been very consistently reported, with studies having found that the intrinsic Roll of DNA alone is predictive for 92% of histone binding [12,16]. Structural properties also influence DNA flexibility [18,19,20,21], supercoiling [22,23], and, in some cases, mutation rates or damage [24,25]. Again, the Roll property is often found to have the most prominent effects. Since those structural DNA properties are known to be mainly determined by the local dinucleotide contents [26], influences of DNA properties on transcription, histone occupancy, and, thus, 3D chromatin organization might be indirect influences of underlying dinucleotide contents.

Those local dinucleotide contents are sensitive to the presence of other sequence patterns. One very abundant class of such patterns is DNA repeats, which cover more than 50% of the human genome [27]. One main class of repeats in eukaryotic genomes is interspersed repeats, which are mainly transposons of the Alu (a type of SINE) or B1 (Alu-equivalent in MM) and LINE1 (L1) families in humans and mice [28]. Higher densities of G+C- and AG-rich Alu elements [29,30] were found in gene-rich regions [31]. In some cases, they provide binding sites for functional elements or provide alternative promoters or enhancers to genes [32,33]. G+C-rich Alu elements also influence transcription by DNA methylation [30] and affect histone occupancy [34]. L1 elements have higher contents of A+T and are preferentially found in heterochromatic, gene-poor regions [35]. Accordingly, there is less evidence for influences of L1 on transcription. It has, however, been proposed that L1 includes CTCF binding sites [36]. Recently, more and more studies propose major roles of Alu and L1 in 3D organization [37,38,39]. Another major class of repeats in eukaryotes is so-called tandem repeats (TRs), which are (often multiple) head-to-tail repetitions of identical sequences (repeat units) Typical repeat units are short (1–6 bp), therefore sometimes also being referred to as “simple” (e.g., simple sequence repeats), and are repeated several times (e.g., AGAGAG = (AG)_3_) [40]. Their relatively high mutation rates lead to significant-length polymorphisms across individuals and species, providing a potential source of very fast variation and evolution [41]. TRs are often associated with promoters, enhancers, and genes [41]. Variations in the TR length influence the spacing between transcription factor binding sites and promoters or influence nucleosome positioning [40]. (A)_n_ and (T)_n_, especially, were known to disfavor nucleosome formation [42]. TFs were also found to directly bind and, therefore, be guided by TRs through the providing of a high number of binding sites with relatively low binding affinity [40,43]. The hypothesis of TRs having functional roles is further supported by several genome-wide studies that linked TRs with nearby gene expression [44]. The evidence for effects on 3D organization is less extensive but does exist [40,45].

Since a considerable amount of eukaryotic genomes consist of DNA repeats, it seems plausible that local dinucleotide contents are strongly affected by their presence. This influence of repeats on dinucleotide contents might lead to influences on structural properties, which could then lead to influences on large-scale 3D chromatin structures.

In summary, it seems quite plausible that chromatin 3D organization and its transcription are influenced by more fuzzy sequence features like DNA repeats and dinucleotide contents, indirectly, through their respective influence on DNA properties. Importantly, it is unclear to what extent these properties contribute to the formation or stability of 3D structures and, more generally, to the regulation of transcription.

In this study, we aim to search for indicators of these indirect influences in the positional relationship between associated annotations (e.g., genes, enhancers, CTCF), sequence features (e.g., dinucleotide contents and repeat contents), and DNA properties within the genomic sequences of *Homo sapiens* (HS) and *Mus musculus* (MM). First, we use dinucleotide-based models to predict local values for DNA properties around functional annotations and identify significant local variations. We then examine the influences that local dinucleotide and repeat contents have on these local patterns of structural DNA properties. Finally, we highlight interesting cases and discuss potential implications for the role of dinucleotides and repeats in transcription regulation and 3D chromatin organization.

## 2. Materials and Methods

### 2.1. Genomic Maps

Using the Oligo software package (version 5.0) [46], we derived local (resolution of 1000 bp) dinucleotide and repeat content based on chromosomal DNA sequences of HS and MM extracted from GenBank files and downloaded from the NCBI website [47]. Local contents of transposons (Alu, L1) in 1000 bp resolution were derived from data files downloaded from the RepeatMasker website [48]. Local contents of genes were derived based on gene annotations from GenBank files for the respective chromosomes [47]. Local contents of promoter, enhancers, transcription factor binding sites, and CTCF binding sites were derived from data files (GTF format) downloaded from the Ensembl FTP website [49]. According to the description at Ensemble (https://www.ensembl.org/info/genome/funcgen/index.html—accessed on 28 August 2025) [49], the source data contain a union set of annotations derived from multiple cell-lines. Positions of TAD borders for embryonic stem cells, first published in [50], were downloaded in BED format from the 3D genome browser website (https://3dgenome.fsm.northwestern.edu/downloads/—accessed on 3 February 2023) [51]. Results are genomic maps for HS and MM, with consistent resolution of 1000 bp for each dinucleotide, repeat, and functional annotation listed.

### 2.2. Structural DNA Properties

We downloaded the complete database (126 different dinucleotide models) from the Dinucleotide Properties Genome Browser [52]. Each model consists of 16 values (one for each dinucleotide). A model is applied to a given sequence by summarizing the products of dinucleotide content and respective model values. Accordingly, DNA property maps are directly modelled based on the genomic maps of dinucleotides. The influence of repeats can be indirectly derived by first deriving their influence on local dinucleotide contents.

### 2.3. Reference Models for Dinucleotide Contents

We used expectation values from zero-order Markov models, based on empirical local nucleotide content, to derive reference values representing a null hypothesis of dinucleotide content resulting from random shuffling of local nucleotides (Equation (1)).

(1) $$E[n_{XY}]=\frac{n_{X}n_{Y}}{L_{seq}}$$

Here, E[n_XY_] is the expectation value of the local dinucleotide content n_XY_ for dinucleotide XY (X, Y ∈ {A, C, G, T}), n_X_ is the empirical content of nucleotide X, and L_seq_ is the length of the modelled sequence (1000 bp in this study). For a second reference, representing a null hypothesis of a uniform distribution of dinucleotides, we derived genomic mean values and uncertainties based on empirical dinucleotide content.

### 2.4. Reference Models for Repeat Content

We used expectation values from first-order Markov models, based on empirical local dinucleotide content, to derive reference values representing a null hypothesis of TR content resulting from local dinucleotide content (Equation (2)). We limited our definition of TRs to a minimum length of 4 bp (at least 2 repeated units, n ≥ 2) and did not differentiate between TRs with repeat units consisting of the same nucleotides, e.g., ACAC and CACA are both considered as repeats of (CA)_n_.

(2) $$E[n_{{\left(XY\right)}_{n\geq 2}}]=\frac{n_{XY}^{2}n_{YX}}{{n_{X}n}_{Y}}$$

### 2.5. Local Environments

We used Oligo [46] to derive local density environments (e.g., of dinucleotide contents) around functional annotations (e.g., genes or TAD borders), based on the genomic maps with 1000 bp resolution described above. The underlying algorithm starts at a given anchor position (e.g., a gene) and gradually increases the distance from that position while collecting the density from a genomic map (e.g., dinucleotide content). This procedure is repeated for every annotation within a dataset (e.g., for all human genes). Afterwards, a mean value and variance is derived for each distance. For the derivation, the interiors of annotations are masked such that only the true environment (which is not part of an annotation) is considered for the calculations. This removes potential biases from a clustering of annotations (e.g., if promoters are preferentially located near genes). If more than 50% of a 1000 bp segment was masked this way, the segment was discarded from the calculation, to exclude biases from overly short sequences. The remaining segments were weighted based on their unmasked sizes to correct for the expected lower numbers of annotations (e.g., dinucleotides or repeats).

### 2.6. Peak Calling

In the simplest case, if there is a relation between the environmental densities and the anchor annotations, we expect a central peak or dip within the density environments described above. Therefore, we use an algorithm for explicitly identifying and evaluating those central peaks/dips. The algorithm starts by deriving a baseline value for the environment. This is done by deriving a mean value and standard deviation for values at sufficiently high distances (>800,000 bp in this study), assuming that annotations will not have any influence on the environment at such distances. The algorithm then starts at the center (distance of 0). The algorithm checks if the local environmental density is significantly higher or lower than the baseline value (considering uncertainties of both values). If such a peak was detected, it gradually increases the distance in both directions, until the difference to the baseline is no longer significant. The maximum difference to the baseline value that is observed is considered the height of the respective peak, while the maximum and minimum distances with significant differences from the baseline define the width of the peak.

### 2.7. Correlation

To reduce computational costs and introduce an error estimation for the derivation of correlation values between large genomic maps, we used a bootstrapping algorithm (50 repetitions with 50 randomly selected positions). For each selected position, values from both correlated maps were extracted. The result is a 50-dimensional vector for each map, for a standard Pearson coefficient [53] was then derived. This process was repeated 50 times to derive a mean value and standard deviation for resulting correlations. To derive a reference value, the same procedure was repeated, using a random shuffled version of the maps. The significance level σ of the resulting correlation was then defined as the difference between empirical and shuffled mean values, divided by its uncertainty. A similar approach, described and applied in [46], was tested. To reduce associated bias, centromere regions and sequencing gaps were masked for the correlations.

## 3. Results

### 3.1. Correlations Between Structural Properties & Functional Annotations

**Figure 1:**
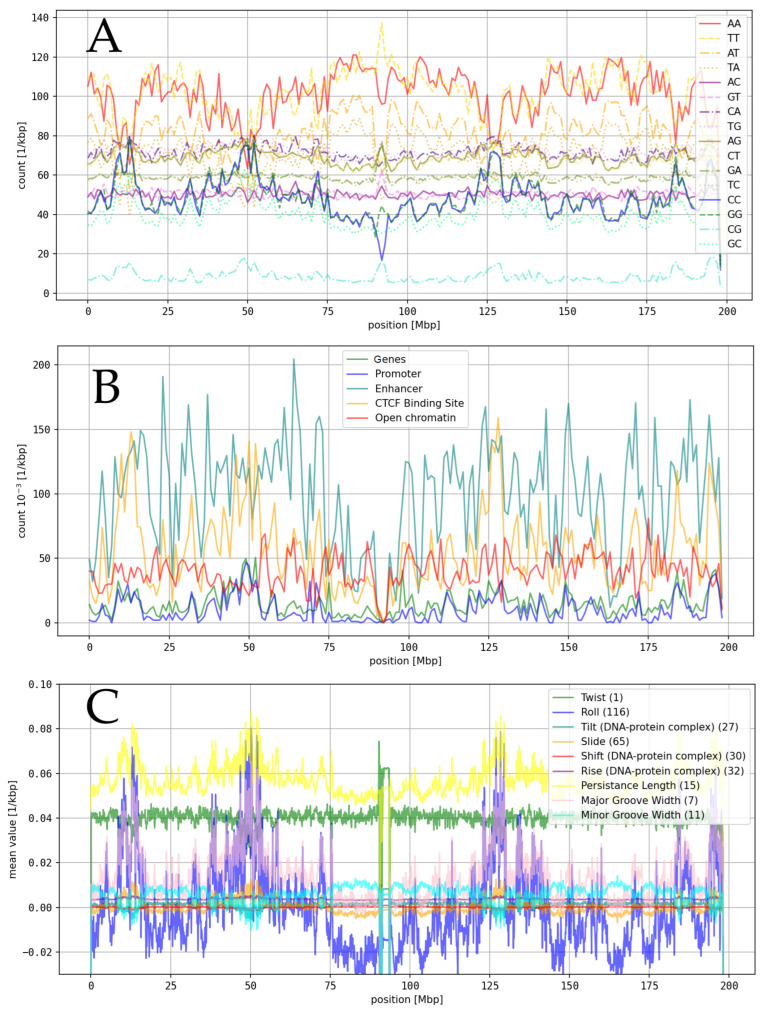
Genomic maps: shown are genomic maps of HS c3 with a resolution of 1 Mbp. (**A**): Maps of functional annotations, derived from GenBank files [47] and datafiles downloaded from Ensembl. (**B**): Dinucleotide maps, derived by counting the number of dinucleotide occurrences within the sequences of 1 Mbp segments extracted from GenBank files [47]. (**C**): Selection of DNA property maps, derived based on dinucleotide maps, using models from the Dinucleotide Properties Genome Browser [52]. To improve visibility, variances in (**C**) were scaled by a factor of 100 (except for persistence length).

We derived genomic maps for functional annotations and dinucleotides. Using models extracted from Dinucleotide Properties Genome Browser (version updated on 14.06.2010) [52], we derived genomic maps for a large variety of structural and physical properties of DNA, based on the obtained dinucleotide maps (see Figure 1). To search for global genomic relationships, we pairwise correlated maps of functional annotations and DNA properties (see Table 1 and Table S1). Correlations were repeated for map resolutions of 10 kbp and 1 Mbp to confirm the results and robustness of the analysis (see Tables S5 and S6).

Interestingly, significant correlations were observed for most pairs of DNA properties and functional annotations. Considering the observed correlations, the high level of consistency between HS and MM is remarkable. Not only is the direction/sign of respective correlations mostly conserved between genomes of HS and MM, but so is the level of correlation. The correlations are also mostly consistent between the different functional annotations. A positive correlation between one DNA property and one functional annotation nearly always implies a positive correlation with all other functional annotations and vice versa. To some degree, this result is expected, since there are well-known positional correlations between different functional annotations based on their functional interplays (e.g., promoters and other regulatory elements are often in close proximity to their associated genes). While correlation values between maps of functional annotations in our dataset (see Table S2) confirm this expectation, their level is significantly lower compared to the correlation values with DNA properties (see Table 1). Also interesting is the observation that the correlation results mostly agree between alternative models for the same property, especially for the Rise, Roll, and Twist properties. In the context of chromatin organization, it is also remarkable that the highest correlations were observed between maps of DNA properties and CTCF binding sites and that, for flexibility-related DNA properties (e.g., persistence length), relatively high correlation values were observed.

### 3.2. Peaks in Structural Properties Around Functional Annotations

**Figure 2:**
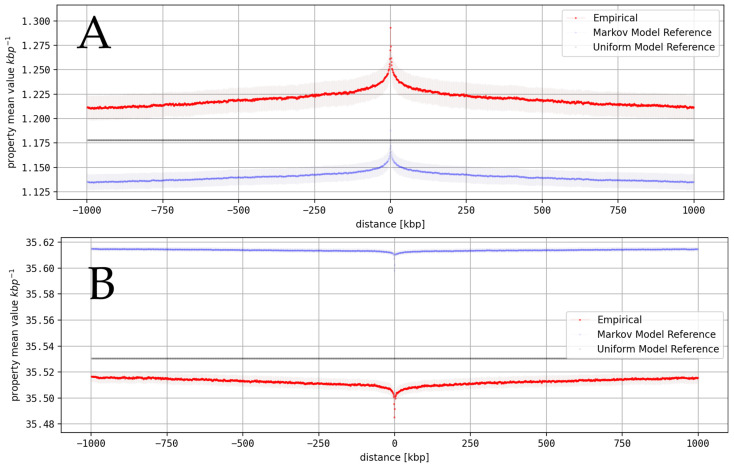
Structural property profiles around functional annotations: Illustrative examples for local mean values of structural DNA properties around functional elements, derived based on genomic maps with 1000 bp resolution. Shown are empirical profiles (red) and reference data derived based on Markov chain models (blue) and uniform distributions (black). (**A**) positive peak of Roll (94) around CTCF in HS. (**B**) negative peak of Twist (92) around genes in HS.

We derived local environments of all structural DNA properties within our dataset, and these were centered around genes, promoters, enhancers, TF binding sites, CTCF binding sites, and TAD borders. In most cases, central peaks or dips were visible, which indicates a positional relationship between the environment and functional element (see Figure 2).

**Figure 3:**
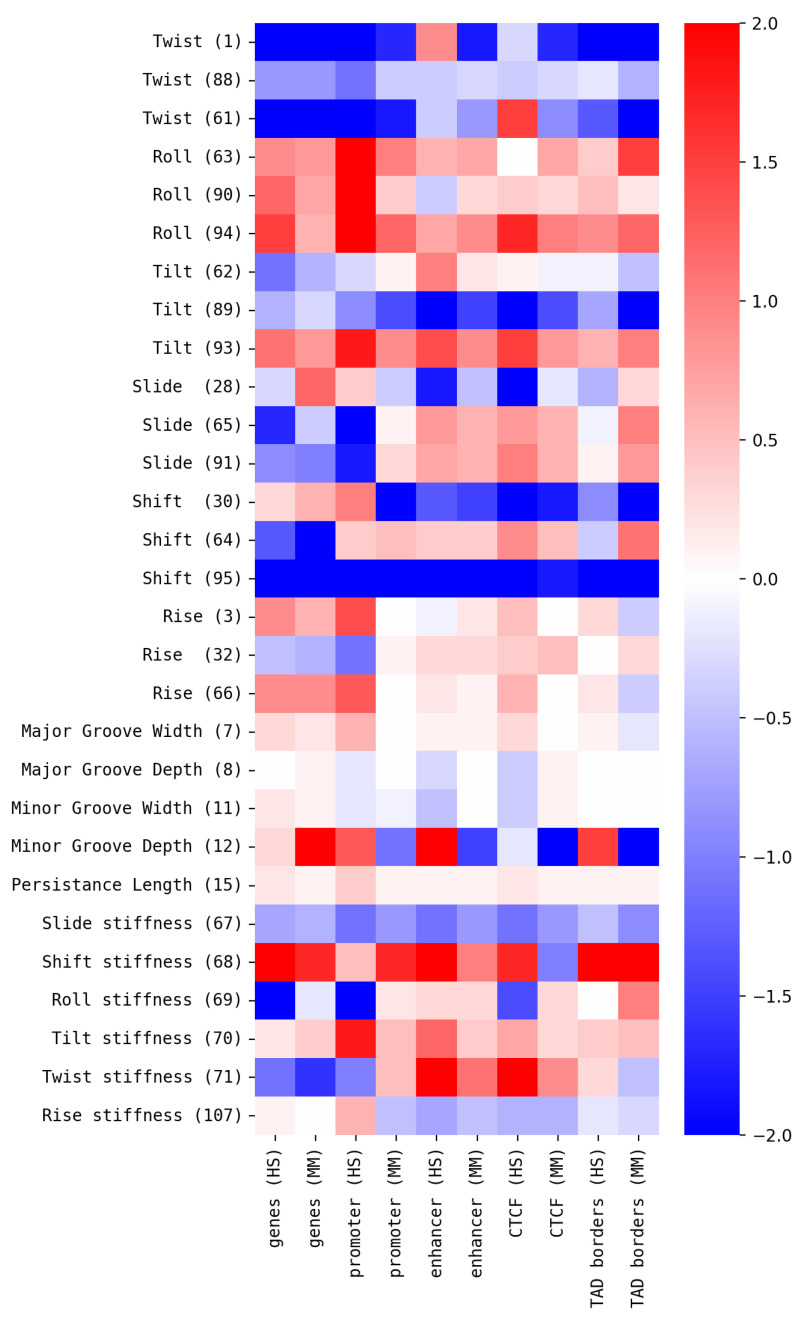
Peaks in property profiles around functional annotations: Peaks in empirical and reference profiles of local values of structural DNA properties were identified. Shown are significance values (σ) of peak heights relative to reference data. See Table S3 for details on peak height and peak width and data for all property models.

We identified significant central peaks using the algorithm described above (see Table S3). In the reference data, especially the Markov models based on local dinucleotide environments, similar peaks were frequently visible (see Figure 3, Table S3). Therefore, we evaluated the significance of an identified peak not only in relation to its baseline but also considering differences in its peak height in relation to peaks within the reference data. This procedure removes potential influences of local deviations in nucleotide or G+C content that could lead to differences in dinucleotide contents and thus peaks in structural properties.

While, for models of Tilt, Slide, Shift, and Rise, inconsistent results were often observed (negative or positive peaks, depending on the model), the peaks for Twist and Roll were consistent for most models, showing a positive peak for Roll around functional annotations and a negative peak (or dip) for Twist. The peaks around DNA groove-related properties are mostly insignificant or inconsistent between HS and MM. For the persistence length, we also only observe insignificant peaks around functional annotations. Interestingly, we observe peculiar patterns for other flexibility-related properties, including the Twist stiffness and also the Slide stiffness and Shift stiffness. The twist stiffness is low around genes and high around enhancers and CTCF. The results for the Roll stiffness were mostly inconsistent for HS and MM.

### 3.3. Influences of Dinucleotides & Repeats

**Figure 4:**
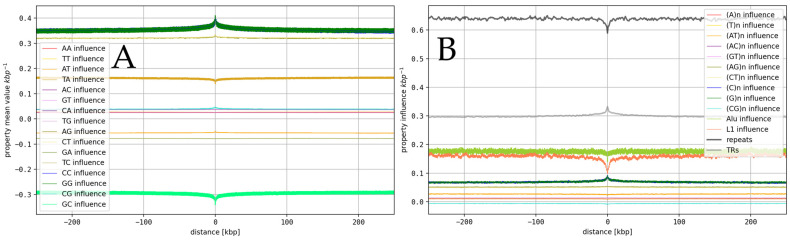
Influence of dinucleotides and repeats on Roll around CTCF: Illustrative example of the influence of dinucleotides and repeats on the Roll properties. Shown are results for Roll (94) in HS. (**A**): Influences of dinucleotides on Roll. (**B**): Influences of repeats (TRs, Alu and L1) on Roll.

Based on the DNA property models, we derived influences of individual dinucleotides and repeat contents on DNA properties around functional annotations and corresponding peaks (see Figure 4, Table S4).

**Figure 5:**
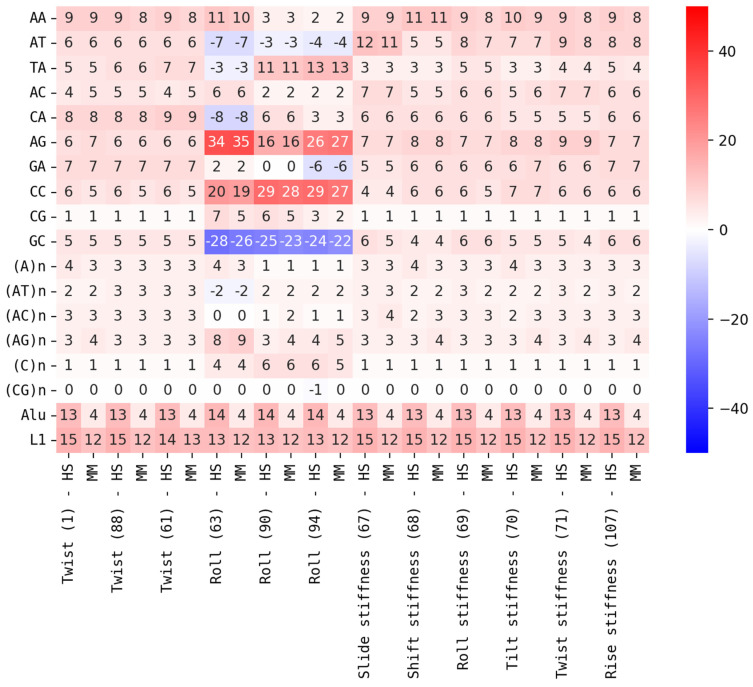
Relative influence of dinucleotides/repeats on DNA properties around CTCF: relative influences (in percent [%]) of a representative selection of dinucleotides/repeats on peaks of structural DNA properties around CTCF binding sites (see Figure S1 for other functional annotations, Table S4 for other dinucleotides/repeats).

In general, we observe that the influences of dinucleotides on DNA properties around functional annotations are complex. Properties are influenced by dinucleotides in different directions, often by dinucleotides with identical G+C content. In the case of Roll (Figure 4), GC dinucleotides have the highest negative influence, while CC and GG have the highest positive influence around CTCF binding sites. The relative level of dinucleotide influences is also often in the range of 10–40% (see Table S4, Figure 5) for several individual dinucleotides, which implies that the resulting property is a tightly balanced average between strong influencing factors. Influences of repeats are generally smaller but still very considerable, especially when combined (see Figure 4 and Figure 5). The influences for dinucleotides and repeats are almost always consistent between HS and MM and between different functional annotations (see Figure 5 and Figure S1). The differences between HS and MM are less than 1–2% in most cases. An exception is the influence of Alu. For transposons of the Alu family, we observe influences of around 13–14% on the peaks of all structural properties around each annotation for HS, while only observing values in the range of 4% for MM. This might indicate a special role of Alu in HS when compared to its role in MM. AA/TT (~9%) dinucleotides and (A)n/(T)n TRs (~3%) show tendencies to have the highest average influence within their respective categories. Nevertheless, we observe higher values for other dinucleotides/repeats for peaks of specific DNA properties. Most prominent here is the Roll property (see Figure 5).

Interestingly, only a few models for Roll indicate a high influence of AA/TT, while we observe a consistently high influence of AT and TA (in different directions), and extremely high influences of AG/CT (~30%), CC/GG (+30%), and GC (−25%). Influences of (AG)_n_ (4–9%) and (C)_n_/(G)_n_ (~5%) on Roll are also very high compared to those of other TRs. In contrast, the influence of Alu (which is known to be G+C and rich in AG dinucleotides) is only slightly elevated (+1%) in HS and unaffected in MM, when compared to its influences on peaks of other properties. It might also be interesting that the influences of CG, (CG)_n_, and (C)_n_/(G)_n_ are very weak for most properties. This makes the relatively high influence of (C)n on Roll (up to 6%) even more remarkable. Also, CG reaches influences up to 7%, but only for the Roll property.

## 4. Discussion

The main focus of this study was the search for evidence that local dinucleotide and DNA repeat contents indirectly influence chromatin 3D organization and/or transcription by influencing dynamic and/or structural DNA properties. First, we found high and significant correlations between DNA properties, which were modelled based on the local dinucleotide content, and functional annotations associated with transcription and 3D organization, such as genes, enhancers, promoters, and binding sites of CTCF. The direction and level of correlation were mostly conserved when comparing results for HS and MM, which supports the idea of evolutionary pressure on the observed patterns. Comparing different annotations, the highest correlations were very consistently found for CTCF, especially in the case of flexibility-related DNA properties. Since CTCF is generally believed to be a key player for chromatin 3D organization, this might also be the first hint that DNA properties are relevant in that context. The correlations were also, in general, consistent between annotations, in the sense that a high correlation between a property and one annotation consistently implies a comparable level of correlation between that property and each other annotation analyzed. This result was expected to some extent, since all annotations that were analyzed are associated with transcription and a shared functional relationship often implies proximity and thus positional correlation (confirmed by our results). Nevertheless, we found stronger correlations between annotations and DNA properties compared to the correlations between different annotations. This provides a first hint that clustering of functional elements alone cannot explain the observed correlations to DNA properties. In a second approach, we examined DNA properties (profiles) around the same annotations, while masking influences provided from sequences within the annotations themselves. While we cannot strictly exclude that the results are influenced by the 1000 kbp resolution chosen for underlying genomic maps, we see the width and significance of the identified peaks (see Figure 2) as indicators that the observed patterns are sufficiently robust. Otherwise, it would not have been possible to differentiate between relevant influences from sequence patterns within annotations (which might still exist) and artifacts resulting from proximity between the elements discussed above. We found significant associations between a variety of DNA properties and annotations, often in the form of strong peaks of properties that were centered at the positions of annotations. While some associations identified with the former approach could be confirmed, the results for many properties were inconclusive or inconsistent between HS and MM. These include most minor or major groove-related properties and also the persistence length. In contrast, we still observed significant results for other flexibility-related properties (e.g., Shift stiffness). Remarkable are the observations of very consistent results (between HS and MM as well as between different models), especially for the Twist and even more so for the Roll property, both of which were known to be the most influential on important DNA features, such as DNA flexibility and histone occupancy. Consistency or conservation between HS and MM again supports the idea of evolutionary pressure, while consistency between models supports the reliability of our results. Our findings are consistent with former findings that implied a potential influence of these properties on transcription [13,14,15] and, since we also found relations to CTCF and TAD borders, also support a potential influence on chromatin 3D organization.

To identify specific sequence features that potentially cause the observed patterns around annotations, we directly derived the influences of the local dinucleotide content and the content of repeats (TRs and transposons) using the same models for DNA properties. We found that the relations between the dinucleotide content and properties around annotations are complex in the sense that, often, many dinucleotides have considerable influence on a single property and strongly influence its local value in opposing directions (often 40% of the total value and more). This is consistent with our recent observation that dinucleotide contents moderate each other’s influences on DNA properties on a chromosomal level [54] in many eukaryotes. It also implies that small changes in the dinucleotide content might result in considerable changes in influenced properties if they are not compensated for by fine-tuned changes in the content of other dinucleotides. If major functions like 3D organization or transcription would be affected by such uncompensated changes to DNA properties (e.g., by changing energetic costs of certain chromatin configurations), evolutionary pressure to maintain beneficial conditions for the formation of functional 3D structures might explain former findings that constant ratios between certain dinucleotide contents were conserved in eukaryotes [54]. Influences of repeats on DNA properties around annotations were found to be less prominent compared to influences of dinucleotide contents, which is expected considering that only a fraction of dinucleotides are part of a repeat while each repeat consists completely of dinucleotides. The highest influence of a repeat was found for transposons. The influence of L1 is consistently around 13–14%, which is even higher than the influence of most dinucleotides. For HS, the influence of Alu is on a comparable level, while it is only around 4% in MM. This reflects the much higher abundance of Alu in HS (or primates in general) compared to other animals [55]. The generally high influences of transposons might imply relevance for associated functions. Especially, we would expect significant changes in the influenced properties when the Alu or L1 contents change, which might lead to changes in 3D conformation and transcriptional activity. The much higher abundance of Alu in HS might therefore be associated with differences to MM in gene expression. While a test of this hypothesis is out of the scope of this study, our findings might provide hints to a mechanism of how Alu expansion in primates could lead to differences in phenotypes (e.g., higher intelligence). While the influences of transposons are mostly constant over different properties and annotations, influences of TRs, while on average smaller, are often more specific. It is again very remarkable that peaks of the Roll property are the ones that are most sensitive to changes in TRs (also for dinucleotides). This supports the outstanding *role of Roll*. Interesting is the relatively weak effect of (A)_n_ and (T)_n_ on Roll. (A)_n_ and (T)_n_, often referred to as Poly-A, were known to be stiff and, therefore, determinants of linker-DNA [42,56]. We would thus expect a substantial influence on the local flexibility and Roll, which is known to be associated with histone occupancy [12,16]. We see the expected effects to some extent for AA/TT dinucleotides but not for TRs. Remarkably, predictions of the influence of (A)_n_ on the Roll were also not consistent between different models for the Roll (see Figure 5). Roll (63) indicates a relatively high influence of 3–4% but most other models predict low values of around 1%. Since the model predictions are very consistent in most other cases, this might be a specific weakness of those models. Alternatively, the influence of (A)_n_ and (T)_n_ on the Roll and stiffness is in fact remarkably low around the analyzed functional annotations. It might then be plausible that, since the G+C contents around most annotations are known to be relatively high, the role of (A)_n_ in providing certain values of Roll or low flexibility is overtaken by that of (C)_n_ and (G)_n_, for which we observe a high influence on the Roll. (C)_n_/(G)_n_ might elevate the local G+C content of DNA, which could result in less flexible DNA [57]. Also, (AG)_n_ might play a major role here. Its influence on the Roll is even higher than that of (C)_n_ and (G)_n_. The high influence of (AG)_n_ and AG is especially remarkable because Alu sequences include high levels of AG. The influence of (AG)_n_ and AG is consistent between HS and MM, which indicates that the influence of many AG dinucleotides provided by Alu in HS is compensated by higher levels of AG outside of Alu within MM. This is a clear indication that evolutionary forces maintained specific dinucleotide contents after the Alu expansion in primates. Especially, those strong influences of TRs on the Roll support the hypothesis of a role of TRs in histone occupancy and thus 3D organization; however, given the high mutation rates of TRs [45] and the observed sensitivity of DNA properties, and considering variations in dinucleotide content, even the relatively small influence of some TRs might have a considerable influence on the 3D conformation.

In summary, our results strongly support the idea of a major role of dinucleotides and sequence repeats in 3D chromatin organization and transcription. Our findings that sequence repeats influence 3D chromatin organization are in-line with previous publications [37,38,39]. Therefore, the main finding of this study is strong support for an indirect mechanism determined by influences on local DNA properties being one possible pathway for this influence of repeats and dinucleotide contents on 3D chromatin organization.

## Figures and Tables

**Table 1:** Correlation between DNA properties and functional annotations: Representative selection of correlation values between genomic maps of functional annotations, derived based on GenBank files [47] and Ensembl data files, and genomic maps of DNA properties, derived based on dinucleotide maps, using models from the Dinucleotide Properties Genome Browser [52] (see Table S1 for complete dataset). Green color indicates positive correlation values, red color indicates negative correlation values (anti-correlation).

Property Model	Genes (HS)	Genes (MM)	Promoter (HS)	Promoter (MM)	Enhancer (HS)	Enhancer (MM)	CTCF Binding Site (HS)	CTCF Binding Site (MM)
Twist (1)	−0.37 ± 0.17	−0.44 ± 0.17	−0.57 ± 0.19	−0.48 ± 0.14	−0.21 ± 0.16	−0.54 ± 0.11	−0.42 ± 0.24	−0.63 ± 0.12
Twist (88)	−0.5 ± 0.17	−0.47 ± 0.14	−0.56 ± 0.16	−0.45 ± 0.18	−0.46 ± 0.18	−0.6 ± 0.15	−0.76 ± 0.1	−0.72 ± 0.1
Twist (61)	−0.47 ± 0.2	−0.44 ± 0.22	−0.63 ± 0.18	−0.41 ± 0.17	−0.41 ± 0.17	−0.38 ± 0.13	−0.56 ± 0.17	−0.57 ± 0.16
Roll (63)	0.54 ± 0.15	0.54 ± 0.14	0.55 ± 0.12	0.5 ± 0.17	0.42 ± 0.15	0.57 ± 0.1	0.74 ± 0.12	0.68 ± 0.09
Roll (90)	0.51 ± 0.16	0.52 ± 0.24	0.63 ± 0.16	0.51 ± 0.18	0.39 ± 0.16	0.59 ± 0.15	0.76 ± 0.06	0.65 ± 0.13
Roll (94)	0.57 ± 0.15	0.49 ± 0.15	0.54 ± 0.16	0.45 ± 0.14	0.35 ± 0.13	0.53 ± 0.16	0.8 ± 0.08	0.71 ± 0.07
Tilt (62)	0.47 ± 0.14	0.46 ± 0.12	0.5 ± 0.13	0.48 ± 0.14	0.36 ± 0.17	0.52 ± 0.19	0.71 ± 0.1	0.65 ± 0.13
Tilt (89)	−0.44 ± 0.17	−0.52 ± 0.18	−0.52 ± 0.15	−0.53 ± 0.14	−0.39 ± 0.14	−0.58 ± 0.07	−0.73 ± 0.1	−0.66 ± 0.11
Tilt (93)	0.49 ± 0.16	0.47 ± 0.19	0.61 ± 0.15	0.55 ± 0.17	0.36 ± 0.12	0.56 ± 0.12	0.75 ± 0.08	0.67 ± 0.1
Slide (28)	0.4 ± 0.2	0.45 ± 0.17	0.57 ± 0.17	0.37 ± 0.14	0.3 ± 0.14	0.42 ± 0.21	0.58 ± 0.16	0.53 ± 0.18
Slide (65)	0.46 ± 0.16	0.49 ± 0.15	0.47 ± 0.14	0.51 ± 0.14	0.41 ± 0.13	0.63 ± 0.11	0.73 ± 0.11	0.71 ± 0.09
Slide (91)	0.5 ± 0.17	0.52 ± 0.14	0.52 ± 0.16	0.48 ± 0.16	0.38 ± 0.14	0.61 ± 0.12	0.73 ± 0.1	0.67 ± 0.11
Shift (30)	−0.42 ± 0.17	−0.45 ± 0.15	−0.51 ± 0.21	−0.48 ± 0.16	−0.32 ± 0.16	−0.69 ± 0.11	−0.75 ± 0.09	−0.68 ± 0.09
Shift (64)	0.38 ± 0.18	0.26 ± 0.2	0.39 ± 0.15	0.22 ± 0.2	0.36 ± 0.18	0.4 ± 0.15	0.61 ± 0.13	0.48 ± 0.16
Shift (95)	−0.32 ± 0.22	−0.43 ± 0.17	−0.5 ± 0.13	−0.44 ± 0.11	−0.45 ± 0.13	−0.51 ± 0.12	−0.64 ± 0.07	−0.57 ± 0.12
Rise (3)	0.42 ± 0.15	0.46 ± 0.16	0.54 ± 0.18	0.5 ± 0.16	0.37 ± 0.17	0.55 ± 0.16	0.76 ± 0.07	0.68 ± 0.09
Rise (32)	0.5 ± 0.16	0.48 ± 0.18	0.51 ± 0.17	0.45 ± 0.2	0.38 ± 0.16	0.62 ± 0.14	0.72 ± 0.17	0.67 ± 0.12
Rise (66)	0.44 ± 0.16	0.47 ± 0.21	0.57 ± 0.22	0.43 ± 0.14	0.39 ± 0.15	0.53 ± 0.14	0.75 ± 0.1	0.68 ± 0.1
Major Groove Width (7)	0.49 ± 0.14	0.49 ± 0.19	0.54 ± 0.14	0.53 ± 0.17	0.34 ± 0.17	0.58 ± 0.1	0.79 ± 0.1	0.66 ± 0.12
Major Groove Depth (8)	−0.42 ± 0.17	−0.49 ± 0.17	−0.52 ± 0.14	−0.48 ± 0.18	−0.27 ± 0.16	−0.57 ± 0.16	−0.71 ± 0.08	−0.68 ± 0.1
Minor Groove Width (11)	−0.38 ± 0.19	−0.5 ± 0.18	−0.56 ± 0.13	−0.49 ± 0.18	−0.34 ± 0.17	−0.52 ± 0.1	−0.72 ± 0.1	−0.63 ± 0.14
Minor Groove Depth (12)	0.3 ± 0.17	0.06 ± 0.17	0.37 ± 0.2	0.01 ± 0.16	0.26 ± 0.21	0.03 ± 0.2	0.52 ± 0.17	0.05 ± 0.17
Persistance Length (15)	0.44 ± 0.13	0.55 ± 0.14	0.52 ± 0.19	0.43 ± 0.12	0.39 ± 0.15	0.6 ± 0.15	0.76 ± 0.1	0.67 ± 0.12
Slide stiffness (67)	−0.53 ± 0.16	−0.51 ± 0.12	−0.54 ± 0.16	−0.51 ± 0.12	−0.38 ± 0.18	−0.61 ± 0.11	−0.74 ± 0.09	−0.72 ± 0.09
Shift stiffness (68)	0.34 ± 0.19	0.36 ± 0.18	0.4 ± 0.15	0.25 ± 0.17	0.29 ± 0.16	0.11 ± 0.18	0.41 ± 0.17	0.21 ± 0.2
Roll stiffness (69)	0.42 ± 0.19	0.48 ± 0.13	0.5 ± 0.12	0.47 ± 0.13	0.34 ± 0.19	0.68 ± 0.09	0.68 ± 0.13	0.71 ± 0.11
Tilt stiffness (70)	0.51 ± 0.12	0.54 ± 0.11	0.59 ± 0.1	0.46 ± 0.22	0.38 ± 0.14	0.55 ± 0.14	0.69 ± 0.13	0.61 ± 0.13
Twist stiffness (71)	−0.45 ± 0.2	−0.36 ± 0.17	−0.46 ± 0.18	−0.42 ± 0.22	−0.21 ± 0.15	−0.47 ± 0.2	−0.67 ± 0.12	−0.61 ± 0.11
Rise stiffness (107)	0.38 ± 0.17	0.4 ± 0.25	0.45 ± 0.15	0.49 ± 0.16	0.28 ± 0.19	0.47 ± 0.16	0.72 ± 0.11	0.57 ± 0.14

## Data Availability

All codes and scripts (including visualization) used for this article, as well as a manual, are available online at http://www.kip.uni-heidelberg.de/biophysik/software (accessed on 28 August 2025) or from an associated GitHub repository at https://github.com/Sievers-A/Oligo (accessed on 27 September 2021).

## Supplementary Materials

The following supporting information can be downloaded at: https://www.mdpi.com/article/10.3390/genes16091082/s1, Figure S1: Relative Influence of Dinucleotides/Repeats on DNA Properties around Functional Annotations; Table S1: Correlation between DNA Properties and Functional Annotations; Table S2: Correlation of Functional Annotations; Table S3: Peaks in DNA Property Profiles around Functional Elements; Table S4: Correlation between DNA Properties and Functional Annotations (10 kbp Resolution); Table S5: Correlation between DNA Properties and Functional Annotations (1 Mbp Resolution).

## Abbreviations

The following abbreviations are used in this manuscript:

DNA	Deoxyribonucleic Aacid
HS	Homo sapiens
MM	Mus musculus
TR	Tandem repeat
L1	Line 1
CTCF	CCCTC-binding factor
TAD	Topologically associating domain
TF	Transcription factor
