# Supplementary material for: Repeats Influence Structural DNA Properties Around Functional Annotations Associated with 3D Organization and Transcription
Source: Genes (Basel). 2025 Sep 15;16(9):1082. doi: 10.3390/genes16091082 (PMC12470155; doi:10.3390/genes16091082)
Supplement: Supplementary file 1 [file genes-16-01082-s001.zip › genes-3824925-supplementary.pdf]

Table S1: Correlation between DNA Properties and Functional Annotations: Correlation values between genomic maps of functional annotations, derived based on from GenBank files [47], and Ensemble data files and genomic maps of DNA properties, derived based on dinucleotide maps, using models from the Dinucleotide Properties Genome Browser [52].

model	genes (HS)	genes (MM)	Promoter (HS)	Promoter (MM)	Enhancer (HS)	Enhancer (MM)	CTCF Binding Site (HS)	CTCF Binding Site (MM)
Twist (1)	-0.37 ± 0.17	-0.44 ± 0.17	-0.57 ± 0.19	-0.48 ± 0.14	-0.21 ± 0.16	-0.54 ± 0.11	-0.42 ± 0.24	-0.63 ± 0.12
Twist (DNA-protein complex) (26)	-0.4 ± 0.15	-0.48 ± 0.12	-0.55 ± 0.17	-0.46 ± 0.18	-0.36 ± 0.12	-0.62 ± 0.13	-0.75 ± 0.08	-0.63 ± 0.14
Twist (DNA-protein complex) (37)	-0.51 ± 0.15	-0.47 ± 0.13	-0.52 ± 0.12	-0.47 ± 0.15	-0.36 ± 0.15	-0.58 ± 0.16	-0.75 ± 0.1	-0.68 ± 0.07
Twist (88)	-0.5 ± 0.17	-0.47 ± 0.14	-0.56 ± 0.16	-0.45 ± 0.18	-0.46 ± 0.18	-0.6 ± 0.15	-0.76 ± 0.1	-0.72 ± 0.1
Twist (61)	-0.47 ± 0.2	-0.44 ± 0.22	-0.63 ± 0.18	-0.41 ± 0.17	-0.41 ± 0.17	-0.38 ± 0.13	-0.56 ± 0.17	-0.57 ± 0.16
Twist (98)	-0.5 ± 0.19	-0.44 ± 0.17	-0.52 ± 0.14	-0.44 ± 0.14	-0.39 ± 0.19	-0.56 ± 0.1	-0.72 ± 0.08	-0.66 ± 0.1
Twist (118)	-0.46 ± 0.16	-0.5 ± 0.19	-0.51 ± 0.2	-0.46 ± 0.12	-0.38 ± 0.13	-0.61 ± 0.16	-0.73 ± 0.13	-0.69 ± 0.11
Twist (120)	-0.44 ± 0.19	-0.42 ± 0.14	-0.51 ± 0.16	-0.49 ± 0.14	-0.37 ± 0.16	-0.64 ± 0.11	-0.76 ± 0.1	-0.7 ± 0.13
Twist (92)	-0.41 ± 0.19	-0.57 ± 0.18	-0.66 ± 0.09	-0.53 ± 0.17	-0.34 ± 0.15	-0.5 ± 0.17	-0.72 ± 0.15	-0.7 ± 0.08
Twist_twist (39)	-0.36 ± 0.14	-0.28 ± 0.18	-0.45 ± 0.13	-0.38 ± 0.11	-0.28 ± 0.17	-0.63 ± 0.12	-0.62 ± 0.14	-0.58 ± 0.13
Twist_tilt (42)	-0.48 ± 0.19	-0.34 ± 0.23	-0.58 ± 0.14	-0.43 ± 0.12	-0.36 ± 0.15	-0.6 ± 0.14	-0.73 ± 0.1	-0.69 ± 0.14
Twist_shift (51)	-0.41 ± 0.16	-0.42 ± 0.11	-0.49 ± 0.17	-0.47 ± 0.18	-0.4 ± 0.17	-0.59 ± 0.13	-0.7 ± 0.11	-0.58 ± 0.12
Twist_slide (52)	-0.46 ± 0.2	-0.41 ± 0.17	-0.6 ± 0.15	-0.28 ± 0.18	-0.41 ± 0.15	-0.28 ± 0.16	-0.69 ± 0.1	-0.45 ± 0.1
Twist_rise (53)	-0.34 ± 0.16	-0.48 ± 0.11	-0.34 ± 0.18	-0.44 ± 0.09	-0.4 ± 0.17	-0.59 ± 0.12	-0.28 ± 0.17	-0.65 ± 0.09
Twist_roll (43)	-0.45 ± 0.17	-0.47 ± 0.15	-0.53 ± 0.19	-0.43 ± 0.19	-0.39 ± 0.17	-0.6 ± 0.12	-0.71 ± 0.13	-0.72 ± 0.09
Roll (DNA-protein complex) (25)	0.43 ± 0.18	0.46 ± 0.15	0.52 ± 0.15	0.53 ± 0.13	0.41 ± 0.15	0.57 ± 0.13	0.72 ± 0.11	0.62 ± 0.12
Roll (63)	0.54 ± 0.15	0.54 ± 0.14	0.55 ± 0.12	0.5 ± 0.17	0.42 ± 0.15	0.57 ± 0.1	0.74 ± 0.12	0.68 ± 0.09
Roll (90)	0.51 ± 0.16	0.52 ± 0.24	0.63 ± 0.16	0.51 ± 0.18	0.39 ± 0.16	0.59 ± 0.15	0.76 ± 0.06	0.65 ± 0.13
Roll (94)	0.57 ± 0.15	0.49 ± 0.15	0.54 ± 0.16	0.45 ± 0.14	0.35 ± 0.13	0.53 ± 0.16	0.8 ± 0.08	0.71 ± 0.07
Roll (116)	0.41 ± 0.14	0.42 ± 0.16	0.51 ± 0.17	0.47 ± 0.13	0.32 ± 0.14	0.6 ± 0.11	0.73 ± 0.1	0.66 ± 0.1
Roll (119)	0.4 ± 0.17	0.52 ± 0.1	0.52 ± 0.11	0.51 ± 0.15	0.42 ± 0.15	0.68 ± 0.1	0.69 ± 0.08	0.73 ± 0.08
Roll (DNA-protein complex) (84)	0.45 ± 0.16	0.42 ± 0.19	0.54 ± 0.12	0.46 ± 0.12	0.35 ± 0.13	0.6 ± 0.1	0.77 ± 0.1	0.65 ± 0.1
Roll_roll (41)	0.51 ± 0.14	0.45 ± 0.15	0.5 ± 0.12	0.49 ± 0.16	0.37 ± 0.13	0.61 ± 0.11	0.71 ± 0.1	0.68 ± 0.1
Roll_shift (57)	-0.47 ± 0.19	-0.45 ± 0.19	-0.55 ± 0.13	-0.46 ± 0.15	-0.37 ± 0.17	-0.61 ± 0.12	-0.75 ± 0.09	-0.67 ± 0.13
Roll_slide (58)	0.47 ± 0.19	0.46 ± 0.15	0.48 ± 0.13	0.48 ± 0.15	0.38 ± 0.11	0.66 ± 0.11	0.76 ± 0.09	0.68 ± 0.13
Roll_rise (59)	0.42 ± 0.17	0.52 ± 0.13	0.56 ± 0.12	0.46 ± 0.13	0.38 ± 0.14	0.61 ± 0.14	0.72 ± 0.08	0.74 ± 0.08
Tilt_roll (44)	-0.53 ± 0.14	-0.52 ± 0.12	-0.56 ± 0.12	-0.5 ± 0.15	-0.37 ± 0.16	-0.59 ± 0.16	-0.72 ± 0.11	-0.63 ± 0.11
Tilt (DNA-protein complex) (27)	-0.37 ± 0.19	-0.39 ± 0.23	-0.41 ± 0.21	-0.4 ± 0.16	-0.21 ± 0.15	-0.58 ± 0.16	-0.64 ± 0.15	-0.6 ± 0.14
Tilt (62)	0.47 ± 0.14	0.46 ± 0.12	0.5 ± 0.13	0.48 ± 0.14	0.36 ± 0.17	0.52 ± 0.19	0.71 ± 0.1	0.65 ± 0.13
Tilt (89)	-0.44 ± 0.17	-0.52 ± 0.18	-0.52 ± 0.15	-0.53 ± 0.14	-0.39 ± 0.14	-0.58 ± 0.07	-0.73 ± 0.1	-0.66 ± 0.11
Tilt (93)	0.49 ± 0.16	0.47 ± 0.19	0.61 ± 0.15	0.55 ± 0.17	0.36 ± 0.12	0.56 ± 0.12	0.75 ± 0.08	0.67 ± 0.1
Tilt (117)	-0.05 ± 0.11	0.0 ± 0.16	-0.0 ± 0.17	-0.06 ± 0.15	0.13 ± 0.19	-0.06 ± 0.21	-0.06 ± 0.17	0.01 ± 0.18
Tilt (DNA-protein complex) (83)	0.39 ± 0.13	0.34 ± 0.14	0.56 ± 0.16	0.4 ± 0.19	0.16 ± 0.15	0.49 ± 0.14	0.66 ± 0.14	0.6 ± 0.11
Tilt_tilt (40)	0.43 ± 0.12	0.39 ± 0.2	0.52 ± 0.18	0.48 ± 0.17	0.38 ± 0.16	0.45 ± 0.12	0.74 ± 0.08	0.58 ± 0.1
Tilt_shift (54)	-0.47 ± 0.12	-0.47 ± 0.16	-0.58 ± 0.16	-0.5 ± 0.17	-0.35 ± 0.13	-0.55 ± 0.12	-0.75 ± 0.09	-0.7 ± 0.09
Twist_tilt (42)	-0.48 ± 0.19	-0.34 ± 0.23	-0.58 ± 0.14	-0.43 ± 0.12	-0.36 ± 0.15	-0.6 ± 0.14	-0.73 ± 0.1	-0.69 ± 0.14

Tilt_rise (56)	0.4 ± 0.15	0.41 ± 0.24	0.47 ± 0.16	0.48 ± 0.12	0.38 ± 0.18	0.58 ± 0.11	0.69 ± 0.16	0.69 ± 0.11
Tilt_slide (55)	0.41 ± 0.17	0.48 ± 0.21	0.6 ± 0.17	0.52 ± 0.12	0.4 ± 0.14	0.51 ± 0.1	0.75 ± 0.1	0.66 ± 0.16
Slide (DNA-protein complex) (28)	0.4 ± 0.2	0.45 ± 0.17	0.57 ± 0.17	0.37 ± 0.14	0.3 ± 0.14	0.42 ± 0.21	0.58 ± 0.16	0.53 ± 0.18
Slide (65)	0.46 ± 0.16	0.49 ± 0.15	0.47 ± 0.14	0.51 ± 0.14	0.41 ± 0.13	0.63 ± 0.11	0.73 ± 0.11	0.71 ± 0.09
Slide (91)	0.5 ± 0.17	0.52 ± 0.14	0.52 ± 0.16	0.48 ± 0.16	0.38 ± 0.14	0.61 ± 0.12	0.73 ± 0.1	0.67 ± 0.11
Slide (96)	0.39 ± 0.19	0.45 ± 0.17	0.54 ± 0.16	0.45 ± 0.14	0.37 ± 0.14	0.59 ± 0.11	0.77 ± 0.08	0.69 ± 0.1
Slide (DNA-protein complex) (86)	0.38 ± 0.15	0.34 ± 0.16	0.39 ± 0.15	0.39 ± 0.18	0.35 ± 0.14	0.57 ± 0.14	0.58 ± 0.18	0.57 ± 0.12
Slide_slide (46)	-0.21 ± 0.2	-0.29 ± 0.19	-0.18 ± 0.19	-0.33 ± 0.11	-0.28 ± 0.15	-0.62 ± 0.1	-0.29 ± 0.17	-0.55 ± 0.14
Slide_rise (50)	-0.35 ± 0.17	-0.36 ± 0.18	-0.37 ± 0.16	-0.27 ± 0.19	-0.41 ± 0.13	-0.28 ± 0.14	-0.56 ± 0.15	-0.31 ± 0.2
Twist_slide (52)	-0.46 ± 0.2	-0.41 ± 0.17	-0.6 ± 0.15	-0.28 ± 0.18	-0.41 ± 0.15	-0.28 ± 0.16	-0.69 ± 0.1	-0.45 ± 0.1
Roll_slide (58)	0.47 ± 0.19	0.46 ± 0.15	0.48 ± 0.13	0.48 ± 0.15	0.38 ± 0.11	0.66 ± 0.11	0.76 ± 0.09	0.68 ± 0.13
Shift_slide (48)	-0.33 ± 0.18	-0.31 ± 0.13	-0.34 ± 0.14	-0.3 ± 0.18	-0.3 ± 0.23	-0.57 ± 0.18	-0.56 ± 0.12	-0.59 ± 0.11
Shift (DNA-protein complex) (30)	-0.42 ± 0.17	-0.45 ± 0.15	-0.51 ± 0.21	-0.48 ± 0.16	-0.32 ± 0.16	-0.69 ± 0.11	-0.75 ± 0.09	-0.68 ± 0.09
Shift (64)	0.38 ± 0.18	0.26 ± 0.2	0.39 ± 0.15	0.22 ± 0.2	0.36 ± 0.18	0.4 ± 0.15	0.61 ± 0.13	0.48 ± 0.16
Shift (95)	-0.32 ± 0.22	-0.43 ± 0.17	-0.5 ± 0.13	-0.44 ± 0.11	-0.45 ± 0.13	-0.51 ± 0.12	-0.64 ± 0.07	-0.57 ± 0.12
Shift (DNA-protein complex) (85)	0.46 ± 0.17	0.42 ± 0.18	0.51 ± 0.16	0.54 ± 0.12	0.32 ± 0.13	0.66 ± 0.11	0.74 ± 0.13	0.72 ± 0.09
Shift_shift (45)	0.46 ± 0.18	0.47 ± 0.31	0.61 ± 0.16	0.48 ± 0.17	0.41 ± 0.16	0.41 ± 0.15	0.7 ± 0.11	0.57 ± 0.12
Twist_shift (51)	-0.41 ± 0.16	-0.42 ± 0.11	-0.49 ± 0.17	-0.47 ± 0.18	-0.4 ± 0.17	-0.59 ± 0.13	-0.7 ± 0.11	-0.58 ± 0.12
Tilt_shift (54)	-0.47 ± 0.12	-0.47 ± 0.16	-0.58 ± 0.16	-0.5 ± 0.17	-0.35 ± 0.13	-0.55 ± 0.12	-0.75 ± 0.09	-0.7 ± 0.09
Roll_shift (57)	-0.47 ± 0.19	-0.45 ± 0.19	-0.55 ± 0.13	-0.46 ± 0.15	-0.37 ± 0.17	-0.61 ± 0.12	-0.75 ± 0.09	-0.67 ± 0.13
Shift_rise (49)	0.42 ± 0.17	0.26 ± 0.16	0.6 ± 0.15	0.1 ± 0.22	0.22 ± 0.18	-0.1 ± 0.1	0.66 ± 0.13	0.09 ± 0.15
Rise (3)	0.42 ± 0.15	0.46 ± 0.16	0.54 ± 0.18	0.5 ± 0.16	0.37 ± 0.17	0.55 ± 0.16	0.76 ± 0.07	0.68 ± 0.09
Rise (DNA-protein complex) (32)	0.5 ± 0.16	0.48 ± 0.18	0.51 ± 0.17	0.45 ± 0.2	0.38 ± 0.16	0.62 ± 0.14	0.72 ± 0.17	0.67 ± 0.12
Rise (66)	0.44 ± 0.16	0.47 ± 0.21	0.57 ± 0.22	0.43 ± 0.14	0.39 ± 0.15	0.53 ± 0.14	0.75 ± 0.1	0.68 ± 0.1
Rise (97)	0.48 ± 0.16	0.52 ± 0.18	0.55 ± 0.18	0.51 ± 0.16	0.41 ± 0.11	0.59 ± 0.13	0.72 ± 0.13	0.71 ± 0.1
Rise (DNA-protein complex) (87)	0.48 ± 0.17	0.46 ± 0.14	0.59 ± 0.15	0.51 ± 0.12	0.3 ± 0.13	0.58 ± 0.13	0.74 ± 0.07	0.72 ± 0.08
Rise_rise (47)	-0.45 ± 0.25	-0.55 ± 0.15	-0.64 ± 0.13	-0.54 ± 0.15	-0.4 ± 0.15	-0.57 ± 0.1	-0.71 ± 0.12	-0.68 ± 0.1
Slide_rise (50)	-0.35 ± 0.17	-0.36 ± 0.18	-0.37 ± 0.16	-0.27 ± 0.19	-0.41 ± 0.13	-0.28 ± 0.14	-0.56 ± 0.15	-0.31 ± 0.2
Twist_rise (53)	-0.34 ± 0.16	-0.48 ± 0.11	-0.34 ± 0.18	-0.44 ± 0.09	-0.4 ± 0.17	-0.59 ± 0.12	-0.28 ± 0.17	-0.65 ± 0.09
Tilt_rise (56)	0.4 ± 0.15	0.41 ± 0.24	0.47 ± 0.16	0.48 ± 0.12	0.38 ± 0.18	0.58 ± 0.11	0.69 ± 0.16	0.69 ± 0.11
Roll_rise (59)	0.42 ± 0.17	0.52 ± 0.13	0.56 ± 0.12	0.46 ± 0.13	0.38 ± 0.14	0.61 ± 0.14	0.72 ± 0.08	0.74 ± 0.08
Bend (4)	-0.42 ± 0.19	-0.43 ± 0.19	-0.5 ± 0.15	-0.48 ± 0.18	-0.37 ± 0.14	-0.59 ± 0.12	-0.7 ± 0.12	-0.67 ± 0.1
Tip (5)	-0.38 ± 0.18	-0.42 ± 0.22	-0.53 ± 0.15	-0.47 ± 0.11	-0.35 ± 0.15	-0.61 ± 0.08	-0.74 ± 0.12	-0.61 ± 0.17
Inclination (6)	-0.04 ± 0.16	0.05 ± 0.17	-0.03 ± 0.13	-0.02 ± 0.18	0.01 ± 0.16	-0.06 ± 0.15	-0.03 ± 0.17	-0.03 ± 0.18
Propeller Twist (20)	0.45 ± 0.15	0.4 ± 0.17	0.5 ± 0.15	0.47 ± 0.17	0.41 ± 0.11	0.63 ± 0.06	0.69 ± 0.15	0.7 ± 0.09
Stacking energy (2)	0.44 ± 0.16	0.35 ± 0.2	0.54 ± 0.2	0.17 ± 0.18	0.29 ± 0.12	0.2 ± 0.2	0.69 ± 0.12	0.33 ± 0.24
Stacking energy (33)	-0.5 ± 0.17	-0.48 ± 0.17	-0.52 ± 0.15	-0.48 ± 0.14	-0.32 ± 0.19	-0.61 ± 0.1	-0.73 ± 0.1	-0.7 ± 0.1
Stacking energy (60)	0.46 ± 0.15	0.5 ± 0.24	0.59 ± 0.13	0.45 ± 0.17	0.34 ± 0.16	0.55 ± 0.11	0.77 ± 0.09	0.61 ± 0.11
Stacking energy (109)	0.54 ± 0.18	0.4 ± 0.22	0.51 ± 0.12	0.46 ± 0.16	0.39 ± 0.18	0.65 ± 0.09	0.72 ± 0.11	0.69 ± 0.08
Melting Temperature (16)	0.48 ± 0.14	0.46 ± 0.13	0.61 ± 0.13	0.54 ± 0.11	0.35 ± 0.17	0.63 ± 0.11	0.75 ± 0.09	0.69 ± 0.1

Melting Temperature (108)	0.46 ± 0.15	0.43 ± 0.17	0.57 ± 0.15	0.51 ± 0.15	0.39 ± 0.12	0.65 ± 0.11	0.67 ± 0.14	0.7 ± 0.13
Enthalpy (22)	-0.49 ± 0.17	-0.45 ± 0.17	-0.53 ± 0.13	-0.49 ± 0.12	-0.38 ± 0.14	-0.62 ± 0.15	-0.75 ± 0.07	-0.72 ± 0.08
Enthalpy (123)	-0.55 ± 0.18	-0.52 ± 0.14	-0.63 ± 0.12	-0.55 ± 0.13	-0.4 ± 0.16	-0.66 ± 0.11	-0.67 ± 0.15	-0.66 ± 0.12
Entropy (23)	-0.52 ± 0.15	-0.44 ± 0.22	-0.58 ± 0.15	-0.45 ± 0.16	-0.37 ± 0.15	-0.63 ± 0.1	-0.74 ± 0.1	-0.66 ± 0.1
Entropy (124)	-0.42 ± 0.17	-0.42 ± 0.19	-0.63 ± 0.12	-0.54 ± 0.09	-0.4 ± 0.15	-0.66 ± 0.12	-0.66 ± 0.11	-0.69 ± 0.09
Free energy (34)	-0.48 ± 0.14	-0.39 ± 0.2	-0.56 ± 0.12	-0.47 ± 0.17	-0.35 ± 0.15	-0.56 ± 0.15	-0.73 ± 0.12	-0.7 ± 0.1
Free energy (35)	-0.47 ± 0.15	-0.47 ± 0.17	-0.58 ± 0.13	-0.46 ± 0.16	-0.3 ± 0.14	-0.62 ± 0.1	-0.74 ± 0.11	-0.7 ± 0.1
Free energy (36)	-0.44 ± 0.15	-0.56 ± 0.18	-0.54 ± 0.16	-0.47 ± 0.13	-0.4 ± 0.13	-0.59 ± 0.11	-0.75 ± 0.08	-0.7 ± 0.12
Free energy (38)	-0.5 ± 0.19	-0.42 ± 0.19	-0.56 ± 0.16	-0.48 ± 0.16	-0.43 ± 0.13	-0.63 ± 0.14	-0.72 ± 0.12	-0.72 ± 0.08
Free energy (72)	-0.52 ± 0.15	-0.48 ± 0.16	-0.53 ± 0.17	-0.45 ± 0.16	-0.39 ± 0.15	-0.6 ± 0.12	-0.73 ± 0.07	-0.65 ± 0.08
Free energy (73)	-0.39 ± 0.17	-0.46 ± 0.15	-0.55 ± 0.16	-0.53 ± 0.1	-0.35 ± 0.15	-0.62 ± 0.13	-0.72 ± 0.14	-0.67 ± 0.11
Free energy (74)	-0.46 ± 0.19	-0.47 ± 0.14	-0.56 ± 0.15	-0.43 ± 0.16	-0.29 ± 0.13	-0.61 ± 0.13	-0.75 ± 0.09	-0.68 ± 0.13
Free energy (75)	-0.49 ± 0.13	-0.47 ± 0.17	-0.57 ± 0.13	-0.39 ± 0.13	-0.44 ± 0.15	-0.63 ± 0.14	-0.71 ± 0.12	-0.69 ± 0.12
Free energy (125)	-0.46 ± 0.13	-0.55 ± 0.09	-0.55 ± 0.18	-0.47 ± 0.18	-0.35 ± 0.19	-0.63 ± 0.1	-0.74 ± 0.11	-0.68 ± 0.1
Major Groove Width (7)	0.49 ± 0.14	0.49 ± 0.19	0.54 ± 0.14	0.53 ± 0.17	0.34 ± 0.17	0.58 ± 0.1	0.79 ± 0.1	0.66 ± 0.12
Major Groove Depth (8)	-0.42 ± 0.17	-0.49 ± 0.17	-0.52 ± 0.14	-0.48 ± 0.18	-0.27 ± 0.16	-0.57 ± 0.16	-0.71 ± 0.08	-0.68 ± 0.1
Major Groove Size (9)	-0.05 ± 0.21	0.04 ± 0.23	-0.02 ± 0.18	-0.06 ± 0.24	0.03 ± 0.22	-0.01 ± 0.19	0.03 ± 0.19	0.01 ± 0.19
Major Groove Distance (10)	-0.47 ± 0.15	-0.5 ± 0.15	-0.47 ± 0.16	-0.47 ± 0.15	-0.41 ± 0.15	-0.46 ± 0.13	-0.72 ± 0.09	-0.58 ± 0.13
Mobility to bend towards major groove (18)	-0.47 ± 0.14	-0.48 ± 0.17	-0.6 ± 0.14	-0.5 ± 0.14	-0.36 ± 0.18	-0.56 ± 0.15	-0.73 ± 0.14	-0.65 ± 0.13
Minor Groove Width (11)	-0.38 ± 0.19	-0.5 ± 0.18	-0.56 ± 0.13	-0.49 ± 0.18	-0.34 ± 0.17	-0.52 ± 0.1	-0.72 ± 0.1	-0.63 ± 0.14
Minor Groove Depth (12)	0.3 ± 0.17	0.06 ± 0.17	0.37 ± 0.2	0.01 ± 0.16	0.26 ± 0.21	0.03 ± 0.2	0.52 ± 0.17	0.05 ± 0.17
Minor Groove Size (13)	0.44 ± 0.19	0.43 ± 0.19	0.58 ± 0.14	0.44 ± 0.2	0.34 ± 0.16	0.61 ± 0.1	0.78 ± 0.07	0.71 ± 0.08
Minor Groove Distance (14)	-0.37 ± 0.21	-0.4 ± 0.16	-0.53 ± 0.14	-0.36 ± 0.21	-0.38 ± 0.16	-0.21 ± 0.17	-0.69 ± 0.12	-0.39 ± 0.17
Mobility to bend towards minor groove (19)	0.46 ± 0.17	0.45 ± 0.17	0.54 ± 0.07	0.44 ± 0.16	0.27 ± 0.11	0.57 ± 0.14	0.73 ± 0.1	0.73 ± 0.05
Probability contacting nucleosome core (17)	-0.46 ± 0.2	-0.44 ± 0.18	-0.49 ± 0.17	-0.49 ± 0.16	-0.31 ± 0.14	-0.6 ± 0.12	-0.73 ± 0.09	-0.63 ± 0.13
Persistance Length (15)	0.44 ± 0.13	0.55 ± 0.14	0.52 ± 0.19	0.43 ± 0.12	0.39 ± 0.15	0.6 ± 0.15	0.76 ± 0.1	0.67 ± 0.12
Slide stiffness (67)	-0.53 ± 0.16	-0.51 ± 0.12	-0.54 ± 0.16	-0.51 ± 0.12	-0.38 ± 0.18	-0.61 ± 0.11	-0.74 ± 0.09	-0.72 ± 0.09
Flexibility_slide (121)	-0.49 ± 0.12	-0.4 ± 0.19	-0.55 ± 0.11	-0.51 ± 0.17	-0.32 ± 0.18	-0.66 ± 0.12	-0.71 ± 0.1	-0.7 ± 0.09
Shift stiffness (68)	0.34 ± 0.19	0.36 ± 0.18	0.4 ± 0.15	0.25 ± 0.17	0.29 ± 0.16	0.11 ± 0.18	0.41 ± 0.17	0.21 ± 0.2
Flexibility_shift (122)	0.51 ± 0.14	0.46 ± 0.17	0.58 ± 0.15	0.46 ± 0.13	0.45 ± 0.12	0.68 ± 0.11	0.72 ± 0.11	0.7 ± 0.06
Roll stiffness (69)	0.42 ± 0.19	0.48 ± 0.13	0.5 ± 0.12	0.47 ± 0.13	0.34 ± 0.19	0.68 ± 0.09	0.68 ± 0.13	0.71 ± 0.11
Tilt stiffness (70)	0.51 ± 0.12	0.54 ± 0.11	0.59 ± 0.1	0.46 ± 0.22	0.38 ± 0.14	0.55 ± 0.14	0.69 ± 0.13	0.61 ± 0.13
Twist stiffness (71)	-0.45 ± 0.2	-0.36 ± 0.17	-0.46 ± 0.18	-0.42 ± 0.22	-0.21 ± 0.15	-0.47 ± 0.2	-0.67 ± 0.12	-0.61 ± 0.11
Rise stiffness (107)	0.38 ± 0.17	0.4 ± 0.25	0.45 ± 0.15	0.49 ± 0.16	0.28 ± 0.19	0.47 ± 0.16	0.72 ± 0.11	0.57 ± 0.14

Table S2: Correlation of Functional Annotations: Correlation values between genomic maps of functional annotations

element 1	Element 2	correlation coefficient (HS)	correlation coefficient (MM)
genes	Enhancer	0.01 ± 0.13	0.2 ± 0.16
genes	Promoter	0.52 ± 0.16	0.43 ± 0.16
genes	CTCF Binding Site	0.41 ± 0.12	0.41 ± 0.14
Enhancer	Promoter	0.11 ± 0.13	0.25 ± 0.14
Enhancer	CTCF Binding Site	0.42 ± 0.11	0.59 ± 0.12
Promoter	CTCF Binding Site	0.55 ± 0.15	0.56 ± 0.13

Table S3: Peaks in DNA Property Profiles around Functional Elements: Local profiles of DNA properties around functional elements were derived based on genomic maps with 1000 bp resolution. Shown are the width, height and significance of central peaks called within these profiles (negative significance indicates values lower than expected).

element	model	peak width (MM) [kbp]	peak height (MM) [1/kbp]	significance (MM)	reference height (MM) [1/kbp]	peak width (HS) [kbp]	peak height (HS) [1/kbp]	significance (HS)	reference height (HS) [1/kbp]
genes	Twist (1)	539	-446.02 ± 69.8	-2.3	-268.48	672	-717.14 ± 52.44	-5.2	-346.08
genes	Stacking energy (2)	131	37.08 ± 17.65	1.0	19.14	186	140.63 ± 34.02	3.3	25.53
genes	Rise (3)	406	199.62 ± 25.68	0.6	179.86	141	271.39 ± 34.42	0.9	230.79
genes	Bend (4)	504	-469.75 ± 53.01	0.4	-502.1	94	-575.54 ± 83.46	0.6	-641.36
genes	Tip (5)	417	-619.83 ± 80.99	0.4	-668.58	75	-697.51 ± 125.42	1.9	-1036.91
genes	Inclination (6)	472	67.76 ± 32.79	-0.2	75.09	774	37.98 ± 5.8	-0.9	44.54
genes	Major Groove Width (7)	435	949.15 ± 120.19	0.2	916.97	134	1242.47 ± 161.63	0.3	1174.63
genes	Major Groove Depth (8)	413	-211.47 ± 26.79	0.1	-213.91	113	-271.66 ± 38.2	0.0	-273.92
genes	Major Groove Size (9)	304	0.1 ± 0.04	2.7	-0.0	315	0.09 ± 0.04	2.2	-0.0
genes	Major Groove Distance (10)	819	-2.67 ± 0.27	-2.5	-1.8	103	-3.09 ± 0.53	-1.3	-2.3
genes	Minor Groove Width (11)	411	-278.59 ± 33.8	0.1	-285.05	97	-348.21 ± 50.82	0.2	-364.79
genes	Minor Groove Depth (12)	227	6.34 ± 0.42	3.5	4.06	9	5.57 ± 0.97	0.3	5.2
genes	Minor Groove Size (13)	458	359.84 ± 45.52	0.0	359.74	136	460.51 ± 58.12	0.0	460.43
genes	Minor Groove Distance (14)	706	-23.92 ± 12.85	-1.7	-1.59	80	-30.93 ± 9.67	-3.2	-0.01

genes	Persistance Length (15)	489	28606.25 ± 3627.92	0.1	28244.0	138	37179.76 ± 4858.28	0.2	36171.46
genes	Melting Temperature (16)	463	16008.01 ± 1946.84	-0.0	16095.82	134	19977.03 ± 2423.05	-0.2	20597.21
genes	Probability contacting nucleosome core (17)	275	-2302.22 ± 264.34	-1.2	-1875.69	226	-3088.32 ± 274.5	-1.6	-2411.81
genes	Mobility to bend towards major groove (18)	410	-40.75 ± 5.76	0.1	-41.85	129	-53.75 ± 7.09	0.0	-54.16
genes	Mobility to bend towards minor groove (19)	437	68.54 ± 8.96	-0.2	70.75	130	89.16 ± 11.81	-0.1	91.2
genes	Propeller Twist (20)	387	1011.59 ± 197.33	-1.7	1461.9	155	1343.06 ± 200.95	-1.8	1863.01
genes	Clash Strength (21)	475	307.94 ± 39.47	0.4	286.59	143	407.16 ± 51.98	0.6	366.99
genes	Enthalpy (22)	544	-1433.92 ± 159.66	-0.3	-1373.53	148	-1796.79 ± 205.71	-0.1	-1760.5
genes	Entropy (23)	593	-3121.38 ± 339.47	-0.3	-2979.27	154	-3918.58 ± 443.33	-0.1	-3819.85
genes	Shift (RNA) (24)	415	48.48 ± 6.22	-1.1	58.69	115	59.13 ± 6.63	-1.4	74.88
genes	Roll (DNA-protein complex) (25)	396	572.96 ± 93.5	-1.2	728.04	100	752.75 ± 127.44	-1.1	926.6
genes	Twist (DNA-protein complex) (26)	328	-289.61 ± 48.81	0.7	-334.56	103	-417.7 ± 74.65	1.1	-519.77
genes	Tilt (DNA-protein complex) (27)	89	-130.8 ± 28.78	0.6	-153.09	388	-193.28 ± 20.03	0.1	-196.03
genes	Slide (DNA-protein complex) (28)	842	57.23 ± 3.26	1.2	48.59	58	59.11 ± 4.7	-0.3	62.42
genes	Hydrophilicity (RNA) (29)	394	20.91 ± 8.57	0.1	19.25	160	13.5 ± 2.79	0.0	13.41
genes	Shift (DNA-protein complex) (30)	419	-16.72 ± 4.36	0.6	-19.77	373	-23.92 ± 3.83	0.3	-25.23
genes	Hydrophilicity (RNA) (31)	353	35.26 ± 3.59	0.2	34.3	119	45.84 ± 6.71	0.1	45.17
genes	Rise (DNA-protein complex) (32)	408	26.33 ± 3.95	-0.6	29.67	131	34.62 ± 4.81	-0.5	37.85
genes	Stacking energy (33)	436	-1771.06 ± 213.7	0.1	-1817.32	124	-2185.7 ± 259.8	0.4	-2323.98
genes	Free energy (34)	456	-375.64 ± 46.88	-0.0	-374.47	137	-475.01 ± 57.88	0.1	-479.29
genes	Free energy (35)	515	-584.81 ± 63.98	-0.7	-520.13	151	-731.67 ± 82.5	-0.5	-667.89
genes	Free energy (36)	459	-343.89 ± 42.18	-0.0	-340.89	137	-432.41 ± 51.91	0.0	-436.09
genes	Twist (DNA-protein complex) (37)	329	-161.99 ± 34.38	-0.3	-151.19	200	-278.53 ± 45.75	-1.6	-193.96
genes	Free energy (38)	463	-398.54 ± 48.55	0.0	-400.99	134	-497.55 ± 60.46	0.2	-513.13
genes	Twist_twist (39)	44	-0.84 ± 0.14	1.4	-1.13	33	-0.85 ± 0.11	4.0	-1.75
genes	Tilt_tilt (40)	756	0.73 ± 0.07	0.8	0.65	108	0.94 ± 0.15	0.6	0.83
genes	Roll_roll (41)	614	0.26 ± 0.04	0.7	0.23	123	0.27 ± 0.07	-0.0	0.27
genes	Twist_tilt (42)	272	-0.86 ± 0.17	0.9	-1.05	118	-1.26 ± 0.2	0.3	-1.34
genes	Twist_roll (43)	452	-1.24 ± 0.17	0.1	-1.26	139	-1.61 ± 0.21	0.0	-1.62
genes	Tilt_roll (44)	338	-0.88 ± 0.13	-0.3	-0.83	124	-1.2 ± 0.17	-0.6	-1.07
genes	Shift_shift (45)	896	42.83 ± 4.46	4.2	21.17	329	50.93 ± 7.47	2.6	27.73
genes	Slide_slide (46)	77	-24.73 ± 3.43	1.9	-35.33	584	-17.34 ± 1.39	6.1	-44.61
genes	Rise_rise (47)	722	-186.97 ± 18.5	-3.0	-116.2	381	-237.85 ± 21.57	-3.0	-149.33
genes	Shift_slide (48)	56	9.56 ± 2.21	8.4	-17.4	8	8.66 ± 2.1	11.4	-21.72

genes	Shift_rise (49)	590	20.94 ± 1.45	7.0	8.29	437	25.94 ± 2.66	4.2	10.99
genes	Slide_rise (50)	362	21.52 ± 4.5	1.1	16.04	21	20.78 ± 5.41	-0.0	20.91
genes	Twist_shift (51)	115	-2.97 ± 0.91	1.1	-4.15	74	-4.34 ± 1.11	0.5	-4.95
genes	Twist_slide (52)	948	-11.45 ± 0.85	-4.8	-6.02	203	-13.86 ± 1.55	-3.2	-7.77
genes	Twist_rise (53)	898	-1.82 ± 0.26	-0.1	-1.79	88	-0.56 ± 0.14	0.7	-0.8
genes	Tilt_shift (54)	507	-26.26 ± 3.03	-0.4	-24.44	138	-33.44 ± 4.08	-0.4	-31.32
genes	Tilt_slide (55)	476	2.28 ± 0.28	1.0	1.92	145	3.19 ± 0.41	1.4	2.46
genes	Tilt_rise (56)	195	14.07 ± 3.55	-1.7	21.91	120	21.14 ± 3.41	-1.5	27.8
genes	Roll_shift (57)	339	-2.86 ± 0.58	-0.0	-2.85	155	-4.15 ± 0.69	-0.6	-3.64
genes	Roll_slide (58)	438	5.6 ± 0.89	-1.3	7.3	123	7.01 ± 1.09	-1.5	9.31
genes	Roll_rise (59)	316	15.43 ± 2.14	0.3	14.63	208	20.72 ± 2.13	0.6	18.75
genes	Stacking energy (60)	422	556.81 ± 65.2	1.7	415.91	128	761.01 ± 101.99	1.8	533.58
genes	Twist (61)	1009	-471.73 ± 32.11	-5.1	-243.17	360	-573.47 ± 42.95	-4.3	-312.17
genes	Tilt (62)	465	93.62 ± 9.75	-0.6	103.44	56	105.76 ± 17.47	-1.1	132.15
genes	Roll (63)	789	674.34 ± 65.66	0.8	593.77	151	889.06 ± 111.07	0.9	758.02
genes	Shift (64)	151	2.74 ± 1.15	-2.8	6.88	19	5.15 ± 2.1	-1.3	8.16
genes	Slide (65)	324	155.57 ± 27.3	-0.4	170.4	107	185.5 ± 33.64	-1.7	264.99
genes	Rise (66)	343	37.26 ± 4.67	0.9	31.94	133	48.69 ± 6.1	0.9	41.02
genes	Slide stiffness (67)	566	-163.32 ± 21.4	-0.6	-146.83	157	-215.79 ± 30.24	-0.7	-187.91
genes	Shift stiffness (68)	801	16.24 ± 9.2	1.7	0.26	42	6.57 ± 2.19	2.7	0.58
genes	Roll stiffness (69)	396	0.17 ± 0.02	-0.2	0.18	115	0.16 ± 0.02	-2.0	0.23
genes	Tilt stiffness (70)	707	1.53 ± 0.16	0.4	1.44	113	1.91 ± 0.3	0.2	1.84
genes	Twist stiffness (71)	140	-1.0 ± 0.1	-1.6	-0.78	189	-1.2 ± 0.1	-1.1	-1.01
genes	Free energy (72)	482	-464.18 ± 54.42	-0.2	-448.8	137	-580.09 ± 68.21	-0.1	-574.87
genes	Free energy (73)	472	-415.74 ± 50.27	-0.1	-410.92	135	-519.99 ± 62.98	0.1	-526.2
genes	Free energy (74)	447	-359.03 ± 44.63	0.1	-363.77	135	-449.49 ± 55.07	0.2	-465.72
genes	Free energy (75)	458	-362.49 ± 46.02	0.1	-368.55	133	-460.26 ± 59.13	0.1	-471.72
genes	GC content (76)	458	719.56 ± 91.02	0.0	719.48	136	920.95 ± 116.23	0.0	920.87
genes	Purine (AG) content (77)	476	-83.11 ± 44.07	-0.0	-83.11	517	-45.35 ± 8.15	0.0	-45.37
genes	Keto (GT) content (78)	411	91.85 ± 24.67	-0.2	99.54	707	-62.85 ± 9.4	0.2	-64.79
genes	Adenine content (79)	363	-335.31 ± 46.66	-0.0	-335.28	116	-443.6 ± 58.86	-0.0	-443.54
genes	Guanine content (80)	469	386.31 ± 44.87	0.0	386.26	130	473.92 ± 59.43	0.0	473.88
genes	Cytosine content (81)	433	333.25 ± 46.51	0.0	333.23	156	447.03 ± 57.02	0.0	446.99
genes	Thymine content (82)	454	-384.25 ±	-	-384.2	173	-477.35 ± 58.0	-0.0	-477.33

			45.42	0.0					
genes	Tilt (DNA-protein complex) (83)	169	175.42 ± 34.95	-0.8	210.42	285	257.76 ± 33.3	-0.3	270.5
genes	Roll (DNA-protein complex) (84)	386	627.52 ± 95.77	-0.4	682.44	127	845.15 ± 122.04	-0.2	871.51
genes	Shift (DNA-protein complex) (85)	464	10.11 ± 3.11	-0.5	11.84	460	13.6 ± 2.81	-1.4	18.64
genes	Slide (DNA-protein complex) (86)	183	22.77 ± 1.76	-1.0	26.67	4	16.59 ± 2.57	-2.8	31.81
genes	Rise (DNA-protein complex) (87)	419	33.4 ± 4.48	0.3	31.48	159	45.38 ± 5.49	0.7	40.33
genes	Twist (88)	545	-864.46 ± 100.9	-0.8	-757.55	155	-1113.06 ± 136.98	-0.8	-970.95
genes	Tilt (89)	585	-169.49 ± 25.36	-0.3	-158.27	141	-225.74 ± 37.76	-0.6	-201.01
genes	Roll (90)	646	898.21 ± 107.35	0.7	800.97	190	1293.71 ± 165.03	1.2	1027.7
genes	Slide (91)	366	138.46 ± 26.38	-1.0	171.98	138	184.53 ± 30.62	-0.9	219.74
genes	Twist (92)	728	-184.49 ± 34.59	-1.5	-128.01	395	-298.85 ± 50.21	-2.4	-165.2
genes	Tilt (93)	543	457.9 ± 59.18	0.8	394.15	142	623.44 ± 91.54	1.1	504.33
genes	Roll (94)	620	605.35 ± 92.5	0.6	539.16	233	937.29 ± 140.78	1.5	691.01
genes	Shift (95)	371	-11.89 ± 1.73	-5.8	-1.81	80	-14.52 ± 2.87	-4.3	-2.22
genes	Slide (96)	369	189.35 ± 33.6	-0.7	221.19	141	254.93 ± 39.88	-0.5	282.9
genes	Rise (97)	400	35.85 ± 4.81	0.5	32.39	198	46.41 ± 5.36	0.6	41.62
genes	Twist (98)	366	-197.33 ± 38.5	0.5	-223.54	112	-307.63 ± 53.27	-0.4	-282.47
genes	Wedge (99)	64	-82.53 ± 27.47	-1.7	-29.86	531	-200.97 ± 51.15	-0.0	-199.25
genes	Direction (100)	375	17100.69 ± 2371.04	0.8	14659.7	105	19496.61 ± 2334.15	0.3	18566.88
genes	Slide (RNA) (101)	461	-137.84 ± 16.27	-0.2	-133.92	128	-181.61 ± 22.34	-0.3	-171.41
genes	Rise (RNA) (102)	471	33.19 ± 3.31	1.3	26.99	119	44.84 ± 5.11	1.5	34.54
genes	Tilt (RNA) (103)	101	99.03 ± 21.01	-1.6	146.06	44	130.92 ± 34.0	-1.1	174.18
genes	Roll (RNA) (104)	364	727.0 ± 80.61	1.0	618.3	129	973.12 ± 117.49	1.2	778.17
genes	Twist (RNA) (105)	857	-131.13 ± 10.26	-0.8	-116.82	371	-114.83 ± 8.58	1.8	-148.29
genes	Stacking energy (RNA) (106)	675	324.58 ± 49.22	-0.7	375.23	135	468.79 ± 88.28	-0.1	479.75
genes	Rise stiffness (107)	269	107.46 ± 10.76	0.0	106.7	68	139.72 ± 17.93	0.1	136.76
genes	Melting Temperature (108)	464	46.18 ± 5.61	-0.0	46.41	134	57.64 ± 6.99	-0.2	59.39
genes	Stacking energy (109)	429	181.29 ± 21.68	-0.1	185.02	126	223.49 ± 25.78	-0.3	236.63
genes	Enthalpy (RNA) (110)	463	-1748.33 ± 230.8	0.2	-1812.51	132	-2249.48 ± 298.75	0.1	-2307.61
genes	Entropy (RNA) (111)	465	-3457.14 ± 459.42	0.3	-3639.78	127	-4440.6 ± 597.38	0.2	-4621.75
genes	Free energy (RNA) (112)	454	-662.35 ± 86.51	0.1	-671.2	136	-852.45 ± 110.74	0.0	-858.07

genes	Free energy (RNA) (113)	446	-712.95 ± 95.11	0.1	-729.08	136	-924.81 ± 122.0	0.0	-932.76
genes	Enthalpy (RNA) (114)	411	-1904.29 ± 258.18	0.3	-2000.01	125	-2478.56 ± 333.66	0.2	-2557.24
genes	Entropy (RNA) (115)	392	-3841.37 ± 526.15	0.3	-4098.58	120	-5010.92 ± 683.09	0.2	-5238.74
genes	Roll (116)	340	1128.46 ± 201.82	-1.1	1412.83	123	1572.33 ± 239.19	-0.7	1800.05
genes	Tilt (117)	502	123.76 ± 58.8	-0.0	126.68	551	75.06 ± 9.74	-0.5	81.34
genes	Twist (118)	429	-769.99 ± 107.78	0.2	-802.13	142	-1015.91 ± 131.82	0.1	-1026.23
genes	Roll (119)	634	352.77 ± 37.97	-1.0	415.17	107	371.49 ± 45.15	-2.1	528.11
genes	Twist (120)	430	-535.73 ± 101.12	1.3	-715.42	169	-700.66 ± 109.73	1.4	-912.83
genes	Flexibility_slide (121)	349	-1918.48 ± 305.73	0.7	-2189.79	132	-2485.25 ± 345.37	0.6	-2798.47
genes	Flexibility_shift (122)	678	869.46 ± 92.95	-0.6	957.0	149	1021.96 ± 110.96	-1.1	1218.37
genes	Enthalpy (123)	460	-619.15 ± 69.18	-0.1	-609.67	134	-743.29 ± 79.03	0.3	-780.49
genes	Entropy (124)	408	-666.32 ± 63.75	-0.2	-646.34	122	-733.3 ± 54.36	0.8	-827.13
genes	Free energy (125)	472	-415.74 ± 50.27	-0.1	-410.92	135	-519.99 ± 62.98	0.1	-526.2
Enhancer	Twist (1)	693	-111.89 ± 31.72	-1.8	-43.92	114	64.68 ± 62.82	0.9	2.4
Enhancer	Stacking energy (2)	37	26.01 ± 22.5	1.1	0.9	20	32.82 ± 28.0	1.1	2.56
Enhancer	Rise (3)	660	39.01 ± 13.23	0.2	36.17	32	65.47 ± 28.91	-0.1	68.58
Enhancer	Bend (4)	800	-110.47 ± 38.21	-0.1	-107.14	50	-259.98 ± 75.5	-0.5	-203.62
Enhancer	Tip (5)	742	-193.68 ± 68.97	-0.3	-169.16	53	-450.85 ± 126.63	-0.6	-340.82
Enhancer	Inclination (6)	977	-59.75 ± 30.88	-0.0	-58.79	837	-39.69 ± 27.74	-0.1	-36.07
Enhancer	Major Groove Width (7)	703	196.26 ± 67.48	0.1	189.81	39	373.95 ± 140.29	0.1	358.27
Enhancer	Major Groove Depth (8)	709	-45.11 ± 16.02	-0.0	-44.77	38	-97.3 ± 34.08	-0.3	-83.9
Enhancer	Major Groove Size (9)	104	0.18 ± 0.18	1.0	-0.0	204	-0.08 ± 0.07	-1.2	0.0
Enhancer	Major Groove Distance (10)	814	-0.93 ± 0.48	-1.1	-0.4	180	-2.53 ± 0.43	-3.6	-0.71
Enhancer	Minor Groove Width (11)	715	-60.49 ± 21.57	0.0	-60.64	43	-145.77 ± 45.82	-0.5	-112.74
Enhancer	Minor Groove Depth (12)	360	-1.51 ± 1.18	-1.5	0.37	144	4.28 ± 0.74	3.0	1.51
Enhancer	Minor Groove Size (13)	740	77.45 ± 25.82	-0.0	77.54	44	142.4 ± 51.14	0.0	142.36
Enhancer	Minor Groove Distance (14)	310	-14.66 ± 11.46	-1.2	-0.66	212	-48.75 ± 7.21	-6.8	0.03
Enhancer	Persistance Length (15)	760	6198.56 ± 2051.25	0.1	6037.76	43	11592.95 ± 4258.1	0.1	11071.48
Enhancer	Melting Temperature (16)	759	3333.98 ± 1142.81	-0.1	3453.64	45	6022.18 ± 2132.19	-0.1	6388.81
Enhancer	Probability contacting nucleosome core (17)	559	-304.98 ± 97.44	0.5	-381.61	12	-239.35 ± 202.33	0.3	-325.33
Enhancer	Mobility to bend towards major groove (18)	579	-9.31 ± 3.54	-0.2	-8.57	47	-17.67 ± 6.33	-0.1	-16.92

Enhancer	Mobility to bend towards minor groove (19)	713	15.1 ± 5.44	0.0	14.97	43	29.22 ± 10.52	0.1	28.47
Enhancer	Propeller Twist (20)	698	406.2 ± 138.34	0.5	316.11	66	559.47 ± 205.21	-0.2	616.32
Enhancer	Clash Strength (21)	735	68.61 ± 21.85	0.2	61.38	47	129.4 ± 44.67	0.3	112.47
Enhancer	Enthalpy (22)	842	-266.48 ± 85.18	0.2	-286.42	37	-401.11 ± 173.42	0.5	-532.57
Enhancer	Entropy (23)	879	-569.64 ± 178.55	0.2	-618.94	32	-790.73 ± 370.12	0.6	-1149.7
Enhancer	Shift (RNA) (24)	730	11.79 ± 3.94	-0.2	13.05	42	14.5 ± 6.17	-0.9	24.32
Enhancer	Roll (DNA-protein complex) (25)	674	204.52 ± 69.83	0.5	159.04	67	466.15 ± 124.09	1.0	310.54
Enhancer	Twist (DNA-protein complex) (26)	616	-110.0 ± 40.36	-0.4	-88.49	42	-211.72 ± 72.76	-0.4	-172.67
Enhancer	Tilt (DNA-protein complex) (27)	404	-39.31 ± 11.78	-0.5	-30.4	12	-22.65 ± 17.38	0.1	-25.28
Enhancer	Slide (DNA-protein complex) (28)	945	5.65 ± 3.05	-0.5	8.37	21	4.66 ± 3.16	-1.8	18.17
Enhancer	Hydrophilicity (RNA) (29)	748	-13.5 ± 8.49	-0.0	-13.12	537	-8.47 ± 6.6	0.0	-8.61
Enhancer	Shift (DNA-protein complex) (30)	713	-9.21 ± 2.97	-1.5	-3.97	146	-14.62 ± 3.95	-1.3	-8.18
Enhancer	Hydrophilicity (RNA) (31)	626	-15.38 ± 11.93	-0.0	-15.17	155	18.13 ± 5.87	0.4	15.23
Enhancer	Rise (DNA-protein complex) (32)	692	7.52 ± 2.55	0.3	6.63	61	14.38 ± 4.48	0.3	12.26
Enhancer	Stacking energy (33)	743	-371.11 ± 127.28	0.1	-393.07	45	-659.49 ± 229.74	0.2	-728.56
Enhancer	Free energy (34)	749	-79.52 ± 27.18	0.0	-80.18	44	-140.89 ± 50.81	0.1	-148.26
Enhancer	Free energy (35)	813	-96.63 ± 31.47	0.2	-106.17	32	-152.1 ± 67.89	0.4	-196.31
Enhancer	Free energy (36)	755	-70.3 ± 23.79	0.1	-72.64	43	-119.89 ± 45.33	0.2	-133.89
Enhancer	Twist (DNA-protein complex) (37)	558	-55.5 ± 20.46	-1.1	-30.23	39	-93.85 ± 40.27	-0.8	-57.81
Enhancer	Free energy (38)	758	-83.22 ± 28.53	0.1	-86.05	45	-150.41 ± 53.22	0.1	-159.18
Enhancer	Twist_twist (39)	449	-0.3 ± 0.1	-0.1	-0.29	30	-0.26 ± 0.13	1.4	-0.58
Enhancer	Tilt_tilt (40)	827	0.18 ± 0.08	0.4	0.14	60	0.46 ± 0.13	1.3	0.25
Enhancer	Roll_roll (41)	852	0.09 ± 0.03	0.9	0.06	67	0.25 ± 0.06	1.8	0.11
Enhancer	Twist_tilt (42)	561	-0.32 ± 0.12	-0.6	-0.23	56	-0.63 ± 0.2	-0.8	-0.44
Enhancer	Twist_roll (43)	739	-0.28 ± 0.1	-0.1	-0.27	43	-0.5 ± 0.19	-0.0	-0.5
Enhancer	Tilt_roll (44)	610	-0.25 ± 0.08	-0.7	-0.18	58	-0.53 ± 0.15	-1.0	-0.34
Enhancer	Shift_shift (45)	903	10.08 ± 5.48	1.4	2.22	140	24.71 ± 6.02	2.8	5.41
Enhancer	Slide_slide (46)	519	-10.11 ± 2.68	-0.7	-7.21	410	-11.26 ± 1.16	1.1	-16.79
Enhancer	Rise_rise (47)	912	-38.46 ± 10.84	-1.1	-23.3	171	-57.37 ± 14.76	-0.6	-43.12
Enhancer	Shift_slide (48)	467	-7.12 ± 2.59	-0.9	-4.45	32	-7.14 ± 2.88	0.6	-9.36
Enhancer	Shift_rise (49)	94	-2.05 ± 1.48	-1.4	0.13	32	2.25 ± 1.71	0.8	0.64
Enhancer	Slide_rise (50)	157	-8.37 ± 6.57	-1.6	2.37	166	-38.3 ± 5.49	-7.3	4.99

Enhancer	Twist_shift (51)	468	-2.17 ± 0.78	-1.1	-1.2	83	-4.73 ± 1.09	-1.8	-2.37
Enhancer	Twist_slide (52)	690	-2.17 ± 1.5	-0.6	-1.21	179	-4.45 ± 1.0	-1.8	-2.07
Enhancer	Twist_rise (53)	1023	-1.21 ± 0.41	-1.8	-0.26	720	-2.5 ± 0.14	-4.5	-1.01
Enhancer	Tilt_shift (54)	792	-5.16 ± 1.64	0.0	-5.2	45	-9.63 ± 3.47	-0.0	-9.52
Enhancer	Tilt_slide (55)	690	0.55 ± 0.18	0.6	0.4	52	1.01 ± 0.34	0.6	0.76
Enhancer	Tilt_rise (56)	530	7.22 ± 2.54	0.7	4.96	67	11.11 ± 3.51	0.3	9.76
Enhancer	Roll_shift (57)	607	-1.05 ± 0.37	-1.0	-0.63	77	-2.35 ± 0.65	-1.6	-1.15
Enhancer	Roll_slide (58)	756	1.84 ± 0.65	0.3	1.61	62	3.53 ± 1.08	0.3	3.04
Enhancer	Roll_rise (59)	600	3.16 ± 1.0	0.0	3.1	32	2.68 ± 1.77	-1.1	5.72
Enhancer	Stacking energy (60)	641	126.74 ± 48.01	0.7	85.05	49	272.74 ± 82.35	1.1	157.96
Enhancer	Twist (61)	960	-88.61 ± 47.83	-0.8	-50.37	314	-105.15 ± 19.91	-0.4	-90.86
Enhancer	Tilt (62)	774	21.9 ± 8.05	0.2	20.1	50	63.48 ± 16.39	1.0	41.75
Enhancer	Roll (63)	926	170.69 ± 60.82	0.7	114.95	90	309.29 ± 87.86	0.6	241.61
Enhancer	Shift (64)	478	2.69 ± 1.44	0.4	1.98	28	5.15 ± 1.94	0.4	4.18
Enhancer	Slide (65)	647	59.55 ± 20.87	0.6	43.06	63	121.58 ± 34.9	0.8	86.6
Enhancer	Rise (66)	618	6.77 ± 2.33	0.1	6.4	33	13.48 ± 5.13	0.2	12.04
Enhancer	Slide stiffness (67)	803	-44.62 ± 13.85	-0.8	-30.7	78	-95.71 ± 26.42	-1.1	-57.93
Enhancer	Shift stiffness (68)	139	7.73 ± 7.32	1.0	0.59	614	22.11 ± 1.62	13.1	0.68
Enhancer	Roll stiffness (69)	803	0.04 ± 0.02	0.3	0.04	132	0.08 ± 0.02	0.3	0.07
Enhancer	Tilt stiffness (70)	876	0.39 ± 0.15	0.4	0.31	59	0.97 ± 0.26	1.2	0.57
Enhancer	Twist stiffness (71)	530	-0.08 ± 0.03	1.1	-0.15	4	0.08 ± 0.06	2.7	-0.27
Enhancer	Free energy (72)	766	-89.68 ± 30.4	0.1	-94.3	42	-154.88 ± 58.67	0.2	-175.69
Enhancer	Free energy (73)	762	-84.25 ± 28.9	0.1	-86.63	43	-149.74 ± 55.04	0.1	-161.55
Enhancer	Free energy (74)	743	-74.85 ± 25.67	0.1	-77.69	41	-130.09 ± 48.69	0.2	-143.53
Enhancer	Free energy (75)	745	-78.38 ± 27.63	-0.0	-78.07	44	-147.4 ± 52.57	-0.0	-145.8
Enhancer	GC content (76)	739	155.02 ± 51.62	-0.0	155.08	44	284.79 ± 102.28	0.0	284.71
Enhancer	Purine (AG) content (77)	858	67.67 ± 40.06	-0.0	67.83	723	45.11 ± 33.9	0.0	45.06
Enhancer	Keto (GT) content (78)	1295	-90.22 ± 43.8	-0.1	-86.01	919	-48.93 ± 8.77	-2.9	-16.7
Enhancer	Adenine content (79)	545	-73.75 ± 32.94	-0.0	-73.73	54	-137.99 ± 51.36	-0.0	-137.95
Enhancer	Guanine content (80)	582	79.79 ± 26.93	-0.0	79.84	37	139.89 ± 51.71	0.0	139.82
Enhancer	Cytosine content (81)	925	95.95 ± 28.42	0.0	95.92	69	144.9 ± 50.76	0.0	144.9
Enhancer	Thymine content (82)	1172	-139.79 ± 39.99	-0.0	-139.67	76	-146.8 ± 51.67	-0.0	-146.76
Enhancer	Tilt (DNA-protein complex) (83)	525	50.78 ± 15.57	0.4	41.55	11	33.41 ± 29.01	-0.1	36.86
Enhancer	Roll (DNA-protein complex) (84)	654	174.89 ± 60.09	0.3	151.1	48	350.63 ± 112.3	0.5	278.58
Enhancer	Shift (DNA-protein complex) (85)	769	7.31 ± 2.19	1.8	2.63	123	8.29 ± 3.04	0.7	5.8
Enhancer	Slide (DNA-protein complex) (86)	680	5.89 ± 2.16	0.0	5.87	39	13.19 ± 3.15	-0.1	13.95

Enhancer	Rise (DNA-protein complex) (87)	674	6.97 ± 2.37	0.1	6.66	38	10.55 ± 4.65	-0.3	12.29
Enhancer	Twist (88)	814	-181.9 ± 57.4	-0.3	-155.37	52	-352.27 ± 114.94	-0.4	-293.11
Enhancer	Tilt (89)	809	-76.6 ± 23.23	-1.5	-36.19	167	-179.29 ± 34.4	-2.7	-68.78
Enhancer	Roll (90)	814	187.07 ± 67.74	0.3	162.33	33	239.55 ± 132.74	-0.4	304.78
Enhancer	Slide (91)	661	50.31 ± 18.02	0.6	36.08	58	95.46 ± 30.29	0.7	69.85
Enhancer	Twist (92)	880	-70.98 ± 22.95	-2.0	-22.21	111	-141.3 ± 43.47	-2.1	-44.0
Enhancer	Tilt (93)	765	129.67 ± 42.73	0.9	82.88	66	287.47 ± 78.57	1.4	155.51
Enhancer	Roll (94)	787	176.6 ± 57.12	0.9	113.67	54	313.35 ± 119.34	0.7	207.89
Enhancer	Shift (95)	548	-7.13 ± 3.27	-2.0	-0.68	265	-20.1 ± 2.24	-8.4	-1.05
Enhancer	Slide (96)	652	60.93 ± 21.59	0.5	45.9	49	111.31 ± 38.3	0.5	88.59
Enhancer	Rise (97)	678	6.93 ± 2.28	0.1	6.64	33	9.38 ± 4.56	-0.4	12.13
Enhancer	Twist (98)	606	-107.06 ± 37.16	-1.4	-46.75	113	-223.97 ± 49.12	-2.1	-103.84
Enhancer	Wedge (99)	334	-79.4 ± 42.61	-1.2	-24.1	4	135.37 ± 43.99	3.9	-56.68
Enhancer	Direction (100)	695	-5440.02 ± 3670.08	-0.3	-3852.75	80	4520.21 ± 1861.59	-0.2	5171.95
Enhancer	Slide (RNA) (101)	720	-27.73 ± 9.22	0.0	-27.94	33	-46.19 ± 18.88	0.3	-52.98
Enhancer	Rise (RNA) (102)	680	6.44 ± 2.52	0.2	5.65	37	10.42 ± 3.87	-0.0	10.68
Enhancer	Tilt (RNA) (103)	491	54.58 ± 20.75	0.5	41.23	44	114.86 ± 33.58	0.8	81.77
Enhancer	Roll (RNA) (104)	522	132.56 ± 50.57	0.2	121.33	24	179.07 ± 93.36	-0.4	225.2
Enhancer	Twist (RNA) (105)	682	-33.17 ± 24.58	-0.3	-25.45	446	-16.36 ± 5.49	2.0	-51.33
Enhancer	Stacking energy (RNA) (106)	877	109.79 ± 40.09	0.6	79.81	51	246.74 ± 80.97	1.0	149.48
Enhancer	Rise stiffness (107)	579	17.12 ± 7.48	-0.5	22.03	20	25.79 ± 14.93	-0.7	41.29
Enhancer	Melting Temperature (108)	759	9.61 ± 3.29	-0.1	9.96	45	17.34 ± 6.15	-0.1	18.42
Enhancer	Stacking energy (109)	736	37.17 ± 12.59	-0.1	39.97	41	62.29 ± 22.62	-0.3	74.07
Enhancer	Enthalpy (RNA) (110)	771	-434.03 ± 137.1	-0.1	-418.84	46	-775.4 ± 268.0	-0.2	-719.29
Enhancer	Entropy (RNA) (111)	786	-918.54 ± 270.35	-0.1	-870.84	49	-1581.02 ± 539.13	-0.2	-1446.52
Enhancer	Free energy (RNA) (112)	737	-150.18 ± 50.03	-0.1	-145.56	45	-281.9 ± 98.49	-0.1	-266.1
Enhancer	Free energy (RNA) (113)	723	-164.93 ± 55.61	-0.1	-157.93	45	-312.77 ± 108.98	-0.2	-290.2
Enhancer	Enthalpy (RNA) (114)	693	-449.29 ± 154.26	-0.1	-436.39	43	-868.47 ± 300.49	-0.2	-802.77
Enhancer	Entropy (RNA) (115)	685	-917.35 ± 318.39	-0.0	-897.93	43	-1793.38 ± 618.17	-0.2	-1652.86
Enhancer	Roll (116)	617	389.45 ± 139.0	0.5	306.63	55	708.98 ± 229.59	0.4	596.03
Enhancer	Tilt (117)	937	-99.86 ± 67.79	-0.0	-96.06	817	-58.01 ± 39.52	0.0	-59.53
Enhancer	Twist (118)	709	-183.67 ± 63.71	-0.1	-172.6	42	-311.02 ± 118.02	0.1	-319.56

Enhancer	Roll (119)	906	114.27 ± 31.51	0.6	84.44	172	226.02 ± 44.18	0.6	178.34
Enhancer	Twist (120)	731	-206.17 ± 72.15	-0.6	-152.46	72	-340.77 ± 110.78	-0.3	-296.31
Enhancer	Flexibility_slide (121)	647	-541.31 ± 194.61	-0.2	-477.84	48	-951.56 ± 327.46	-0.1	-887.26
Enhancer	Flexibility_shift (122)	920	248.39 ± 68.27	0.5	189.78	144	400.18 ± 99.06	-0.0	406.67
Enhancer	Enthalpy (123)	771	-115.06 ± 37.5	0.3	-130.41	43	-175.13 ± 67.66	0.6	-240.94
Enhancer	Entropy (124)	771	-100.81 ± 29.78	0.6	-133.64	35	-81.12 ± 42.15	1.7	-257.42
Enhancer	Free energy (125)	762	-84.25 ± 28.9	0.1	-86.63	43	-149.74 ± 55.04	0.1	-161.55
Promoter	Twist (1)	857	-261.99 ± 93.98	-1.7	-98.93	536	-970.83 ± 152.92	-2.6	-563.2
Promoter	Stacking energy (2)	195	41.76 ± 26.84	1.4	3.51	970	276.07 ± 52.98	3.7	72.07
Promoter	Rise (3)	826	77.09 ± 21.1	-0.0	77.64	1034	432.02 ± 31.33	1.4	373.66
Promoter	Bend (4)	935	-225.33 ± 64.46	-0.1	-217.91	1019	-984.66 ± 77.42	0.0	-987.58
Promoter	Tip (5)	854	-363.88 ± 109.47	-0.1	-356.43	1005	-1175.36 ± 119.24	2.1	-1538.61
Promoter	Inclination (6)	981	-109.63 ± 64.26	-0.1	-103.47	514	-132.21 ± 103.57	-0.1	-115.95
Promoter	Major Groove Width (7)	868	397.78 ± 106.52	0.0	396.18	1022	1978.11 ± 141.6	0.6	1867.73
Promoter	Major Groove Depth (8)	849	-93.18 ± 26.18	-0.0	-92.44	1035	-443.99 ± 35.67	-0.2	-432.3
Promoter	Major Groove Size (9)	101	0.28 ± 0.26	1.1	-0.0	65	0.39 ± 0.33	1.2	0.0
Promoter	Major Groove Distance (10)	1071	-1.7 ± 0.65	-1.6	-0.65	632	-6.46 ± 0.69	-3.5	-3.84
Promoter	Minor Groove Width (11)	865	-125.91 ± 36.4	-0.1	-123.03	1022	-584.52 ± 47.7	-0.2	-573.1
Promoter	Minor Groove Depth (12)	250	-2.37 ± 2.02	-1.1	-0.17	588	13.87 ± 3.63	1.3	9.01
Promoter	Minor Groove Size (13)	889	155.51 ± 40.12	0.0	155.4	983	722.69 ± 47.45	0.0	722.44
Promoter	Minor Groove Distance (14)	875	-29.6 ± 16.32	-1.7	-1.61	502	-83.94 ± 13.83	-4.8	-6.67
Promoter	Persistance Length (15)	931	12600.88 ± 3238.63	0.1	12231.35	1008	59666.19 ± 4100.75	0.4	57339.47
Promoter	Melting Temperature (16)	904	6777.44 ± 1734.3	-0.1	6930.24	979	31218.53 ± 1912.16	-0.3	32179.17
Promoter	Probability contacting nucleosome core (17)	614	-624.17 ± 220.51	0.5	-771.83	928	-4648.98 ± 227.42	-2.1	-3897.63
Promoter	Mobility to bend towards major groove (18)	703	-17.28 ± 4.95	-0.1	-16.86	744	-86.39 ± 6.46	0.1	-87.67
Promoter	Mobility to bend towards minor groove (19)	846	30.33 ± 8.22	-0.0	30.62	1009	143.14 ± 10.42	-0.2	146.16
Promoter	Propeller Twist (20)	821	677.16 ± 172.75	0.5	570.31	711	1854.39 ± 221.15	-3.1	2716.19
Promoter	Clash Strength (21)	910	132.08 ± 33.63	0.2	124.08	886	639.75 ± 43.64	1.0	583.2
Promoter	Enthalpy (22)	1000	-566.53 ± 154.27	0.0	-569.87	1037	-2906.2 ± 164.15	-0.4	-2800.16
Promoter	Entropy (23)	1018	-1231.28 ± 335.01	0.0	-1236.03	1039	-6407.34 ± 359.96	-0.6	-6096.47
Promoter	Shift (RNA) (24)	826	21.86 ± 5.96	-	25.46	948	87.58 ± 5.21	-2.7	111.49

				0.4					
Promoter	Roll (DNA-protein complex) (25)	787	350.13 ± 100.65	0.2	321.39	883	1155.37 ± 146.94	-1.1	1356.18
Promoter	Twist (DNA-protein complex) (26)	698	-186.33 ± 58.07	-0.0	-183.13	979	-634.68 ± 74.02	1.6	-772.39
Promoter	Tilt (DNA-protein complex) (27)	312	-58.58 ± 23.3	-0.2	-53.43	458	-241.98 ± 25.48	1.4	-295.43
Promoter	Slide (DNA-protein complex) (28)	1004	15.9 ± 6.34	-0.4	19.64	1051	106.22 ± 5.74	0.4	101.77
Promoter	Hydrophilicity (RNA) (29)	918	-21.91 ± 12.69	-0.0	-21.75	429	45.8 ± 18.35	0.0	44.6
Promoter	Shift (DNA-protein complex) (30)	896	-15.72 ± 3.76	-2.0	-7.35	575	-28.69 ± 8.57	1.0	-37.26
Promoter	Hydrophilicity (RNA) (31)	786	-26.58 ± 17.87	0.0	-26.83	429	102.97 ± 21.86	0.0	102.16
Promoter	Rise (DNA-protein complex) (32)	818	13.43 ± 3.59	0.1	12.97	821	51.32 ± 4.39	-1.1	57.29
Promoter	Stacking energy (33)	873	-742.28 ± 193.85	0.1	-781.94	981	-3366.49 ± 202.15	0.8	-3597.08
Promoter	Free energy (34)	898	-159.33 ± 40.58	0.0	-161.39	958	-739.18 ± 45.99	0.2	-750.1
Promoter	Free energy (35)	984	-212.57 ± 53.02	0.1	-221.57	1031	-1188.27 ± 69.39	-1.0	-1084.08
Promoter	Free energy (36)	907	-142.09 ± 36.0	0.1	-146.29	989	-675.27 ± 40.86	0.1	-683.24
Promoter	Twist (DNA-protein complex) (37)	724	-90.26 ± 27.61	-1.2	-52.91	790	-426.61 ± 46.84	-2.1	-316.07
Promoter	Free energy (38)	903	-168.97 ± 43.26	0.1	-172.67	980	-777.49 ± 47.76	0.3	-801.61
Promoter	Twist_twist (39)	303	-0.44 ± 0.15	0.4	-0.53	623	-1.27 ± 1.13	-0.8	-0.3
Promoter	Tilt_tilt (40)	1050	0.36 ± 0.11	0.6	0.28	995	1.87 ± 0.17	2.6	1.36
Promoter	Roll_roll (41)	1029	0.17 ± 0.05	0.8	0.12	991	0.53 ± 0.07	-0.5	0.57
Promoter	Twist_tilt (42)	646	-0.5 ± 0.15	-0.2	-0.46	769	-1.74 ± 0.21	1.0	-1.99
Promoter	Twist_roll (43)	896	-0.55 ± 0.14	-0.0	-0.55	968	-2.53 ± 0.17	0.1	-2.54
Promoter	Tilt_roll (44)	747	-0.41 ± 0.12	-0.3	-0.36	800	-1.8 ± 0.21	-0.6	-1.65
Promoter	Shift_shift (45)	1163	24.89 ± 6.77	2.6	6.57	612	103.5 ± 9.97	3.9	57.96
Promoter	Slide_slide (46)	370	-15.41 ± 7.4	-0.2	-13.57	92	-21.35 ± 20.41	-0.7	-5.57
Promoter	Rise_rise (47)	1122	-87.8 ± 19.87	-1.9	-44.81	569	-367.48 ± 55.46	-2.0	-248.63
Promoter	Shift_slide (48)	218	-10.15 ± 4.83	-0.5	-7.44	202	-29.04 ± 28.21	-0.8	-5.49
Promoter	Shift_rise (49)	886	7.59 ± 3.18	1.6	2.42	794	52.59 ± 4.15	5.2	25.24
Promoter	Slide_rise (50)	465	-14.64 ± 9.42	-1.8	2.99	550	-45.88 ± 37.3	-0.4	-25.62
Promoter	Twist_shift (51)	425	-3.1 ± 1.16	-1.0	-1.82	540	-4.88 ± 2.24	-0.8	-2.95
Promoter	Twist_slide (52)	1144	-5.44 ± 2.0	-1.4	-2.39	593	-27.34 ± 2.05	-5.5	-14.13
Promoter	Twist_rise (53)	1101	-2.09 ± 0.67	-1.8	-0.79	329	4.09 ± 4.09	0.9	0.29
Promoter	Tilt_shift (54)	953	-10.69 ± 2.71	-0.0	-10.52	997	-53.76 ± 3.33	-0.8	-49.99
Promoter	Tilt_slide (55)	850	1.02 ± 0.36	0.7	0.74	844	5.06 ± 0.49	2.0	3.91
Promoter	Tilt_rise (56)	550	10.94 ± 3.58	0.6	8.33	590	23.86 ± 4.8	-2.7	38.54
Promoter	Roll_shift (57)	777	-1.64 ± 0.44	-	-1.03	605	-5.84 ± 1.1	-0.2	-5.6

				1.2					
Promoter	Roll_slide (58)	883	3.32 ± 0.89	0.1	3.2	857	10.64 ± 0.98	-2.6	13.95
Promoter	Roll_rise (59)	726	5.64 ± 1.51	0.1	5.5	710	29.88 ± 1.76	0.3	29.17
Promoter	Stacking energy (60)	806	222.07 ± 78.9	0.6	165.39	900	1251.22 ± 107.58	2.9	880.2
Promoter	Twist (61)	1187	-235.3 ± 77.07	-1.8	-93.09	490	-958.9 ± 136.26	-3.0	-526.96
Promoter	Tilt (62)	893	46.44 ± 14.94	0.1	44.55	1001	197.24 ± 19.61	-0.3	204.94
Promoter	Roll (63)	1088	353.47 ± 130.85	1.0	206.77	795	1533.66 ± 142.76	2.1	1179.57
Promoter	Shift (64)	296	4.6 ± 3.16	0.5	3.12	769	9.45 ± 5.73	0.4	6.9
Promoter	Slide (65)	770	96.31 ± 27.39	0.1	90.93	845	268.24 ± 39.61	-2.4	391.95
Promoter	Rise (66)	769	13.61 ± 3.74	-0.0	13.62	993	76.55 ± 5.52	1.3	66.98
Promoter	Slide stiffness (67)	1009	-82.75 ± 21.18	-0.8	-61.17	708	-350.01 ± 40.51	-1.1	-297.64
Promoter	Shift stiffness (68)	819	15.66 ± 9.17	1.7	0.38	241	30.47 ± 29.19	0.5	14.85
Promoter	Roll stiffness (69)	937	0.07 ± 0.02	0.2	0.07	616	0.24 ± 0.03	-2.4	0.35
Promoter	Tilt stiffness (70)	1059	0.76 ± 0.22	0.5	0.63	998	3.6 ± 0.3	1.8	2.94
Promoter	Twist stiffness (71)	565	-0.22 ± 0.11	0.5	-0.29	978	-1.89 ± 0.15	-1.0	-1.7
Promoter	Free energy (72)	927	-187.1 ± 47.26	0.1	-192.66	1000	-916.79 ± 53.54	-0.1	-907.83
Promoter	Free energy (73)	916	-173.11 ± 44.06	0.1	-176.68	995	-819.65 ± 50.11	0.1	-828.06
Promoter	Free energy (74)	893	-151.24 ± 39.07	0.1	-156.54	1003	-703.81 ± 44.35	0.4	-729.47
Promoter	Free energy (75)	896	-159.02 ± 41.28	0.0	-159.16	999	-726.27 ± 48.87	0.2	-739.93
Promoter	GC content (76)	887	310.99 ± 80.25	0.0	310.81	981	1445.14 ± 94.89	0.0	1444.87
Promoter	Purine (AG) content (77)	963	113.71 ± 98.36	-0.0	113.76	349	231.81 ± 208.39	-0.0	231.84
Promoter	Keto (GT) content (78)	1158	-143.5 ± 71.43	0.0	-145.38	1026	-167.9 ± 107.19	-0.0	-165.38
Promoter	Adenine content (79)	815	139.91 ± 110.36	0.0	139.8	529	-774.35 ± 80.38	-0.0	-774.29
Promoter	Guanine content (80)	784	167.69 ± 44.67	-0.0	167.69	721	661.08 ± 70.63	-0.0	661.13
Promoter	Cytosine content (81)	977	204.4 ± 68.0	0.0	204.34	905	795.88 ± 55.22	-0.0	795.96
Promoter	Thymine content (82)	1360	-219.51 ± 80.26	0.0	-219.58	989	-749.61 ± 97.24	-0.0	-749.57
Promoter	Tilt (DNA-protein complex) (83)	562	80.01 ± 24.13	0.0	79.67	860	380.94 ± 39.88	-0.8	423.59
Promoter	Roll (DNA-protein complex) (84)	792	314.05 ± 86.19	0.1	298.49	891	1273.16 ± 109.26	-0.5	1337.14
Promoter	Shift (DNA-protein complex) (85)	955	12.35 ± 2.87	2.1	5.51	575	18.95 ± 14.25	1.0	4.47
Promoter	Slide (DNA-protein complex) (86)	546	10.61 ± 4.64	-0.6	13.83	897	28.73 ± 5.81	-3.9	58.45
Promoter	Rise (DNA-protein complex) (87)	836	13.39 ± 3.54	-0.0	13.64	915	69.7 ± 4.55	0.9	63.8
Promoter	Twist (88)	1000	-371.73 ± 98.53	-0.4	-313.64	835	-1788.97 ± 160.42	-1.1	-1554.1
Promoter	Tilt (89)	992	-133.45 ± 41.71	-1.4	-68.7	565	-364.2 ± 73.54	-0.9	-296.46
Promoter	Roll (90)	1007	388.34 ±	0.4	335.4	1018	2183.81 ±	2.6	1683.48

			105.99				151.97		
Promoter	Slide (91)	822	82.89 ± 22.55	0.3	75.34	784	271.19 ± 29.18	-1.8	334.51
Promoter	Twist (92)	1119	-143.97 ± 38.86	-2.4	-45.53	582	-512.85 ± 66.33	-3.0	-298.82
Promoter	Tilt (93)	964	230.47 ± 70.26	0.9	157.9	807	1037.11 ± 110.35	1.8	805.12
Promoter	Roll (94)	1007	342.75 ± 102.87	1.2	206.01	801	1562.02 ± 138.02	2.6	1135.36
Promoter	Shift (95)	681	-11.44 ± 4.57	-2.3	-1.02	369	-23.33 ± 7.34	-2.6	-3.56
Promoter	Slide (96)	817	103.51 ± 28.09	0.2	96.66	848	382.67 ± 36.43	-1.2	436.1
Promoter	Rise (97)	882	13.34 ± 3.4	-0.1	13.8	847	71.51 ± 4.32	0.7	67.21
Promoter	Twist (98)	686	-185.68 ± 74.52	-1.2	-92.29	562	-426.46 ± 100.03	-0.4	-385.12
Promoter	Wedge (99)	119	-132.04 ± 119.76	-1.1	2.12	388	-475.52 ± 436.03	-1.0	-54.4
Promoter	Direction (100)	885	-10688.34 ± 7596.46	-0.2	-8238.82	873	29695.81 ± 2782.96	0.3	28551.86
Promoter	Slide (RNA) (101)	862	-55.43 ± 15.26	0.1	-58.03	1038	-289.23 ± 19.46	-0.7	-270.19
Promoter	Rise (RNA) (102)	775	12.74 ± 5.67	0.4	10.45	972	71.9 ± 5.2	2.6	54.71
Promoter	Tilt (RNA) (103)	362	82.68 ± 37.77	0.5	63.91	857	145.85 ± 62.51	0.3	125.85
Promoter	Roll (RNA) (104)	674	244.45 ± 73.99	-0.2	260.3	800	1485.34 ± 101.79	2.1	1197.04
Promoter	Twist (RNA) (105)	966	-76.22 ± 51.5	-0.6	-42.35	117	-213.59 ± 26.58	-0.1	-211.72
Promoter	Stacking energy (RNA) (106)	1040	214.13 ± 59.27	0.6	168.69	1013	869.68 ± 92.36	1.0	758.45
Promoter	Rise stiffness (107)	592	37.23 ± 13.96	-0.5	45.99	1030	238.55 ± 28.47	0.6	219.83
Promoter	Melting Temperature (108)	905	19.54 ± 5.0	-0.1	19.98	978	90.09 ± 5.51	-0.3	92.8
Promoter	Stacking energy (109)	864	74.01 ± 19.32	-0.2	79.51	986	342.48 ± 19.74	-0.8	366.07
Promoter	Enthalpy (RNA) (110)	910	-825.41 ± 215.29	-0.1	-807.89	983	-3527.22 ± 327.05	0.1	-3583.6
Promoter	Entropy (RNA) (111)	918	-1684.93 ± 442.55	-0.1	-1644.72	994	-6980.53 ± 660.61	0.2	-7156.18
Promoter	Free energy (RNA) (112)	890	-295.28 ± 76.4	-0.0	-290.86	967	-1330.4 ± 91.6	0.1	-1339.59
Promoter	Free energy (RNA) (113)	875	-322.17 ± 84.2	-0.1	-316.03	961	-1442.75 ± 102.1	0.1	-1457.88
Promoter	Enthalpy (RNA) (114)	834	-870.03 ± 235.26	-0.0	-868.29	985	-3842.8 ± 284.5	0.3	-3970.89
Promoter	Entropy (RNA) (115)	810	-1767.43 ± 488.2	0.0	-1781.01	994	-7741.58 ± 590.52	0.5	-8104.7
Promoter	Roll (116)	730	639.68 ± 183.75	0.1	624.18	790	2230.94 ± 234.86	-1.5	2667.13
Promoter	Tilt (117)	1043	-191.71 ± 148.53	-0.2	-150.63	483	-294.97 ± 249.8	-0.1	-255.5
Promoter	Twist (118)	852	-348.5 ± 91.08	-0.0	-348.41	921	-1560.08 ± 110.46	0.2	-1595.66
Promoter	Roll (119)	1020	208.2 ± 61.99	0.8	146.21	661	590.51 ± 85.73	-1.7	764.05
Promoter	Twist (120)	879	-349.0 ± 86.67	-0.6	-277.3	716	-1009.63 ± 117.57	2.4	-1359.77

Promoter	Flexibility_slide (121)	782	-945.85 ± 256.74	0.0	-950.94	833	-3639.85 ± 302.13	1.5	-4265.33
Promoter	Flexibility_shift (122)	1043	483.31 ± 117.05	0.6	386.31	667	1577.48 ± 224.44	-0.8	1782.71
Promoter	Enthalpy (123)	906	-236.39 ± 59.66	0.3	-260.21	987	-1147.38 ± 57.0	0.7	-1216.53
Promoter	Entropy (124)	870	-217.02 ± 62.9	0.5	-266.23	834	-1080.53 ± 34.6	2.0	-1266.77
Promoter	Free energy (125)	916	-173.11 ± 44.06	0.1	-176.68	995	-819.65 ± 50.11	0.1	-828.06
CTCF Binding Site	Twist (1)	945	-165.89 ± 52.96	-1.7	-69.79	246	-276.28 ± 49.68	-0.3	-257.42
CTCF Binding Site	Stacking energy (2)	151	26.74 ± 23.57	1.1	1.03	359	167.02 ± 33.12	4.5	17.17
CTCF Binding Site	Rise (3)	825	54.28 ± 17.21	-0.0	54.8	316	195.89 ± 33.98	0.5	173.32
CTCF Binding Site	Bend (4)	899	-158.52 ± 53.31	-0.1	-151.24	305	-492.33 ± 84.32	-0.0	-486.88
CTCF Binding Site	Tip (5)	821	-269.13 ± 85.17	-0.2	-251.02	301	-813.41 ± 136.67	-0.1	-791.81
CTCF Binding Site	Inclination (6)	1125	-74.22 ± 47.86	0.0	-75.36	985	-19.25 ± 3.82	-0.5	-16.62
CTCF Binding Site	Major Groove Width (7)	857	279.46 ± 84.89	-0.0	281.83	313	948.11 ± 162.12	0.3	885.82
CTCF Binding Site	Major Groove Depth (8)	830	-64.47 ± 20.67	0.1	-65.9	312	-225.91 ± 38.83	-0.4	-206.71
CTCF Binding Site	Major Groove Size (9)	75	-0.22 ± 0.21	-1.1	-0.0	318	-0.06 ± 0.05	-1.1	0.0
CTCF Binding Site	Major Groove Distance (10)	1005	-1.21 ± 0.6	-0.9	-0.66	292	-2.88 ± 0.5	-2.0	-1.74
CTCF Binding Site	Minor Groove Width (11)	838	-84.85 ± 27.77	0.1	-88.22	303	-302.66 ± 51.64	-0.4	-275.69
CTCF Binding Site	Minor Groove Depth (12)	350	-1.45 ± 0.94	-2.3	0.85	212	3.72 ± 0.73	-0.2	3.89
CTCF Binding Site	Minor Groove Size (13)	896	110.78 ± 31.58	-0.0	110.83	310	348.04 ± 58.89	0.0	348.0
CTCF Binding Site	Minor Groove Distance (14)	689	-18.69 ± 13.07	-1.2	-2.64	284	-45.96 ± 8.31	-5.5	-0.01
CTCF Binding Site	Persistance Length (15)	917	8814.67 ± 2427.19	0.1	8620.62	314	28612.67 ± 4923.85	0.2	27293.24
CTCF Binding Site	Melting Temperature (16)	897	4800.85 ± 1343.26	-0.1	4949.23	306	14511.12 ± 2454.38	-0.3	15575.52
CTCF Binding Site	Probability contacting nucleosome core (17)	733	-435.79 ± 141.92	0.6	-554.92	292	-1360.8 ± 250.21	1.1	-1803.44
CTCF Binding Site	Mobility to bend towards major groove (18)	745	-13.65 ± 3.93	-0.1	-13.12	271	-43.12 ± 7.25	-0.2	-41.09
CTCF Binding Site	Mobility to bend towards minor groove (19)	826	22.18 ± 6.82	-0.0	22.37	306	70.61 ± 12.05	0.1	69.2
CTCF Binding Site	Propeller Twist (20)	800	547.27 ± 149.45	0.5	452.18	306	1388.96 ± 224.24	-0.1	1424.22
CTCF Binding Site	Clash Strength (21)	902	96.32 ± 26.32	0.3	86.35	309	310.16 ± 51.91	0.5	276.98
CTCF Binding Site	Enthalpy (22)	962	-383.51 ± 105.0	0.2	-416.0	317	-1141.15 ± 204.81	0.6	-1325.83
CTCF Binding Site	Entropy (23)	979	-817.93 ± 227.37	0.2	-900.22	321	-2397.5 ± 439.95	0.7	-2874.37
CTCF Binding Site	Shift (RNA) (24)	822	16.75 ± 4.76	-0.2	18.3	296	40.39 ± 6.93	-1.5	57.06
CTCF Binding Site	Roll (DNA-protein complex) (25)	768	290.11 ± 89.59	0.6	220.36	298	854.73 ± 134.46	0.8	710.02
CTCF Binding Site	Twist (DNA-protein complex)	710	-152.27 ±	-	-128.65	311	-480.4 ± 79.66	-0.8	-397.74

	(26)		49.26	0.4					
CTCF Binding Site	Tilt (DNA-protein complex) (27)	444	-47.51 ± 14.74	-0.3	-41.21	263	-119.65 ± 21.21	0.8	-147.02
CTCF Binding Site	Slide (DNA-protein complex) (28)	1094	9.25 ± 5.59	-0.2	10.59	265	14.97 ± 3.95	-3.4	46.72
CTCF Binding Site	Hydrophilicity (RNA) (29)	1045	-15.62 ± 11.08	0.0	-15.76	368	11.31 ± 2.59	0.3	10.36
CTCF Binding Site	Shift (DNA-protein complex) (30)	846	-11.83 ± 2.85	-1.8	-5.82	300	-30.36 ± 4.42	-2.1	-19.22
CTCF Binding Site	Hydrophilicity (RNA) (31)	812	20.02 ± 12.41	-0.0	20.04	237	37.16 ± 6.79	0.1	36.18
CTCF Binding Site	Rise (DNA-protein complex) (32)	813	10.47 ± 2.95	0.5	8.69	305	31.25 ± 5.0	0.4	28.83
CTCF Binding Site	Stacking energy (33)	864	-532.37 ± 149.74	0.1	-560.43	300	-1569.48 ± 262.66	0.5	-1760.49
CTCF Binding Site	Free energy (34)	896	-113.92 ± 31.71	0.0	-115.1	307	-347.74 ± 58.64	0.2	-362.27
CTCF Binding Site	Free energy (35)	957	-139.18 ± 40.09	0.3	-156.0	312	-452.38 ± 81.26	0.4	-500.69
CTCF Binding Site	Free energy (36)	905	-100.66 ± 27.99	0.1	-103.88	309	-307.92 ± 52.55	0.3	-329.1
CTCF Binding Site	Twist (DNA-protein complex) (37)	754	-71.22 ± 22.2	-0.9	-46.87	315	-280.02 ± 46.67	-2.5	-145.74
CTCF Binding Site	Free energy (38)	897	-119.72 ± 33.51	0.1	-123.31	306	-362.26 ± 61.25	0.3	-388.03
CTCF Binding Site	Twist_twist (39)	396	-0.39 ± 0.12	0.2	-0.42	209	-0.82 ± 0.14	2.1	-1.34
CTCF Binding Site	Tilt_tilt (40)	1002	0.24 ± 0.11	0.3	0.2	330	0.78 ± 0.15	0.9	0.62
CTCF Binding Site	Roll_roll (41)	989	0.12 ± 0.03	0.7	0.08	329	0.41 ± 0.07	1.7	0.27
CTCF Binding Site	Twist_tilt (42)	666	-0.42 ± 0.13	-0.6	-0.33	296	-1.39 ± 0.21	-1.4	-1.02
CTCF Binding Site	Twist_roll (43)	892	-0.4 ± 0.11	-0.1	-0.39	312	-1.26 ± 0.21	-0.1	-1.22
CTCF Binding Site	Tilt_roll (44)	748	-0.33 ± 0.1	-0.7	-0.24	296	-1.11 ± 0.17	-1.4	-0.81
CTCF Binding Site	Shift_shift (45)	1124	14.32 ± 5.46	1.9	3.76	363	40.57 ± 8.0	2.2	19.68
CTCF Binding Site	Slide_slide (46)	455	-14.63 ± 6.11	-0.5	-10.98	98	-6.78 ± 1.13	5.8	-34.92
CTCF Binding Site	Rise_rise (47)	1102	-56.29 ± 16.86	-1.1	-34.64	265	-120.18 ± 20.22	-0.3	-111.64
CTCF Binding Site	Shift_slide (48)	338	-8.85 ± 3.92	-0.6	-6.31	222	-15.63 ± 2.97	0.5	-17.48
CTCF Binding Site	Shift_rise (49)	382	3.83 ± 3.43	0.7	1.26	439	8.28 ± 2.5	1.1	4.92
CTCF Binding Site	Slide_rise (50)	272	-9.69 ± 7.57	-1.6	2.49	275	-33.15 ± 5.57	-7.5	15.2
CTCF Binding Site	Twist_shift (51)	470	-2.87 ± 0.9	-1.4	-1.51	263	-8.15 ± 1.1	-2.6	-4.88
CTCF Binding Site	Twist_slide (52)	1040	-3.3 ± 2.1	-1.0	-1.24	307	-6.19 ± 1.35	-0.3	-5.74
CTCF Binding Site	Twist_rise (53)	1086	-1.49 ± 0.43	-2.0	-0.55	183	-0.62 ± 0.2	4.3	-2.08
CTCF Binding Site	Tilt_shift (54)	932	-7.43 ± 2.06	-0.0	-7.36	311	-23.49 ± 4.05	0.0	-23.61
CTCF Binding Site	Tilt_slide (55)	879	0.78 ± 0.28	0.7	0.56	304	2.37 ± 0.39	1.0	1.86
CTCF Binding Site	Tilt_rise (56)	588	9.42 ± 2.72	0.7	6.99	282	25.58 ± 3.72	0.8	21.48
CTCF Binding Site	Roll_shift (57)	755	-1.35 ± 0.38	-1.0	-0.9	294	-4.79 ± 0.72	-2.4	-2.76
CTCF Binding Site	Roll_slide (58)	858	2.58 ± 0.72	0.4	2.24	311	7.23 ± 1.18	0.1	7.1
CTCF Binding Site	Roll_rise (59)	759	4.36 ± 1.2	-0.0	4.44	291	12.58 ± 2.1	-0.5	14.14

CTCF Binding Site	Stacking energy (60)	820	164.9 ± 60.97	0.6	121.96	298	581.72 ± 96.47	1.5	400.5
CTCF Binding Site	Twist (61)	1161	-151.44 ± 77.86	-0.9	-74.48	144	-153.2 ± 35.85	1.5	-233.69
CTCF Binding Site	Tilt (62)	861	29.74 ± 10.33	-0.1	31.58	297	103.68 ± 17.92	0.1	100.24
CTCF Binding Site	Roll (63)	1059	274.44 ± 122.2	0.7	183.76	307	580.58 ± 103.43	0.0	575.85
CTCF Binding Site	Shift (64)	362	4.0 ± 2.67	0.5	2.72	284	9.97 ± 2.07	0.9	7.98
CTCF Binding Site	Slide (65)	740	79.94 ± 22.89	0.6	63.72	304	239.38 ± 37.73	0.8	202.15
CTCF Binding Site	Rise (66)	772	9.52 ± 2.93	-0.0	9.63	305	35.76 ± 6.01	0.6	30.75
CTCF Binding Site	Slide stiffness (67)	946	-60.41 ± 16.4	-0.8	-45.01	306	-186.52 ± 30.58	-1.1	-141.96
CTCF Binding Site	Shift stiffness (68)	589	-11.98 ± 11.72	-1.0	0.15	282	3.47 ± 1.87	1.7	0.29
CTCF Binding Site	Roll stiffness (69)	882	0.06 ± 0.01	0.3	0.05	265	0.13 ± 0.02	-1.4	0.17
CTCF Binding Site	Tilt stiffness (70)	1005	0.53 ± 0.19	0.3	0.45	323	1.65 ± 0.29	0.7	1.39
CTCF Binding Site	Twist stiffness (71)	674	-0.13 ± 0.05	0.9	-0.21	302	-0.4 ± 0.09	2.0	-0.75
CTCF Binding Site	Free energy (72)	920	-129.63 ± 34.72	0.1	-136.59	310	-397.3 ± 68.4	0.4	-433.66
CTCF Binding Site	Free energy (73)	909	-121.45 ± 34.11	0.1	-125.77	309	-372.89 ± 63.74	0.3	-397.24
CTCF Binding Site	Free energy (74)	884	-107.95 ± 30.59	0.1	-111.43	307	-329.04 ± 56.13	0.3	-351.8
CTCF Binding Site	Free energy (75)	892	-113.11 ± 32.46	0.0	-113.45	310	-355.25 ± 60.33	0.0	-356.51
CTCF Binding Site	GC content (76)	896	221.6 ± 63.19	-0.0	221.66	309	696.1 ± 117.77	0.0	695.99
CTCF Binding Site	Purine (AG) content (77)	1046	79.02 ± 60.63	-0.0	79.02	490	-18.5 ± 8.17	0.0	-18.5
CTCF Binding Site	Keto (GT) content (78)	1155	-95.63 ± 49.37	0.0	-96.85	1251	-57.88 ± 6.9	-3.5	-28.79
CTCF Binding Site	Adenine content (79)	752	-116.46 ± 44.93	-0.0	-116.43	216	-337.34 ± 59.31	-0.0	-337.29
CTCF Binding Site	Guanine content (80)	821	117.37 ± 38.38	0.0	117.33	206	347.97 ± 59.39	0.0	347.94
CTCF Binding Site	Cytosine content (81)	932	143.66 ± 52.09	0.0	143.64	440	348.13 ± 58.63	0.0	348.06
CTCF Binding Site	Thymine content (82)	1251	-178.02 ± 69.45	0.0	-178.08	487	-358.75 ± 58.85	-0.0	-358.71
CTCF Binding Site	Tilt (DNA-protein complex) (83)	593	64.61 ± 21.39	0.1	62.26	329	187.0 ± 35.73	-0.3	202.52
CTCF Binding Site	Roll (DNA-protein complex) (84)	791	240.9 ± 70.18	0.3	214.72	306	780.98 ± 125.96	0.7	662.37
CTCF Binding Site	Shift (DNA-protein complex) (85)	899	9.05 ± 2.21	2.0	3.98	348	21.42 ± 3.41	1.8	14.1
CTCF Binding Site	Slide (DNA-protein complex) (86)	591	7.82 ± 3.48	-0.4	9.79	221	17.46 ± 3.25	-2.4	31.5
CTCF Binding Site	Rise (DNA-protein complex) (87)	840	9.9 ± 2.87	0.1	9.66	311	32.17 ± 5.46	0.2	30.41
CTCF Binding Site	Twist (88)	956	-258.91 ± 71.03	-0.3	-228.17	309	-798.87 ± 135.43	-0.4	-731.0
CTCF Binding Site	Tilt (89)	935	-109.67 ± 37.4	-1.4	-51.45	283	-256.73 ± 38.41	-2.3	-154.61
CTCF Binding Site	Roll (90)	995	269.96 ± 94.75	0.3	237.46	333	846.73 ± 159.3	0.4	771.53
CTCF Binding Site	Slide (91)	800	67.46 ± 18.78	0.6	53.64	308	209.81 ± 33.59	1.0	166.84

CTCF Binding Site	Twist (92)	1061	-98.26 ± 26.92	-2.3	-33.62	333	-305.72 ± 53.33	-3.1	-122.01
CTCF Binding Site	Tilt (93)	929	173.71 ± 55.74	0.8	118.41	304	549.42 ± 90.64	1.5	381.03
CTCF Binding Site	Roll (94)	978	247.9 ± 76.35	1.0	161.82	327	802.28 ± 140.75	1.7	519.97
CTCF Binding Site	Shift (95)	702	-9.26 ± 4.38	-1.8	-1.22	176	-20.03 ± 2.66	-6.8	-1.83
CTCF Binding Site	Slide (96)	789	82.7 ± 23.41	0.5	68.58	309	263.43 ± 42.98	0.9	214.26
CTCF Binding Site	Rise (97)	861	9.83 ± 2.66	0.0	9.64	307	31.69 ± 5.45	0.1	31.16
CTCF Binding Site	Twist (98)	723	-164.41 ± 66.64	-1.3	-71.59	271	-368.18 ± 52.88	-2.4	-220.17
CTCF Binding Site	Wedge (99)	300	-75.29 ± 40.14	-0.8	-41.48	368	-229.55 ± 53.64	-1.3	-148.59
CTCF Binding Site	Direction (100)	980	7281.08 ± 4334.56	0.7	4100.5	116	12301.41 ± 2172.75	-0.4	13540.56
CTCF Binding Site	Slide (RNA) (101)	865	-39.73 ± 12.18	0.0	-40.46	309	-125.8 ± 21.9	0.1	-129.55
CTCF Binding Site	Rise (RNA) (102)	839	10.18 ± 4.82	0.4	7.95	287	26.62 ± 4.59	0.1	26.11
CTCF Binding Site	Tilt (RNA) (103)	430	73.34 ± 28.67	0.5	57.98	284	219.44 ± 34.95	1.1	171.82
CTCF Binding Site	Roll (RNA) (104)	719	166.45 ± 62.23	-0.2	179.87	263	620.34 ± 110.61	0.3	576.05
CTCF Binding Site	Twist (RNA) (105)	897	-52.22 ± 41.43	-0.3	-39.05	180	28.46 ± 6.57	7.8	-114.28
CTCF Binding Site	Stacking energy (RNA) (106)	1001	148.11 ± 49.38	0.6	112.48	333	504.66 ± 91.56	1.3	363.06
CTCF Binding Site	Rise stiffness (107)	639	23.11 ± 11.45	-0.6	32.69	306	88.97 ± 17.32	-0.6	102.97
CTCF Binding Site	Melting Temperature (108)	898	13.84 ± 3.87	-0.1	14.27	306	41.83 ± 7.08	-0.3	44.91
CTCF Binding Site	Stacking energy (109)	849	53.26 ± 14.94	-0.2	56.94	297	154.66 ± 25.96	-0.6	179.21
CTCF Binding Site	Enthalpy (RNA) (110)	881	-614.39 ± 177.46	-0.1	-580.94	319	-1823.61 ± 305.19	-0.2	-1744.8
CTCF Binding Site	Entropy (RNA) (111)	884	-1312.42 ± 395.9	-0.3	-1177.1	325	-3665.28 ± 611.28	-0.2	-3495.18
CTCF Binding Site	Free energy (RNA) (112)	885	-212.07 ± 60.21	-0.1	-206.9	309	-672.46 ± 112.86	-0.2	-648.68
CTCF Binding Site	Free energy (RNA) (113)	868	-233.87 ± 67.13	-0.1	-225.65	309	-744.3 ± 124.6	-0.2	-705.71
CTCF Binding Site	Enthalpy (RNA) (114)	827	-639.27 ± 188.73	-0.1	-621.72	307	-2047.04 ± 340.99	-0.2	-1937.65
CTCF Binding Site	Entropy (RNA) (115)	801	-1307.85 ± 392.73	-0.1	-1277.26	305	-4204.29 ± 698.58	-0.2	-3972.82
CTCF Binding Site	Roll (116)	739	532.49 ± 154.03	0.5	439.03	298	1601.99 ± 252.45	0.7	1376.4
CTCF Binding Site	Tilt (117)	1174	-145.24 ± 114.15	-0.3	-109.13	909	-30.72 ± 8.78	0.2	-33.35
CTCF Binding Site	Twist (118)	847	-260.78 ± 74.0	-0.1	-248.07	309	-807.06 ± 135.32	-0.2	-776.54
CTCF Binding Site	Roll (119)	983	166.58 ± 52.39	0.6	129.22	276	311.01 ± 47.41	-1.2	405.19
CTCF Binding Site	Twist (120)	864	-279.26 ± 74.64	-0.6	-219.5	307	-760.09 ± 122.21	-0.4	-695.55
CTCF Binding Site	Flexibility_slide (121)	763	-754.15 ± 210.61	-0.3	-679.32	302	-2275.41 ± 366.92	-0.3	-2123.93
CTCF Binding Site	Flexibility_shift (122)	1015	388.45 ± 132.43	0.6	292.3	286	687.24 ± 111.03	-1.3	932.89

CTCF Binding Site	Enthalpy (123)	899	-165.46 ± 44.75	0.3	-185.66	296	-461.0 ± 78.93	1.0	-589.73
CTCF Binding Site	Entropy (124)	856	-152.89 ± 43.98	0.5	-187.87	226	-288.35 ± 51.09	2.9	-625.8
CTCF Binding Site	Free energy (125)	909	-121.45 ± 34.11	0.1	-125.77	309	-372.89 ± 63.74	0.3	-397.24
Open chromatin	Twist (1)	493	-56.62 ± 28.27	-0.9	-25.75	3	57.22 ± 28.12	1.3	-2.2
Open chromatin	Stacking energy (2)	9	11.93 ± 9.26	1.2	0.85	-	-	-	-
Open chromatin	Rise (3)	249	21.45 ± 13.87	-0.0	21.75	-	-	-	-
Open chromatin	Bend (4)	400	-69.3 ± 38.02	-0.1	-66.12	-	-	-	-
Open chromatin	Tip (5)	348	-123.99 ± 72.38	-0.1	-110.38	-	-	-	-
Open chromatin	Inclination (6)	1268	-32.57 ± 5.26	-0.4	-29.31	805	-23.54 ± 4.85	-0.6	-19.73
Open chromatin	Major Groove Width (7)	317	115.3 ± 73.81	-0.0	116.6	-	-	-	-
Open chromatin	Major Groove Depth (8)	275	-27.53 ± 17.15	-0.0	-27.3	-	-	-	-
Open chromatin	Major Groove Size (9)	320	0.09 ± 0.04	1.9	-0.0	321	0.08 ± 0.03	3.1	0.0
Open chromatin	Major Groove Distance (10)	638	-0.52 ± 0.2	-1.2	-0.22	-	-	-	-
Open chromatin	Minor Groove Width (11)	276	-38.18 ± 22.62	-0.0	-36.67	-	-	-	-
Open chromatin	Minor Groove Depth (12)	294	-0.63 ± 0.28	-2.2	0.34	2	-0.78 ± 0.76	-0.7	-0.09
Open chromatin	Minor Groove Size (13)	394	46.29 ± 29.11	0.0	46.28	-	-	-	-
Open chromatin	Minor Groove Distance (14)	277	-8.33 ± 3.84	-2.2	0.0	-	-	-	-
Open chromatin	Persistance Length (15)	418	3655.07 ± 2259.85	0.0	3504.25	-	-	-	-
Open chromatin	Melting Temperature (16)	407	2019.33 ± 1242.19	-0.0	2076.55	-	-	-	-
Open chromatin	Probability contacting nucleosome core (17)	127	-151.58 ± 111.47	0.3	-201.64	-	-	-	-
Open chromatin	Mobility to bend towards major groove (18)	150	-5.38 ± 3.74	-0.0	-5.14	-	-	-	-
Open chromatin	Mobility to bend towards minor groove (19)	289	9.18 ± 5.8	0.0	9.07	-	-	-	-
Open chromatin	Propeller Twist (20)	365	231.74 ± 153.63	0.2	185.65	-	-	-	-
Open chromatin	Clash Strength (21)	390	40.13 ± 24.33	0.1	35.6	-	-	-	-
Open chromatin	Enthalpy (22)	497	-150.48 ± 90.91	0.2	-173.38	-	-	-	-
Open chromatin	Entropy (23)	533	-312.91 ± 182.22	0.1	-348.06	-	-	-	-
Open chromatin	Shift (RNA) (24)	396	6.82 ± 4.3	-0.2	7.88	-	-	-	-
Open chromatin	Roll (DNA-protein complex) (25)	299	126.6 ± 78.14	0.3	100.48	-	-	-	-
Open chromatin	Twist (DNA-protein complex) (26)	236	-64.24 ± 43.26	-0.1	-56.02	-	-	-	-
Open chromatin	Tilt (DNA-protein complex) (27)	53	-19.83 ± 14.82	-0.2	-16.05	-	-	-	-
Open chromatin	Slide (DNA-protein complex) (28)	694	2.81 ± 1.05	-0.5	4.9	7	-6.68 ± 3.28	-0.9	-0.41
Open chromatin	Hydrophilicity (RNA) (29)	870	-5.8 ± 2.2	-	-5.61	184	-5.77 ± 2.23	-0.2	-5.16

				0.1					
Open chromatin	Shift (DNA-protein complex) (30)	396	-5.27 ± 3.38	-0.7	-2.48	-	-	-	-
Open chromatin	Hydrophilicity (RNA) (31)	292	-6.84 ± 4.03	0.0	-6.86	1	-7.12 ± 5.76	-0.0	-6.87
Open chromatin	Rise (DNA-protein complex) (32)	351	4.52 ± 2.91	0.1	3.97	-	-	-	-
Open chromatin	Stacking energy (33)	376	-225.95 ± 138.19	0.1	-236.63	-	-	-	-
Open chromatin	Free energy (34)	402	-47.45 ± 29.79	0.0	-48.2	-	-	-	-
Open chromatin	Free energy (35)	449	-54.25 ± 33.59	0.2	-64.04	-	-	-	-
Open chromatin	Free energy (36)	407	-41.54 ± 26.15	0.1	-43.59	-	-	-	-
Open chromatin	Twist (DNA-protein complex) (37)	183	-30.79 ± 20.39	-0.6	-17.6	-	-	-	-
Open chromatin	Free energy (38)	405	-50.37 ± 31.02	0.0	-51.74	-	-	-	-
Open chromatin	Twist_twist (39)	47	-0.16 ± 0.14	0.0	-0.16	2	-0.22 ± 0.13	-0.8	-0.03
Open chromatin	Tilt_tilt (40)	552	0.11 ± 0.05	0.5	0.08	-	-	-	-
Open chromatin	Roll_roll (41)	495	0.05 ± 0.03	0.5	0.03	-	-	-	-
Open chromatin	Twist_tilt (42)	160	-0.18 ± 0.13	-0.3	-0.14	-	-	-	-
Open chromatin	Twist_roll (43)	393	-0.16 ± 0.11	-0.0	-0.16	-	-	-	-
Open chromatin	Tilt_roll (44)	186	-0.14 ± 0.09	-0.3	-0.11	-	-	-	-
Open chromatin	Shift_shift (45)	764	6.59 ± 2.18	2.1	1.53	2	-6.98 ± 5.78	-1.1	-0.32
Open chromatin	Slide_slide (46)	64	-5.23 ± 3.35	0.0	-5.39	65	-8.38 ± 1.16	-1.3	-1.22
Open chromatin	Rise_rise (47)	711	-20.42 ± 9.3	-0.5	-13.69	-	-	-	-
Open chromatin	Shift_slide (48)	99	-3.92 ± 2.92	-0.3	-2.71	3	-6.71 ± 2.86	-1.4	-0.83
Open chromatin	Shift_rise (49)	15	1.24 ± 1.13	0.7	0.45	7	-5.81 ± 1.37	-3.5	-0.26
Open chromatin	Slide_rise (50)	89	-5.31 ± 2.81	-2.3	1.6	-	-	-	-
Open chromatin	Twist_shift (51)	70	-1.26 ± 0.92	-0.5	-0.76	3	-2.04 ± 1.07	-1.4	-0.17
Open chromatin	Twist_slide (52)	674	-1.21 ± 0.49	-0.9	-0.66	7	2.12 ± 0.98	1.7	-0.02
Open chromatin	Twist_rise (53)	849	-0.66 ± 0.23	-1.2	-0.26	58	0.46 ± 0.21	1.0	0.05
Open chromatin	Tilt_shift (54)	432	-3.03 ± 1.8	-0.0	-3.01	-	-	-	-
Open chromatin	Tilt_slide (55)	339	0.31 ± 0.17	0.3	0.24	-	-	-	-
Open chromatin	Tilt_rise (56)	144	4.15 ± 3.07	0.3	2.89	2	4.43 ± 3.35	0.8	0.6
Open chromatin	Roll_shift (57)	204	-0.62 ± 0.41	-0.6	-0.35	-	-	-	-
Open chromatin	Roll_slide (58)	411	1.12 ± 0.71	0.1	0.98	-	-	-	-
Open chromatin	Roll_rise (59)	204	1.67 ± 1.15	-0.0	1.74	-	-	-	-
Open chromatin	Stacking energy (60)	221	68.25 ± 36.68	0.4	50.12	-	-	-	-
Open chromatin	Twist (61)	964	-44.53 ± 14.04	-0.7	-28.84	7	74.34 ± 21.07	2.0	-2.66
Open chromatin	Tilt (62)	328	14.56 ± 7.51	0.1	13.56	-	-	-	-
Open chromatin	Roll (63)	716	100.86 ± 42.73	0.4	76.34	-	-	-	-
Open chromatin	Shift (64)	136	1.55 ± 0.96	0.3	1.21	-	-	-	-
Open chromatin	Slide (65)	259	35.3 ± 23.85	0.2	28.04	-	-	-	-
Open chromatin	Rise (66)	157	3.86 ± 2.62	-0.0	3.93	-	-	-	-
Open chromatin	Slide stiffness (67)	501	-26.55 ± 14.6	-	-18.33	-	-	-	-

				0.4					
Open chromatin	Shift stiffness (68)	245	3.98 ± 2.29	1.7	0.18	62	-6.13 ± 1.95	-3.1	-0.06
Open chromatin	Roll stiffness (69)	375	0.03 ± 0.02	0.1	0.02	-	-	-	-
Open chromatin	Tilt stiffness (70)	577	0.24 ± 0.11	0.4	0.18	-	-	-	-
Open chromatin	Twist stiffness (71)	59	-0.04 ± 0.03	0.5	-0.07	2	0.07 ± 0.06	0.6	-0.0
Open chromatin	Free energy (72)	432	-53.35 ± 32.83	0.1	-57.16	-	-	-	-
Open chromatin	Free energy (73)	413	-50.4 ± 31.24	0.0	-52.54	-	-	-	-
Open chromatin	Free energy (74)	386	-44.54 ± 28.05	0.1	-46.67	-	-	-	-
Open chromatin	Free energy (75)	388	-47.42 ± 29.7	-0.0	-47.4	-	-	-	-
Open chromatin	GC content (76)	393	92.58 ± 58.2	0.0	92.57	-	-	-	-
Open chromatin	Purine (AG) content (77)	1161	33.59 ± 7.07	0.0	33.55	800	25.55 ± 7.13	-0.0	25.55
Open chromatin	Keto (GT) content (78)	1297	-43.47 ± 7.32	-0.2	-41.5	465	-25.12 ± 10.49	-0.1	-23.84
Open chromatin	Adenine content (79)	45	-37.99 ± 30.98	-0.0	-37.97	-	-	-	-
Open chromatin	Guanine content (80)	280	42.71 ± 30.13	0.0	42.7	-	-	-	-
Open chromatin	Cytosine content (81)	507	49.87 ± 28.61	0.0	49.86	-	-	-	-
Open chromatin	Thymine content (82)	934	-62.34 ± 26.87	-0.0	-62.32	-	-	-	-
Open chromatin	Tilt (DNA-protein complex) (83)	145	25.12 ± 16.99	0.1	22.74	-	-	-	-
Open chromatin	Roll (DNA-protein complex) (84)	284	104.27 ± 68.64	0.2	90.39	-	-	-	-
Open chromatin	Shift (DNA-protein complex) (85)	453	4.11 ± 2.64	0.8	1.59	-	-	-	-
Open chromatin	Slide (DNA-protein complex) (86)	179	3.85 ± 1.91	-0.2	4.51	2	3.78 ± 3.43	0.5	0.8
Open chromatin	Rise (DNA-protein complex) (87)	320	3.88 ± 2.57	0.0	3.73	-	-	-	-
Open chromatin	Twist (88)	483	-107.14 ± 60.27	-0.2	-92.85	-	-	-	-
Open chromatin	Tilt (89)	510	-44.26 ± 22.58	-0.8	-21.67	-	-	-	-
Open chromatin	Roll (90)	529	105.03 ± 53.91	0.1	96.66	-	-	-	-
Open chromatin	Slide (91)	307	28.71 ± 20.17	0.2	22.66	-	-	-	-
Open chromatin	Twist (92)	670	-44.35 ± 21.83	-1.3	-14.05	-	-	-	-
Open chromatin	Tilt (93)	432	75.68 ± 39.58	0.5	49.23	-	-	-	-
Open chromatin	Roll (94)	521	107.67 ± 55.71	0.6	65.91	-	-	-	-
Open chromatin	Shift (95)	220	-3.37 ± 1.65	-1.8	-0.33	2	-3.77 ± 2.35	-1.5	-0.09
Open chromatin	Slide (96)	292	34.32 ± 24.27	0.2	28.77	-	-	-	-
Open chromatin	Rise (97)	297	3.7 ± 2.59	0.0	3.69	-	-	-	-
Open chromatin	Twist (98)	266	-58.77 ± 33.93	-0.7	-32.63	-	-	-	-
Open chromatin	Wedge (99)	94	-38.03 ± 20.61	-0.9	-15.27	3	-41.08 ± 33.37	-1.1	3.32
Open chromatin	Direction (100)	353	-2450.19 ± 1349.29	-0.4	-1787.12	1	-2273.95 ± 1871.5	-0.3	-1417.21
Open chromatin	Slide (RNA) (101)	347	-15.62 ± 9.5	0.1	-16.76	-	-	-	-
Open chromatin	Rise (RNA) (102)	309	3.41 ± 1.71	0.0	3.38	-	-	-	-
Open chromatin	Tilt (RNA) (103)	76	32.4 ± 22.24	0.2	26.29	2	46.83 ± 32.81	1.0	5.64
Open chromatin	Roll (RNA) (104)	141	61.14 ± 39.06	-0.1	69.23	-	-	-	-
Open chromatin	Twist (RNA) (105)	544	-11.64 ± 5.92	-0.0	-11.49	612	22.36 ± 3.82	1.2	0.43

Open chromatin	Stacking energy (RNA) (106)	561	70.25 ± 36.13	0.5	47.28	-	-	-	-
Open chromatin	Rise stiffness (107)	120	8.73 ± 5.42	-0.5	13.45	-	-	-	-
Open chromatin	Melting Temperature (108)	408	5.82 ± 3.58	-0.0	5.99	-	-	-	-
Open chromatin	Stacking energy (109)	373	22.32 ± 13.74	-0.1	24.06	-	-	-	-
Open chromatin	Enthalpy (RNA) (110)	458	-247.64 ± 154.87	-0.0	-239.46	-	-	-	-
Open chromatin	Entropy (RNA) (111)	498	-505.59 ± 313.83	-0.0	-486.97	-	-	-	-
Open chromatin	Free energy (RNA) (112)	390	-89.51 ± 56.68	-0.0	-86.75	-	-	-	-
Open chromatin	Free energy (RNA) (113)	368	-98.5 ± 62.86	-0.0	-94.31	-	-	-	-
Open chromatin	Enthalpy (RNA) (114)	317	-271.08 ± 173.3	-0.0	-260.75	-	-	-	-
Open chromatin	Entropy (RNA) (115)	294	-556.72 ± 356.41	-0.0	-536.69	-	-	-	-
Open chromatin	Roll (116)	248	227.1 ± 155.25	0.2	192.92	-	-	-	-
Open chromatin	Tilt (117)	1187	-45.91 ± 9.74	0.4	-51.27	592	-27.17 ± 8.66	0.6	-34.2
Open chromatin	Twist (118)	366	-106.27 ± 70.62	-0.0	-103.83	-	-	-	-
Open chromatin	Roll (119)	588	67.38 ± 34.65	0.2	57.68	-	-	-	-
Open chromatin	Twist (120)	403	-119.1 ± 78.61	-0.3	-89.34	-	-	-	-
Open chromatin	Flexibility_slide (121)	262	-315.1 ± 216.75	-0.1	-287.9	-	-	-	-
Open chromatin	Flexibility_shift (122)	635	139.9 ± 73.95	0.1	131.6	-	-	-	-
Open chromatin	Enthalpy (123)	423	-68.1 ± 41.25	0.2	-78.32	-	-	-	-
Open chromatin	Entropy (124)	386	-57.03 ± 33.6	0.4	-83.61	-	-	-	-
Open chromatin	Free energy (125)	413	-50.4 ± 31.24	0.0	-52.54	-	-	-	-
ESC_TAD_borders	Twist (1)	948	-193.04 ± 27.43	-4.6	-47.63	414	-284.42 ± 60.96	-2.1	-113.92
ESC_TAD_borders	Stacking energy (2)	301	-44.19 ± 15.05	-3.0	1.59	98	82.49 ± 38.47	2.0	5.47
ESC_TAD_borders	Rise (3)	948	41.2 ± 12.04	-0.4	47.47	190	96.47 ± 39.57	0.3	82.86
ESC_TAD_borders	Bend (4)	978	-130.34 ± 32.47	0.2	-139.37	225	-238.94 ± 104.94	0.1	-250.45
ESC_TAD_borders	Tip (5)	997	-212.35 ± 59.12	0.1	-220.28	181	-365.96 ± 174.72	0.2	-412.87
ESC_TAD_borders	Inclination (6)	360	40.13 ± 15.35	0.6	27.98	365	52.15 ± 22.54	0.4	40.33
ESC_TAD_borders	Major Groove Width (7)	982	228.25 ± 58.61	-0.2	246.86	208	467.51 ± 194.54	0.1	448.31
ESC_TAD_borders	Major Groove Depth (8)	966	-49.73 ± 14.23	0.0	-50.34	191	-107.6 ± 47.1	-0.0	-104.81
ESC_TAD_borders	Major Groove Size (9)	348	0.24 ± 0.09	2.6	-0.0	309	0.34 ± 0.14	2.4	-0.0
ESC_TAD_borders	Major Groove Distance (10)	833	-1.2 ± 0.34	-2.2	-0.42	428	-1.58 ± 0.68	-1.0	-0.79
ESC_TAD_borders	Minor Groove Width (11)	955	-67.11 ± 19.15	0.0	-67.54	195	-141.23 ± 63.66	-0.0	-140.29
ESC_TAD_borders	Minor Groove Depth (12)	414	-2.99 ± 0.82	-4.1	0.51	268	2.56 ± 1.26	1.5	0.5
ESC_TAD_borders	Minor Groove Size (13)	1004	97.9 ± 21.78	0.0	97.86	225	176.93 ± 71.58	-0.0	177.06
ESC_TAD_borders	Minor Groove Distance (14)	501	-18.13 ± 6.04	-3.0	0.01	399	-24.85 ± 10.27	-2.4	-0.0
ESC_TAD_borders	Persistance Length (15)	993	7829.6 ±	0.1	7626.34	226	14572.59 ±	0.1	13833.25

			1801.56				5879.11		
ESC_TAD_borders	Melting Temperature (16)	1011	4475.09 ± 1002.17	0.1	4390.46	231	7576.55 ± 3024.22	-0.1	7934.8
ESC_TAD_borders	Probability contacting nucleosome core (17)	885	-362.57 ± 101.93	0.8	-485.48	189	-876.1 ± 329.83	-0.1	-834.24
ESC_TAD_borders	Mobility to bend towards major groove (18)	999	-11.38 ± 2.92	-0.1	-10.95	207	-20.34 ± 8.57	-0.1	-19.73
ESC_TAD_borders	Mobility to bend towards minor groove (19)	996	18.41 ± 4.96	-0.1	19.36	207	34.2 ± 14.48	0.0	34.18
ESC_TAD_borders	Propeller Twist (20)	1028	513.88 ± 97.92	1.0	385.09	202	669.78 ± 283.66	-0.2	743.92
ESC_TAD_borders	Clash Strength (21)	995	82.25 ± 19.76	0.2	77.42	227	154.07 ± 61.94	0.2	140.44
ESC_TAD_borders	Enthalpy (22)	1012	-391.26 ± 80.97	-0.2	-368.27	240	-651.57 ± 245.05	0.0	-668.92
ESC_TAD_borders	Entropy (23)	1012	-857.08 ± 173.04	-0.2	-796.1	242	-1411.74 ± 521.27	0.0	-1447.39
ESC_TAD_borders	Shift (RNA) (24)	1027	15.94 ± 3.09	-0.1	16.54	218	22.57 ± 8.95	-0.5	29.59
ESC_TAD_borders	Roll (DNA-protein complex) (25)	993	215.77 ± 57.56	0.4	183.58	186	361.7 ± 169.89	-0.1	372.99
ESC_TAD_borders	Twist (DNA-protein complex) (26)	966	-116.98 ± 31.69	-0.7	-89.85	132	-201.73 ± 97.24	0.1	-208.43
ESC_TAD_borders	Tilt (DNA-protein complex) (27)	750	-45.35 ± 11.29	-1.0	-30.61	226	-71.3 ± 28.02	-0.0	-69.6
ESC_TAD_borders	Slide (DNA-protein complex) (28)	838	13.97 ± 2.23	0.3	12.73	375	16.17 ± 5.37	-0.6	23.24
ESC_TAD_borders	Hydrophilicity (RNA) (29)	409	11.09 ± 3.46	0.2	10.34	282	12.23 ± 6.56	0.1	10.97
ESC_TAD_borders	Shift (DNA-protein complex) (30)	1058	-11.88 ± 2.08	-2.9	-5.12	288	-14.57 ± 5.09	-0.9	-9.11
ESC_TAD_borders	Hydrophilicity (RNA) (31)	651	16.32 ± 4.49	0.1	15.48	213	20.63 ± 9.33	0.0	20.32
ESC_TAD_borders	Rise (DNA-protein complex) (32)	1002	8.65 ± 2.05	0.3	7.94	205	14.59 ± 6.21	-0.0	14.94
ESC_TAD_borders	Stacking energy (33)	1008	-490.01 ± 108.81	0.1	-499.41	226	-815.08 ± 329.21	0.2	-900.56
ESC_TAD_borders	Free energy (34)	1009	-104.37 ± 23.99	-0.1	-101.92	227	-180.22 ± 71.76	0.0	-184.35
ESC_TAD_borders	Free energy (35)	989	-142.06 ± 27.22	-0.1	-136.33	231	-255.22 ± 96.76	-0.0	-249.81
ESC_TAD_borders	Free energy (36)	1011	-94.15 ± 21.12	-0.1	-92.3	227	-162.58 ± 64.14	0.1	-167.51
ESC_TAD_borders	Twist (DNA-protein complex) (37)	864	-57.23 ± 20.56	-1.0	-34.84	145	-128.81 ± 55.03	-0.9	-69.71
ESC_TAD_borders	Free energy (38)	1010	-111.47 ± 25.01	-0.1	-109.39	231	-188.94 ± 75.47	0.1	-197.69
ESC_TAD_borders	Twist_twist (39)	891	-0.35 ± 0.08	-0.4	-0.3	108	-0.36 ± 0.21	1.0	-0.7
ESC_TAD_borders	Tilt_tilt (40)	820	0.21 ± 0.08	0.7	0.15	256	0.42 ± 0.18	0.5	0.3
ESC_TAD_borders	Roll_roll (41)	976	0.11 ± 0.03	1.3	0.07	236	0.18 ± 0.09	0.5	0.14
ESC_TAD_borders	Twist_tilt (42)	973	-0.33 ± 0.09	-0.9	-0.23	155	-0.57 ± 0.26	-0.2	-0.51
ESC_TAD_borders	Twist_roll (43)	1001	-0.35 ± 0.08	-0.0	-0.34	218	-0.64 ± 0.26	-0.0	-0.62
ESC_TAD_borders	Tilt_roll (44)	964	-0.25 ± 0.07	-0.6	-0.2	190	-0.48 ± 0.21	-0.3	-0.4
ESC_TAD_borders	Shift_shift (45)	907	20.15 ± 4.14	3.9	3.58	453	26.7 ± 9.21	2.0	7.02
ESC_TAD_borders	Slide_slide (46)	1048	-11.36 ± 2.16	-0.1	-11.08	526	-8.46 ± 3.58	0.0	-8.61
ESC_TAD_borders	Rise_rise (47)	1029	-66.2 ± 9.82	-	-25.81	435	-83.0 ± 23.77	-1.0	-50.1

				3.3					
ESC_TAD_borders	Shift_slide (48)	734	-8.33 ± 2.71	-1.6	-3.53	29	-5.94 ± 4.57	-0.3	-4.17
ESC_TAD_borders	Shift_rise (49)	465	6.38 ± 1.5	3.4	1.12	455	8.64 ± 2.61	1.9	2.52
ESC_TAD_borders	Slide_rise (50)	431	14.28 ± 4.23	2.8	2.34	165	-14.38 ± 7.77	-2.0	1.74
ESC_TAD_borders	Twist_shift (51)	909	-2.27 ± 0.58	-1.6	-1.22	107	-2.68 ± 1.62	-0.2	-2.35
ESC_TAD_borders	Twist_slide (52)	752	-4.56 ± 0.95	-3.3	-1.26	509	-5.24 ± 1.68	-1.4	-2.4
ESC_TAD_borders	Twist_rise (53)	1168	-2.23 ± 0.25	-5.9	-0.54	986	-1.41 ± 0.37	-2.1	-0.31
ESC_TAD_borders	Tilt_shift (54)	1002	-6.89 ± 1.48	-0.2	-6.57	233	-12.34 ± 4.87	-0.1	-11.93
ESC_TAD_borders	Tilt_slide (55)	933	0.56 ± 0.17	0.5	0.45	217	1.2 ± 0.47	0.5	0.9
ESC_TAD_borders	Tilt_rise (56)	987	7.62 ± 1.66	0.7	6.04	149	10.31 ± 5.29	0.0	10.05
ESC_TAD_borders	Roll_shift (57)	978	-1.14 ± 0.33	-1.2	-0.69	197	-2.01 ± 0.97	-0.7	-1.24
ESC_TAD_borders	Roll_slide (58)	1028	2.25 ± 0.47	0.5	1.9	218	3.46 ± 1.49	-0.1	3.69
ESC_TAD_borders	Roll_rise (59)	985	3.67 ± 0.7	0.1	3.61	197	6.88 ± 2.7	0.1	6.66
ESC_TAD_borders	Stacking energy (60)	799	123.82 ± 39.64	0.9	80.75	213	280.63 ± 113.61	0.6	191.48
ESC_TAD_borders	Twist (61)	895	-198.58 ± 26.54	-4.7	-54.23	648	-183.35 ± 39.91	-1.3	-101.99
ESC_TAD_borders	Tilt (62)	920	24.16 ± 6.86	-0.5	28.62	208	47.6 ± 23.05	-0.1	51.48
ESC_TAD_borders	Roll (63)	982	236.85 ± 52.52	1.5	142.36	307	347.86 ± 126.36	0.4	284.64
ESC_TAD_borders	Shift (64)	623	3.4 ± 1.06	1.1	2.12	14	3.36 ± 2.69	-0.4	4.47
ESC_TAD_borders	Slide (65)	1004	64.5 ± 15.7	1.0	44.08	182	101.71 ± 48.32	-0.1	105.11
ESC_TAD_borders	Rise (66)	940	7.15 ± 2.09	-0.4	8.35	194	17.0 ± 7.24	0.2	15.33
ESC_TAD_borders	Slide stiffness (67)	1002	-53.29 ± 12.43	-0.9	-39.85	262	-91.86 ± 36.0	-0.5	-69.07
ESC_TAD_borders	Shift stiffness (68)	646	14.4 ± 3.35	4.2	0.41	758	12.57 ± 3.77	3.2	0.34
ESC_TAD_borders	Roll stiffness (69)	1096	0.06 ± 0.01	1.0	0.05	384	0.07 ± 0.03	0.0	0.07
ESC_TAD_borders	Tilt stiffness (70)	925	0.47 ± 0.12	0.5	0.39	257	0.84 ± 0.35	0.4	0.68
ESC_TAD_borders	Twist stiffness (71)	594	-0.15 ± 0.04	-0.5	-0.13	235	-0.3 ± 0.11	0.3	-0.36
ESC_TAD_borders	Free energy (72)	1008	-124.89 ± 27.42	-0.1	-121.16	233	-213.45 ± 83.38	0.1	-219.65
ESC_TAD_borders	Free energy (73)	1005	-113.89 ± 25.56	-0.1	-111.29	230	-196.4 ± 77.92	0.0	-201.56
ESC_TAD_borders	Free energy (74)	1002	-97.87 ± 22.42	0.0	-98.78	222	-171.3 ± 68.9	0.1	-178.76
ESC_TAD_borders	Free energy (75)	1001	-99.55 ± 24.32	0.0	-100.26	223	-180.18 ± 73.53	0.0	-181.37
ESC_TAD_borders	GC content (76)	1004	195.74 ± 43.56	0.0	195.72	225	353.92 ± 143.15	-0.0	354.11
ESC_TAD_borders	Purine (AG) content (77)	375	54.65 ± 19.17	0.0	54.48	412	-63.7 ± 28.56	-0.0	-63.64
ESC_TAD_borders	Keto (GT) content (78)	487	-50.98 ± 19.95	-0.1	-49.44	324	-53.76 ± 23.28	-0.1	-50.54
ESC_TAD_borders	Adenine content (79)	942	-103.77 ± 26.78	-0.0	-103.56	201	-171.84 ± 71.06	-0.0	-171.67
ESC_TAD_borders	Guanine content (80)	1011	108.32 ± 23.84	0.0	108.32	244	183.18 ± 70.31	-0.0	183.37
ESC_TAD_borders	Cytosine content (81)	961	102.76 ± 24.11	0.0	102.69	206	170.73 ± 75.04	-0.0	170.74
ESC_TAD_borders	Thymine content (82)	980	-123.84 ±	-	-123.81	274	-220.9 ± 77.08	0.0	-221.06

			30.72	0.0					
ESC_TAD_borders	Tilt (DNA-protein complex) (83)	820	55.28 ± 14.41	0.8	40.8	151	102.4 ± 41.93	0.1	94.0
ESC_TAD_borders	Roll (DNA-protein complex) (84)	989	187.41 ± 45.48	0.2	175.05	188	352.55 ± 154.3	0.1	341.4
ESC_TAD_borders	Shift (DNA-protein complex) (85)	1045	10.15 ± 2.0	3.1	3.38	274	11.01 ± 3.67	1.0	6.56
ESC_TAD_borders	Slide (DNA-protein complex) (86)	938	6.2 ± 1.51	-1.2	9.43	114	8.2 ± 4.97	-1.0	16.65
ESC_TAD_borders	Rise (DNA-protein complex) (87)	980	8.01 ± 2.04	-0.2	8.46	206	16.22 ± 6.46	0.1	15.38
ESC_TAD_borders	Twist (88)	1002	-241.11 ± 51.36	-0.6	-202.43	254	-415.97 ± 160.95	-0.2	-368.57
ESC_TAD_borders	Tilt (89)	1019	-79.33 ± 20.16	-2.0	-34.71	328	-118.47 ± 47.56	-0.7	-78.99
ESC_TAD_borders	Roll (90)	935	223.94 ± 60.58	0.2	211.13	211	488.2 ± 185.98	0.5	368.59
ESC_TAD_borders	Slide (91)	1010	57.15 ± 13.0	0.8	43.92	190	93.0 ± 41.34	0.1	85.8
ESC_TAD_borders	Twist (92)	997	-103.31 ± 23.95	-3.1	-24.23	299	-169.74 ± 60.35	-1.8	-52.72
ESC_TAD_borders	Tilt (93)	962	139.53 ± 39.35	1.0	93.24	242	267.5 ± 107.42	0.6	185.49
ESC_TAD_borders	Roll (94)	959	207.6 ± 61.46	1.2	125.19	215	427.75 ± 164.94	0.9	250.07
ESC_TAD_borders	Shift (95)	659	-7.12 ± 2.29	-2.9	-0.5	457	-8.95 ± 3.51	-2.3	-1.0
ESC_TAD_borders	Slide (96)	1001	68.12 ± 15.88	0.6	55.82	186	119.16 ± 52.25	0.1	109.53
ESC_TAD_borders	Rise (97)	995	8.63 ± 1.86	0.1	8.43	217	16.72 ± 6.47	0.1	15.49
ESC_TAD_borders	Twist (98)	939	-104.03 ± 27.17	-1.6	-53.41	200	-154.54 ± 68.29	-0.5	-116.17
ESC_TAD_borders	Wedge (99)	323	-91.28 ± 35.69	-1.9	-22.28	374	-147.74 ± 79.54	-1.0	-65.8
ESC_TAD_borders	Direction (100)	922	5921.7 ± 1343.2	1.1	4112.59	374	8510.99 ± 3370.45	0.6	5617.53
ESC_TAD_borders	Slide (RNA) (101)	974	-32.05 ± 8.13	0.4	-36.41	202	-64.36 ± 26.25	0.0	-65.9
ESC_TAD_borders	Rise (RNA) (102)	840	6.32 ± 2.01	-0.4	7.33	205	14.39 ± 5.56	0.2	12.72
ESC_TAD_borders	Tilt (RNA) (103)	815	60.52 ± 16.75	0.9	42.04	47	68.92 ± 45.12	-0.4	90.97
ESC_TAD_borders	Roll (RNA) (104)	882	121.96 ± 40.07	-0.8	164.86	182	332.73 ± 130.4	0.3	284.51
ESC_TAD_borders	Twist (RNA) (105)	745	-64.8 ± 8.27	-2.9	-30.58	807	-39.41 ± 11.23	-0.4	-30.06
ESC_TAD_borders	Stacking energy (RNA) (106)	954	133.4 ± 37.7	1.0	89.53	202	251.2 ± 108.64	0.6	177.48
ESC_TAD_borders	Rise stiffness (107)	686	16.32 ± 6.02	-0.3	18.65	126	44.97 ± 20.62	-0.2	51.91
ESC_TAD_borders	Melting Temperature (108)	1012	12.91 ± 2.89	0.1	12.66	231	21.85 ± 8.72	-0.1	22.88
ESC_TAD_borders	Stacking energy (109)	1010	49.47 ± 10.76	-0.1	50.79	226	81.52 ± 32.6	-0.2	91.61
ESC_TAD_borders	Enthalpy (RNA) (110)	994	-500.07 ± 117.8	0.1	-509.08	223	-922.59 ± 375.93	-0.0	-917.36
ESC_TAD_borders	Entropy (RNA) (111)	994	-1011.3 ± 241.25	0.1	-1038.15	222	-1864.29 ± 759.15	0.0	-1866.95
ESC_TAD_borders	Free energy (RNA) (112)	1002	-183.41 ± 42.29	0.0	-183.73	223	-335.97 ± 137.34	-0.0	-331.9
ESC_TAD_borders	Free energy (RNA) (113)	998	-196.43 ± 46.84	0.0	-199.23	217	-366.3 ± 151.36	-0.0	-359.93
ESC_TAD_borders	Enthalpy (RNA) (114)	991	-506.1 ±	0.1	-522.98	203	-981.92 ±	0.0	-991.69

		132.07				417.21		
ESC_TAD_borders Entropy (RNA) (115)	987	-1011.72 ± 272.83	0.2	-1075.78	195	-1986.41 ± 858.01	0.0	-2037.27
ESC_TAD_borders Roll (116)	995	427.15 ± 95.99	0.4	372.45	166	696.93 ± 313.9	-0.1	719.22
ESC_TAD_borders Tilt (117)	385	-67.09 ± 24.51	-0.1	-64.78	410	-78.67 ± 32.69	-0.1	-72.9
ESC_TAD_borders Twist (118)	1000	-216.15 ± 46.9	-0.2	-201.55	209	-400.58 ± 163.51	-0.0	-396.13
ESC_TAD_borders Roll (119)	1083	165.08 ± 25.25	1.2	120.59	378	173.17 ± 66.57	-0.4	213.62
ESC_TAD_borders Twist (120)	1033	-263.13 ± 51.23	-1.2	-185.73	221	-363.12 ± 151.93	-0.0	-360.66
ESC_TAD_borders Flexibility_slide (121)	1005	-616.9 ± 134.92	-0.3	-557.93	192	-1058.49 ± 457.69	0.1	-1091.0
ESC_TAD_borders Flexibility_shift (122)	1082	372.67 ± 59.23	1.1	275.29	346	411.43 ± 145.32	-0.3	489.41
ESC_TAD_borders Enthalpy (123)	1027	-171.25 ± 32.19	-0.1	-165.83	252	-261.17 ± 99.59	0.2	-299.85
ESC_TAD_borders Entropy (124)	1073	-186.36 ± 25.38	-0.2	-176.97	297	-212.34 ± 72.26	0.7	-319.16
ESC_TAD_borders Free energy (125)	1005	-113.89 ± 25.56	-0.1	-111.29	230	-196.4 ± 77.92	0.0	-201.56

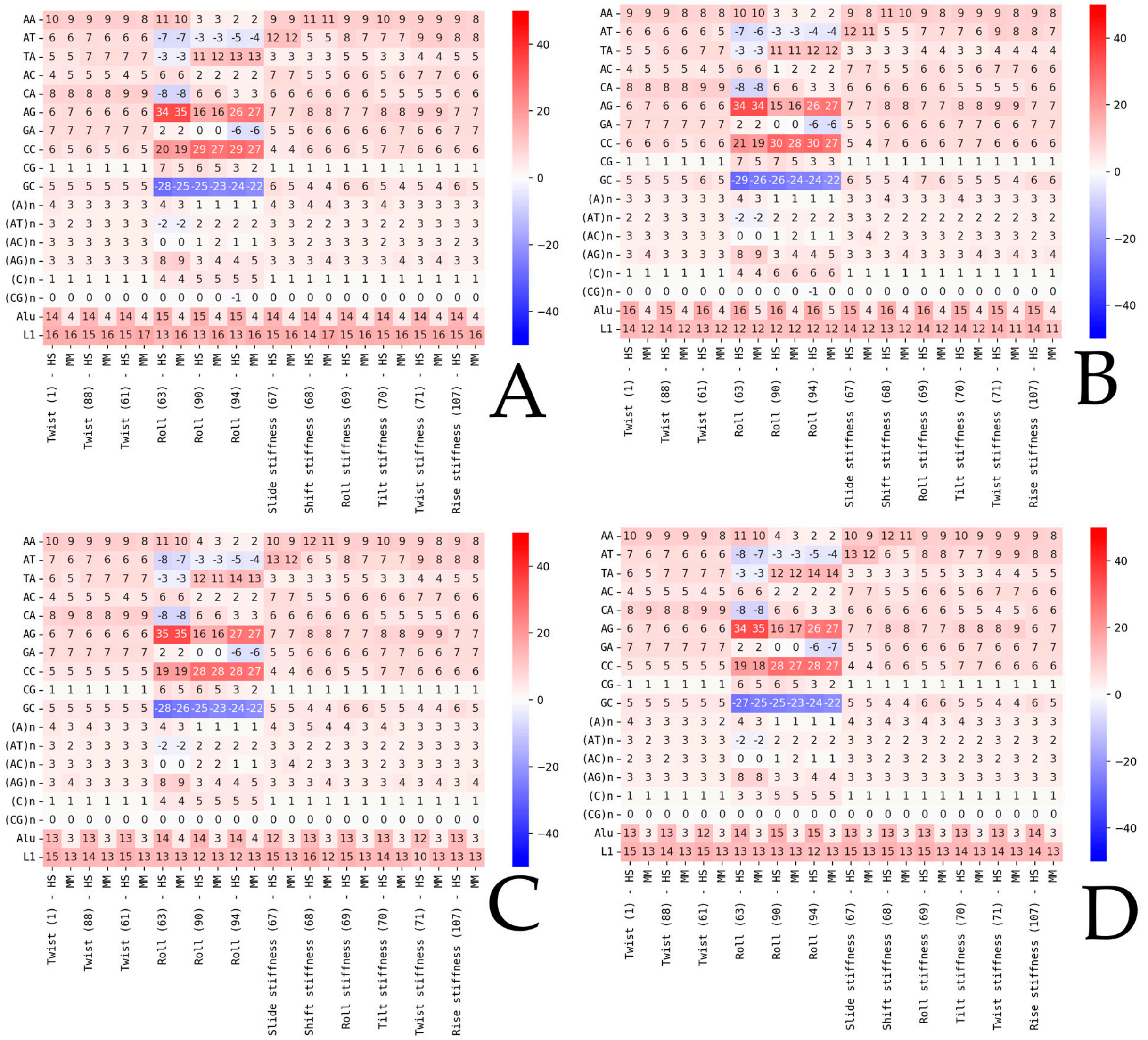

Figure S1: Relative Influence of Dinucleotides/Repeats on DNA Properties around Functional Annotations: Relative influences (in percent [%]) of representative selection of dinucleotides/repeats on peaks of structural DNA properties around annotations. (A): around genes, (B): around promoters, (C); around enhancer, (D); around TAD borders (see Fig. 5 for CTCF, Table S4 for other dinucleotides/repeats).

Table S4: Correlation between DNA Properties and Functional Annotations (10 kbp Resolution): Representative selection of correlation values between genomic maps with 10 kbp resolution of functional annotations, derived based on from genbank files [47], and ensemble data files and genomic maps of DNA properties, derived based on dinucleotide maps, using models from the Dinucleotide Properties Genome Browser [52] (see Table S1 for complete dataset).

model	genes (HS)	genes (MM)	Promoter (HS)	Promoter (MM)	Enhancer (HS)	Enhancer (MM)	CTCF Binding Site (HS)	CTCF Binding Site (MM)
Twist (1)	-0.18 ± 0.21	-0.26 ± 0.16	-0.3 ± 0.17	-0.22 ± 0.18	-0.07 ± 0.18	-0.29 ± 0.14	-0.22 ± 0.19	-0.3 ± 0.14
Twist (DNA-protein complex) (26)	-0.24 ± 0.2	-0.24 ± 0.18	-0.28 ± 0.19	-0.23 ± 0.23	-0.19 ± 0.18	-0.27 ± 0.19	-0.38 ± 0.11	-0.31 ± 0.22
Twist (DNA-protein complex) (37)	-0.27 ± 0.17	-0.25 ± 0.19	-0.29 ± 0.23	-0.19 ± 0.24	-0.16 ± 0.19	-0.28 ± 0.16	-0.43 ± 0.2	-0.33 ± 0.17
Twist (88)	-0.33 ± 0.18	-0.38 ± 0.16	-0.4 ± 0.21	-0.21 ± 0.19	-0.18 ± 0.17	-0.29 ± 0.23	-0.39 ± 0.23	-0.36 ± 0.09
Twist (61)	-0.27 ± 0.17	-0.28 ± 0.21	-0.24 ± 0.29	-0.1 ± 0.17	-0.2 ± 0.19	-0.18 ± 0.22	-0.23 ± 0.23	-0.13 ± 0.16
Twist (98)	-0.31 ± 0.16	-0.26 ± 0.17	-0.24 ± 0.18	-0.13 ± 0.17	-0.23 ± 0.21	-0.35 ± 0.12	-0.38 ± 0.11	-0.33 ± 0.14
Twist (118)	-0.29 ± 0.21	-0.27 ± 0.17	-0.27 ± 0.21	-0.13 ± 0.16	-0.25 ± 0.2	-0.29 ± 0.2	-0.43 ± 0.19	-0.35 ± 0.17
Twist (120)	-0.28 ± 0.21	-0.26 ± 0.17	-0.25 ± 0.27	-0.21 ± 0.12	-0.17 ± 0.18	-0.32 ± 0.2	-0.38 ± 0.19	-0.31 ± 0.16
Twist (92)	-0.18 ± 0.25	-0.2 ± 0.18	-0.3 ± 0.21	-0.18 ± 0.19	-0.2 ± 0.13	-0.26 ± 0.15	-0.39 ± 0.24	-0.25 ± 0.17
Twist_twist (39)	-0.19 ± 0.22	-0.2 ± 0.14	-0.13 ± 0.2	-0.19 ± 0.15	-0.13 ± 0.16	-0.16 ± 0.19	-0.27 ± 0.17	-0.24 ± 0.18
Twist_tilt (42)	-0.23 ± 0.19	-0.16 ± 0.19	-0.29 ± 0.2	-0.26 ± 0.18	-0.2 ± 0.13	-0.26 ± 0.17	-0.35 ± 0.22	-0.28 ± 0.14
Twist_shift (51)	-0.16 ± 0.15	-0.17 ± 0.2	-0.21 ± 0.18	-0.08 ± 0.15	-0.23 ± 0.16	-0.25 ± 0.2	-0.35 ± 0.19	-0.27 ± 0.22
Twist_slide (52)	-0.22 ± 0.17	-0.17 ± 0.17	-0.35 ± 0.14	-0.1 ± 0.16	-0.09 ± 0.17	-0.04 ± 0.18	-0.28 ± 0.15	-0.08 ± 0.12
Twist_rise (53)	-0.1 ± 0.2	-0.16 ± 0.19	-0.14 ± 0.2	-0.17 ± 0.18	-0.09 ± 0.17	-0.31 ± 0.14	-0.17 ± 0.19	-0.32 ± 0.2
Twist_roll (43)	-0.35 ± 0.2	-0.31 ± 0.18	-0.34 ± 0.19	-0.16 ± 0.16	-0.26 ± 0.18	-0.32 ± 0.16	-0.5 ± 0.21	-0.41 ± 0.17
Roll (DNA-protein complex) (25)	0.28 ± 0.16	0.27 ± 0.12	0.26 ± 0.19	0.18 ± 0.2	0.29 ± 0.2	0.35 ± 0.16	0.42 ± 0.19	0.4 ± 0.13

Roll (63)	0.26 ± 0.23	0.27 ± 0.19	0.32 ± 0.23	0.15 ± 0.15	0.22 ± 0.18	0.18 ± 0.22	0.46 ± 0.16	0.28 ± 0.18
Roll (90)	0.22 ± 0.22	0.28 ± 0.18	0.43 ± 0.2	0.2 ± 0.19	0.11 ± 0.19	0.23 ± 0.17	0.43 ± 0.21	0.29 ± 0.18
Roll (94)	0.2 ± 0.19	0.22 ± 0.21	0.25 ± 0.2	0.21 ± 0.14	0.21 ± 0.15	0.27 ± 0.17	0.46 ± 0.2	0.29 ± 0.2
Roll (116)	0.19 ± 0.2	0.29 ± 0.18	0.22 ± 0.19	0.25 ± 0.16	0.25 ± 0.15	0.26 ± 0.13	0.37 ± 0.14	0.35 ± 0.16
Roll (119)	0.23 ± 0.2	0.29 ± 0.18	0.26 ± 0.19	0.22 ± 0.15	0.24 ± 0.2	0.35 ± 0.11	0.38 ± 0.16	0.34 ± 0.12
Roll (DNA-protein complex) (84)	0.26 ± 0.21	0.3 ± 0.15	0.33 ± 0.19	0.13 ± 0.21	0.17 ± 0.18	0.35 ± 0.19	0.52 ± 0.17	0.33 ± 0.19
Roll_roll (41)	0.23 ± 0.16	0.26 ± 0.18	0.21 ± 0.2	0.15 ± 0.2	0.21 ± 0.19	0.27 ± 0.14	0.33 ± 0.2	0.34 ± 0.15
Roll_shift (57)	-0.17 ± 0.22	-0.24 ± 0.17	-0.26 ± 0.2	-0.23 ± 0.16	-0.19 ± 0.17	-0.31 ± 0.17	-0.41 ± 0.24	-0.36 ± 0.18
Roll_slide (58)	0.29 ± 0.15	0.26 ± 0.19	0.23 ± 0.2	0.22 ± 0.18	0.18 ± 0.19	0.36 ± 0.16	0.46 ± 0.14	0.4 ± 0.19
Roll_rise (59)	0.27 ± 0.16	0.34 ± 0.21	0.34 ± 0.21	0.2 ± 0.18	0.2 ± 0.16	0.35 ± 0.21	0.41 ± 0.19	0.33 ± 0.2
Tilt_roll (44)	-0.17 ± 0.21	-0.23 ± 0.18	-0.27 ± 0.2	-0.22 ± 0.23	-0.14 ± 0.18	-0.3 ± 0.14	-0.46 ± 0.21	-0.32 ± 0.15
Tilt (DNA-protein complex) (27)	-0.2 ± 0.19	-0.17 ± 0.15	-0.27 ± 0.18	-0.12 ± 0.15	-0.13 ± 0.2	-0.23 ± 0.18	-0.25 ± 0.19	-0.24 ± 0.19
Tilt (62)	0.26 ± 0.15	0.22 ± 0.2	0.26 ± 0.22	0.24 ± 0.14	0.22 ± 0.14	0.28 ± 0.16	0.33 ± 0.22	0.29 ± 0.15
Tilt (89)	-0.22 ± 0.19	-0.24 ± 0.18	-0.31 ± 0.23	-0.14 ± 0.16	-0.26 ± 0.14	-0.36 ± 0.15	-0.44 ± 0.2	-0.33 ± 0.18
Tilt (93)	0.23 ± 0.19	0.2 ± 0.25	0.27 ± 0.22	0.13 ± 0.19	0.2 ± 0.18	0.28 ± 0.16	0.42 ± 0.15	0.29 ± 0.18
Tilt (117)	-0.0 ± 0.17	0.03 ± 0.15	-0.02 ± 0.17	-0.05 ± 0.12	0.03 ± 0.17	-0.03 ± 0.15	0.03 ± 0.16	0.01 ± 0.14
Tilt (DNA-protein complex) (83)	0.18 ± 0.17	0.21 ± 0.2	0.19 ± 0.24	0.13 ± 0.23	0.13 ± 0.24	0.24 ± 0.13	0.37 ± 0.17	0.35 ± 0.22
Tilt_tilt (40)	0.18 ± 0.17	0.1 ± 0.22	0.29 ± 0.23	0.07 ± 0.24	0.13 ± 0.2	0.23 ± 0.17	0.33 ± 0.22	0.2 ± 0.17
Tilt_shift (54)	-0.18 ± 0.19	-0.28 ± 0.18	-0.4 ± 0.18	-0.2 ± 0.16	-0.17 ± 0.18	-0.32 ± 0.13	-0.52 ± 0.18	-0.34 ± 0.21
Twist_tilt (42)	-0.23 ± 0.19	-0.16 ± 0.19	-0.29 ± 0.2	-0.26 ± 0.18	-0.2 ± 0.13	-0.26 ± 0.17	-0.35 ± 0.22	-0.28 ± 0.14
Tilt_rise (56)	0.28 ± 0.09	0.34 ± 0.19	0.26 ± 0.17	0.12 ± 0.18	0.24 ± 0.16	0.39 ± 0.18	0.38 ± 0.19	0.36 ± 0.12
Tilt_slide (55)	0.22 ± 0.18	0.23 ± 0.16	0.33 ± 0.22	0.26 ± 0.23	0.22 ± 0.12	0.28 ± 0.19	0.43 ± 0.14	0.41 ± 0.19
Slide (DNA-protein complex) (28)	0.17 ± 0.19	0.21 ± 0.17	0.33 ± 0.18	0.06 ± 0.17	0.11 ± 0.19	0.15 ± 0.2	0.27 ± 0.21	0.21 ± 0.19

Slide (65)	0.15 ± 0.19	0.19 ± 0.18	0.14 ± 0.13	0.18 ± 0.17	0.18 ± 0.14	0.31 ± 0.18	0.39 ± 0.21	0.33 ± 0.17
Slide (91)	0.31 ± 0.18	0.25 ± 0.15	0.26 ± 0.2	0.26 ± 0.17	0.2 ± 0.19	0.32 ± 0.16	0.43 ± 0.2	0.36 ± 0.13
Slide (96)	0.24 ± 0.2	0.27 ± 0.17	0.29 ± 0.18	0.14 ± 0.2	0.17 ± 0.19	0.23 ± 0.18	0.47 ± 0.19	0.34 ± 0.18
Slide (DNA-protein complex) (86)	0.1 ± 0.18	0.17 ± 0.15	0.16 ± 0.13	0.11 ± 0.15	0.16 ± 0.19	0.3 ± 0.14	0.32 ± 0.16	0.32 ± 0.19
Slide_slide (46)	-0.1 ± 0.17	-0.2 ± 0.2	-0.02 ± 0.23	-0.1 ± 0.14	-0.1 ± 0.17	-0.31 ± 0.14	-0.05 ± 0.23	-0.21 ± 0.18
Slide_rise (50)	-0.16 ± 0.17	-0.08 ± 0.2	-0.09 ± 0.28	-0.07 ± 0.14	-0.16 ± 0.21	-0.08 ± 0.18	-0.31 ± 0.2	-0.12 ± 0.12
Twist_slide (52)	-0.22 ± 0.17	-0.17 ± 0.17	-0.35 ± 0.14	-0.1 ± 0.16	-0.09 ± 0.17	-0.04 ± 0.18	-0.28 ± 0.15	-0.08 ± 0.12
Roll_slide (58)	0.29 ± 0.15	0.26 ± 0.19	0.23 ± 0.2	0.22 ± 0.18	0.18 ± 0.19	0.36 ± 0.16	0.46 ± 0.14	0.4 ± 0.19
Shift_slide (48)	-0.04 ± 0.18	-0.08 ± 0.22	-0.08 ± 0.21	-0.17 ± 0.16	-0.11 ± 0.14	-0.22 ± 0.21	-0.26 ± 0.19	-0.24 ± 0.19
Shift (DNA-protein complex) (30)	-0.29 ± 0.22	-0.28 ± 0.2	-0.25 ± 0.22	-0.19 ± 0.18	-0.22 ± 0.19	-0.35 ± 0.17	-0.42 ± 0.17	-0.34 ± 0.14
Shift (64)	0.17 ± 0.18	0.12 ± 0.15	0.08 ± 0.18	0.17 ± 0.2	0.14 ± 0.18	0.08 ± 0.17	0.24 ± 0.22	0.19 ± 0.22
Shift (95)	-0.19 ± 0.26	-0.12 ± 0.16	-0.21 ± 0.15	-0.16 ± 0.21	-0.26 ± 0.2	-0.23 ± 0.16	-0.28 ± 0.25	-0.25 ± 0.16
Shift (DNA-protein complex) (85)	0.26 ± 0.19	0.2 ± 0.19	0.16 ± 0.19	0.2 ± 0.18	0.17 ± 0.18	0.32 ± 0.18	0.42 ± 0.2	0.35 ± 0.18
Shift_shift (45)	0.22 ± 0.21	0.25 ± 0.23	0.32 ± 0.2	0.15 ± 0.16	0.16 ± 0.19	0.15 ± 0.16	0.38 ± 0.17	0.29 ± 0.23
Twist_shift (51)	-0.16 ± 0.15	-0.17 ± 0.2	-0.21 ± 0.18	-0.08 ± 0.15	-0.23 ± 0.16	-0.25 ± 0.2	-0.35 ± 0.19	-0.27 ± 0.22
Tilt_shift (54)	-0.18 ± 0.19	-0.28 ± 0.18	-0.4 ± 0.18	-0.2 ± 0.16	-0.17 ± 0.18	-0.32 ± 0.13	-0.52 ± 0.18	-0.34 ± 0.21
Roll_shift (57)	-0.17 ± 0.22	-0.24 ± 0.17	-0.26 ± 0.2	-0.23 ± 0.16	-0.19 ± 0.17	-0.31 ± 0.17	-0.41 ± 0.24	-0.36 ± 0.18
Shift_rise (49)	0.21 ± 0.18	0.11 ± 0.2	0.32 ± 0.24	0.08 ± 0.22	0.06 ± 0.2	-0.09 ± 0.19	0.35 ± 0.22	0.01 ± 0.18
Rise (3)	0.31 ± 0.21	0.25 ± 0.16	0.32 ± 0.23	0.17 ± 0.18	0.14 ± 0.15	0.18 ± 0.19	0.43 ± 0.17	0.37 ± 0.23
Rise (DNA-protein complex) (32)	0.25 ± 0.16	0.19 ± 0.17	0.31 ± 0.18	0.22 ± 0.22	0.11 ± 0.15	0.32 ± 0.2	0.38 ± 0.16	0.41 ± 0.16
Rise (66)	0.21 ± 0.22	0.28 ± 0.21	0.39 ± 0.22	0.26 ± 0.19	0.16 ± 0.13	0.29 ± 0.22	0.47 ± 0.14	0.25 ± 0.17
Rise (97)	0.28 ± 0.26	0.36 ± 0.25	0.34 ± 0.22	0.25 ± 0.22	0.1 ± 0.15	0.33 ± 0.18	0.53 ± 0.13	0.32 ± 0.19
Rise (DNA-protein complex) (87)	0.23 ± 0.2	0.21 ± 0.19	0.36 ± 0.17	0.22 ± 0.19	0.2 ± 0.17	0.38 ± 0.12	0.45 ± 0.21	0.38 ± 0.16

Rise_rise (47)	-0.31 ± 0.19	-0.38 ± 0.13	-0.39 ± 0.21	-0.22 ± 0.15	-0.22 ± 0.17	-0.25 ± 0.17	-0.39 ± 0.24	-0.34 ± 0.18
Slide_rise (50)	-0.16 ± 0.17	-0.08 ± 0.2	-0.09 ± 0.28	-0.07 ± 0.14	-0.16 ± 0.21	-0.08 ± 0.18	-0.31 ± 0.2	-0.12 ± 0.12
Twist_rise (53)	-0.1 ± 0.2	-0.16 ± 0.19	-0.14 ± 0.2	-0.17 ± 0.18	-0.09 ± 0.17	-0.31 ± 0.14	-0.17 ± 0.19	-0.32 ± 0.2
Tilt_rise (56)	0.28 ± 0.09	0.34 ± 0.19	0.26 ± 0.17	0.12 ± 0.18	0.24 ± 0.16	0.39 ± 0.18	0.38 ± 0.19	0.36 ± 0.12
Roll_rise (59)	0.27 ± 0.16	0.34 ± 0.21	0.34 ± 0.21	0.2 ± 0.18	0.2 ± 0.16	0.35 ± 0.21	0.41 ± 0.19	0.33 ± 0.2
Bend (4)	-0.29 ± 0.13	-0.26 ± 0.18	-0.37 ± 0.18	-0.23 ± 0.18	-0.23 ± 0.14	-0.33 ± 0.18	-0.45 ± 0.16	-0.32 ± 0.16
Tip (5)	-0.2 ± 0.12	-0.31 ± 0.14	-0.28 ± 0.2	-0.08 ± 0.18	-0.22 ± 0.21	-0.27 ± 0.17	-0.31 ± 0.21	-0.33 ± 0.19
Inclination (6)	0.06 ± 0.15	0.05 ± 0.16	0.01 ± 0.09	-0.02 ± 0.11	0.02 ± 0.13	-0.06 ± 0.18	-0.02 ± 0.15	-0.04 ± 0.18
Propeller Twist (20)	0.28 ± 0.2	0.22 ± 0.13	0.21 ± 0.16	0.17 ± 0.2	0.17 ± 0.26	0.4 ± 0.15	0.42 ± 0.16	0.4 ± 0.17
Stacking energy (2)	0.27 ± 0.23	0.18 ± 0.22	0.26 ± 0.2	0.17 ± 0.26	0.21 ± 0.22	0.1 ± 0.21	0.4 ± 0.18	0.1 ± 0.19
Stacking energy (33)	-0.3 ± 0.16	-0.31 ± 0.18	-0.35 ± 0.16	-0.2 ± 0.13	-0.11 ± 0.15	-0.32 ± 0.19	-0.41 ± 0.18	-0.36 ± 0.21
Stacking energy (60)	0.21 ± 0.24	0.27 ± 0.22	0.31 ± 0.19	0.17 ± 0.17	0.18 ± 0.18	0.23 ± 0.16	0.38 ± 0.19	0.26 ± 0.12
Stacking energy (109)	0.3 ± 0.19	0.29 ± 0.18	0.34 ± 0.22	0.23 ± 0.25	0.2 ± 0.15	0.31 ± 0.12	0.45 ± 0.15	0.36 ± 0.17
Melting Temperature (16)	0.31 ± 0.18	0.35 ± 0.22	0.27 ± 0.19	0.28 ± 0.21	0.2 ± 0.16	0.3 ± 0.2	0.34 ± 0.15	0.39 ± 0.16
Melting Temperature (108)	0.29 ± 0.17	0.25 ± 0.19	0.34 ± 0.24	0.22 ± 0.21	0.24 ± 0.21	0.32 ± 0.17	0.39 ± 0.15	0.38 ± 0.18
Enthalpy (22)	-0.32 ± 0.17	-0.31 ± 0.15	-0.37 ± 0.23	-0.23 ± 0.21	-0.16 ± 0.15	-0.37 ± 0.15	-0.42 ± 0.21	-0.34 ± 0.19
Enthalpy (123)	-0.29 ± 0.22	-0.32 ± 0.17	-0.36 ± 0.21	-0.22 ± 0.16	-0.2 ± 0.18	-0.35 ± 0.16	-0.46 ± 0.17	-0.37 ± 0.2
Entropy (23)	-0.34 ± 0.21	-0.26 ± 0.23	-0.36 ± 0.19	-0.21 ± 0.2	-0.29 ± 0.17	-0.3 ± 0.14	-0.42 ± 0.19	-0.36 ± 0.22
Entropy (124)	-0.25 ± 0.21	-0.3 ± 0.19	-0.51 ± 0.21	-0.24 ± 0.16	-0.12 ± 0.19	-0.29 ± 0.11	-0.39 ± 0.16	-0.36 ± 0.16
Free energy (34)	-0.26 ± 0.22	-0.29 ± 0.15	-0.34 ± 0.15	-0.19 ± 0.18	-0.22 ± 0.15	-0.31 ± 0.2	-0.5 ± 0.15	-0.36 ± 0.15
Free energy (35)	-0.28 ± 0.18	-0.31 ± 0.21	-0.3 ± 0.17	-0.25 ± 0.17	-0.15 ± 0.2	-0.35 ± 0.15	-0.43 ± 0.12	-0.39 ± 0.19
Free energy (36)	-0.28 ± 0.18	-0.31 ± 0.2	-0.31 ± 0.23	-0.15 ± 0.16	-0.26 ± 0.15	-0.31 ± 0.17	-0.45 ± 0.24	-0.38 ± 0.17
Free energy (38)	-0.31 ± 0.18	-0.22 ± 0.25	-0.32 ± 0.2	-0.22 ± 0.19	-0.14 ± 0.14	-0.33 ± 0.23	-0.44 ± 0.18	-0.37 ± 0.11

Free energy (72)	-0.28 ± 0.14	-0.26 ± 0.21	-0.31 ± 0.19	-0.3 ± 0.18	-0.17 ± 0.17	-0.37 ± 0.14	-0.54 ± 0.14	-0.37 ± 0.16
Free energy (73)	-0.35 ± 0.18	-0.31 ± 0.22	-0.28 ± 0.21	-0.21 ± 0.13	-0.09 ± 0.13	-0.39 ± 0.16	-0.43 ± 0.16	-0.37 ± 0.12
Free energy (74)	-0.32 ± 0.17	-0.29 ± 0.18	-0.38 ± 0.26	-0.28 ± 0.23	-0.17 ± 0.18	-0.27 ± 0.13	-0.39 ± 0.16	-0.28 ± 0.17
Free energy (75)	-0.36 ± 0.17	-0.3 ± 0.2	-0.36 ± 0.21	-0.21 ± 0.21	-0.13 ± 0.16	-0.37 ± 0.19	-0.48 ± 0.16	-0.36 ± 0.11
Free energy (125)	-0.29 ± 0.2	-0.32 ± 0.17	-0.3 ± 0.17	-0.24 ± 0.25	-0.15 ± 0.19	-0.36 ± 0.13	-0.39 ± 0.2	-0.4 ± 0.19
Major Groove Width (7)	0.3 ± 0.17	0.25 ± 0.17	0.25 ± 0.27	0.15 ± 0.16	0.26 ± 0.16	0.27 ± 0.22	0.44 ± 0.21	0.39 ± 0.19
Major Groove Depth (8)	-0.16 ± 0.17	-0.32 ± 0.19	-0.35 ± 0.19	-0.22 ± 0.2	-0.18 ± 0.16	-0.3 ± 0.17	-0.35 ± 0.16	-0.39 ± 0.16
Major Groove Size (9)	-0.02 ± 0.19	-0.06 ± 0.16	-0.03 ± 0.21	-0.05 ± 0.2	0.03 ± 0.18	-0.05 ± 0.25	0.07 ± 0.19	0.02 ± 0.18
Major Groove Distance (10)	-0.18 ± 0.22	-0.19 ± 0.16	-0.24 ± 0.19	-0.16 ± 0.19	-0.17 ± 0.17	-0.11 ± 0.17	-0.3 ± 0.18	-0.19 ± 0.18
Mobility to bend towards major groove (18)	-0.25 ± 0.14	-0.19 ± 0.21	-0.2 ± 0.17	-0.22 ± 0.18	-0.16 ± 0.14	-0.26 ± 0.17	-0.47 ± 0.15	-0.37 ± 0.16
Minor Groove Width (11)	-0.23 ± 0.2	-0.24 ± 0.2	-0.27 ± 0.18	-0.32 ± 0.17	-0.2 ± 0.17	-0.39 ± 0.18	-0.42 ± 0.21	-0.33 ± 0.14
Minor Groove Depth (12)	0.2 ± 0.21	0.01 ± 0.13	0.19 ± 0.23	-0.04 ± 0.16	0.15 ± 0.13	-0.02 ± 0.17	0.24 ± 0.15	-0.04 ± 0.18
Minor Groove Size (13)	0.28 ± 0.21	0.23 ± 0.2	0.33 ± 0.18	0.24 ± 0.2	0.13 ± 0.18	0.36 ± 0.15	0.43 ± 0.21	0.34 ± 0.17
Minor Groove Distance (14)	-0.18 ± 0.16	-0.21 ± 0.18	-0.25 ± 0.23	-0.1 ± 0.2	-0.14 ± 0.15	-0.12 ± 0.18	-0.28 ± 0.17	-0.13 ± 0.14
Mobility to bend towards minor groove (19)	0.32 ± 0.16	0.25 ± 0.19	0.32 ± 0.2	0.19 ± 0.22	0.2 ± 0.2	0.33 ± 0.17	0.44 ± 0.15	0.33 ± 0.19
Probability contacting nucleosome core (17)	-0.32 ± 0.18	-0.19 ± 0.27	-0.31 ± 0.25	-0.15 ± 0.25	-0.08 ± 0.1	-0.31 ± 0.2	-0.41 ± 0.16	-0.3 ± 0.15
Persistance Length (15)	0.32 ± 0.24	0.3 ± 0.2	0.3 ± 0.21	0.18 ± 0.19	0.14 ± 0.2	0.33 ± 0.19	0.42 ± 0.17	0.41 ± 0.18
Slide stiffness (67)	-0.25 ± 0.2	-0.27 ± 0.22	-0.27 ± 0.22	-0.2 ± 0.15	-0.23 ± 0.23	-0.36 ± 0.19	-0.4 ± 0.16	-0.32 ± 0.2
Flexibility_slide (121)	-0.23 ± 0.19	-0.28 ± 0.17	-0.34 ± 0.19	-0.2 ± 0.17	-0.17 ± 0.16	-0.31 ± 0.14	-0.43 ± 0.11	-0.26 ± 0.17
Shift stiffness (68)	0.12 ± 0.2	-0.01 ± 0.14	0.12 ± 0.17	0.08 ± 0.11	0.09 ± 0.14	0.1 ± 0.19	0.15 ± 0.13	0.09 ± 0.15
Flexibility_shift (122)	0.29 ± 0.17	0.29 ± 0.19	0.39 ± 0.18	0.13 ± 0.2	0.21 ± 0.17	0.31 ± 0.13	0.45 ± 0.15	0.38 ± 0.17
Roll stiffness (69)	0.22 ± 0.21	0.31 ± 0.17	0.26 ± 0.2	0.27 ± 0.16	0.13 ± 0.15	0.4 ± 0.1	0.33 ± 0.17	0.34 ± 0.17
Tilt stiffness (70)	0.22 ± 0.13	0.21 ± 0.23	0.26 ± 0.19	0.14 ± 0.16	0.19 ± 0.19	0.19 ± 0.2	0.4 ± 0.17	0.29 ± 0.13

Twist stiffness (71)	-0.27 ± 0.23	-0.27 ± 0.3	-0.43 ± 0.21	-0.1 ± 0.2	-0.03 ± 0.18	-0.18 ± 0.21	-0.27 ± 0.24	-0.22 ± 0.2
Rise stiffness (107)	0.33 ± 0.19	0.14 ± 0.15	0.27 ± 0.22	0.13 ± 0.2	0.14 ± 0.2	0.25 ± 0.19	0.41 ± 0.16	0.31 ± 0.18

Table S5: Correlation between DNA Properties and Functional Annotations (1 Mbp Resolution): Representative selection of correlation values between genomic maps with 1 Mbp resolution of functional annotations, derived based on from genbank files [47], and ensemble data files and genomic maps of DNA properties, derived based on dinucleotide maps, using models from the Dinucleotide Properties Genome Browser [52] (see Table S1 for complete dataset).

model	genes (HS)	genes (MM)	Promoter (HS)	Promoter (MM)	Enhancer (HS)	Enhancer (MM)	CTCF Binding Site (HS)	CTCF Binding Site (MM)
Twist (1)	-0.57 ± 0.17	-0.61 ± 0.21	-0.78 ± 0.12	-0.8 ± 0.08	-0.32 ± 0.16	-0.75 ± 0.06	-0.75 ± 0.11	-0.89 ± 0.04
Twist (DNA-protein complex) (26)	-0.64 ± 0.11	-0.59 ± 0.15	-0.7 ± 0.08	-0.73 ± 0.06	-0.46 ± 0.11	-0.78 ± 0.09	-0.84 ± 0.11	-0.84 ± 0.09
Twist (DNA-protein complex) (37)	-0.68 ± 0.14	-0.61 ± 0.22	-0.77 ± 0.11	-0.71 ± 0.06	-0.48 ± 0.16	-0.69 ± 0.12	-0.88 ± 0.05	-0.86 ± 0.04
Twist (88)	-0.7 ± 0.14	-0.67 ± 0.13	-0.8 ± 0.08	-0.78 ± 0.08	-0.47 ± 0.18	-0.76 ± 0.11	-0.91 ± 0.04	-0.89 ± 0.04
Twist (61)	-0.72 ± 0.17	-0.66 ± 0.14	-0.84 ± 0.05	-0.77 ± 0.06	-0.58 ± 0.12	-0.66 ± 0.1	-0.82 ± 0.05	-0.79 ± 0.08
Twist (98)	-0.62 ± 0.13	-0.6 ± 0.21	-0.74 ± 0.08	-0.72 ± 0.08	-0.55 ± 0.13	-0.77 ± 0.1	-0.87 ± 0.03	-0.89 ± 0.03
Twist (118)	-0.64 ± 0.14	-0.59 ± 0.15	-0.76 ± 0.1	-0.74 ± 0.09	-0.48 ± 0.11	-0.82 ± 0.07	-0.91 ± 0.04	-0.88 ± 0.05
Twist (120)	-0.64 ± 0.11	-0.53 ± 0.16	-0.72 ± 0.1	-0.71 ± 0.1	-0.51 ± 0.12	-0.84 ± 0.06	-0.88 ± 0.04	-0.89 ± 0.04
Twist (92)	-0.72 ± 0.17	-0.74 ± 0.12	-0.81 ± 0.06	-0.78 ± 0.08	-0.55 ± 0.1	-0.72 ± 0.09	-0.9 ± 0.03	-0.9 ± 0.04
Twist_twist (39)	-0.53 ± 0.16	-0.5 ± 0.14	-0.63 ± 0.1	-0.54 ± 0.1	-0.46 ± 0.12	-0.84 ± 0.06	-0.75 ± 0.1	-0.8 ± 0.06
Twist_tilt (42)	-0.62 ± 0.15	-0.62 ± 0.13	-0.73 ± 0.09	-0.69 ± 0.11	-0.53 ± 0.1	-0.79 ± 0.08	-0.89 ± 0.04	-0.87 ± 0.04
Twist_shift (51)	-0.55 ± 0.11	-0.53 ± 0.14	-0.7 ± 0.1	-0.64 ± 0.08	-0.51 ± 0.12	-0.8 ± 0.06	-0.86 ± 0.05	-0.84 ± 0.05
Twist_slide (52)	-0.73 ± 0.13	-0.62 ± 0.2	-0.8 ± 0.08	-0.72 ± 0.1	-0.56 ± 0.17	-0.49 ± 0.13	-0.87 ± 0.04	-0.68 ± 0.14
Twist_rise (53)	-0.58 ±	-0.53 ±	-0.61 ±	-0.68 ±	-0.61 ±	-0.8 ±	-0.66 ±	-0.82 ±

	0.12	0.23	0.11	0.08	0.11	0.07	0.09	0.04
Twist_roll (43)	-0.67 ± 0.16	-0.57 ± 0.2	-0.8 ± 0.07	-0.73 ± 0.07	-0.46 ± 0.11	-0.81 ± 0.07	-0.86 ± 0.06	-0.9 ± 0.04
Roll (DNA-protein complex) (25)	0.62 ± 0.12	0.54 ± 0.18	0.74 ± 0.08	0.7 ± 0.09	0.52 ± 0.13	0.82 ± 0.07	0.86 ± 0.06	0.85 ± 0.05
Roll (63)	0.73 ± 0.1	0.69 ± 0.13	0.78 ± 0.09	0.75 ± 0.05	0.51 ± 0.1	0.77 ± 0.1	0.9 ± 0.04	0.87 ± 0.04
Roll (90)	0.66 ± 0.14	0.63 ± 0.14	0.79 ± 0.07	0.77 ± 0.06	0.48 ± 0.11	0.74 ± 0.07	0.89 ± 0.05	0.87 ± 0.05
Roll (94)	0.72 ± 0.15	0.67 ± 0.21	0.79 ± 0.06	0.78 ± 0.08	0.5 ± 0.12	0.72 ± 0.1	0.91 ± 0.03	0.87 ± 0.05
Roll (116)	0.56 ± 0.13	0.63 ± 0.13	0.77 ± 0.08	0.71 ± 0.08	0.5 ± 0.12	0.8 ± 0.07	0.89 ± 0.05	0.87 ± 0.04
Roll (119)	0.7 ± 0.13	0.55 ± 0.15	0.75 ± 0.1	0.69 ± 0.09	0.55 ± 0.09	0.82 ± 0.06	0.87 ± 0.04	0.9 ± 0.04
Roll (DNA-protein complex) (84)	0.62 ± 0.16	0.56 ± 0.19	0.71 ± 0.08	0.71 ± 0.1	0.48 ± 0.1	0.78 ± 0.07	0.88 ± 0.06	0.86 ± 0.07
Roll_roll (41)	0.66 ± 0.1	0.63 ± 0.18	0.78 ± 0.07	0.73 ± 0.09	0.54 ± 0.12	0.8 ± 0.06	0.89 ± 0.04	0.88 ± 0.06
Roll_shift (57)	-0.64 ± 0.14	-0.58 ± 0.17	-0.75 ± 0.1	-0.71 ± 0.1	-0.52 ± 0.14	-0.8 ± 0.07	-0.88 ± 0.06	-0.89 ± 0.04
Roll_slide (58)	0.66 ± 0.13	0.6 ± 0.15	0.73 ± 0.09	0.68 ± 0.1	0.52 ± 0.11	0.84 ± 0.03	0.88 ± 0.06	0.91 ± 0.03
Roll_rise (59)	0.66 ± 0.15	0.59 ± 0.19	0.75 ± 0.11	0.71 ± 0.11	0.45 ± 0.11	0.81 ± 0.1	0.9 ± 0.05	0.89 ± 0.03
Tilt_roll (44)	-0.71 ± 0.09	-0.62 ± 0.17	-0.77 ± 0.09	-0.72 ± 0.09	-0.44 ± 0.12	-0.76 ± 0.09	-0.9 ± 0.04	-0.88 ± 0.05
Tilt (DNA-protein complex) (27)	-0.51 ± 0.15	-0.39 ± 0.16	-0.66 ± 0.15	-0.68 ± 0.1	-0.32 ± 0.12	-0.8 ± 0.06	-0.77 ± 0.09	-0.84 ± 0.05
Tilt (62)	0.63 ± 0.15	0.68 ± 0.15	0.75 ± 0.08	0.65 ± 0.16	0.51 ± 0.14	0.76 ± 0.07	0.86 ± 0.05	0.85 ± 0.04
Tilt (89)	-0.65 ± 0.1	-0.63 ± 0.13	-0.74 ± 0.09	-0.72 ± 0.09	-0.53 ± 0.13	-0.77 ± 0.07	-0.88 ± 0.05	-0.88 ± 0.03
Tilt (93)	0.68 ± 0.13	0.64 ± 0.15	0.79 ± 0.06	0.73 ± 0.08	0.53 ± 0.13	0.75 ± 0.07	0.89 ± 0.05	0.89 ± 0.04
Tilt (117)	0.01 ± 0.16	0.02 ± 0.19	-0.07 ± 0.16	0.01 ± 0.13	-0.05 ± 0.14	-0.07 ± 0.18	0.05 ± 0.22	0.02 ± 0.21
Tilt (DNA-protein complex) (83)	0.65 ± 0.13	0.58 ± 0.15	0.73 ± 0.12	0.64 ± 0.12	0.37 ± 0.15	0.79 ± 0.07	0.87 ± 0.04	0.85 ± 0.05
Tilt_tilt (40)	0.71 ± 0.13	0.68 ± 0.16	0.75 ± 0.09	0.74 ± 0.07	0.49 ± 0.14	0.67 ± 0.09	0.88 ± 0.05	0.81 ± 0.07
Tilt_shift (54)	-0.73 ± 0.15	-0.72 ± 0.12	-0.79 ± 0.09	-0.74 ± 0.08	-0.51 ± 0.1	-0.81 ± 0.06	-0.89 ± 0.04	-0.9 ± 0.05
Twist_tilt (42)	-0.62 ± 0.15	-0.62 ± 0.13	-0.73 ± 0.09	-0.69 ± 0.11	-0.53 ± 0.1	-0.79 ± 0.08	-0.89 ± 0.04	-0.87 ± 0.04
Tilt_rise (56)	0.6 ±	0.56 ±	0.69 ±	0.69 ±	0.51 ±	0.82 ±	0.87 ±	0.86 ±

	0.13	0.14	0.11	0.1	0.11	0.08	0.04	0.05
Tilt_slide (55)	0.65 ± 0.13	0.64 ± 0.14	0.8 ± 0.07	0.75 ± 0.06	0.55 ± 0.12	0.79 ± 0.07	0.9 ± 0.05	0.89 ± 0.04
Slide (DNA-protein complex) (28)	0.65 ± 0.12	0.6 ± 0.18	0.75 ± 0.1	0.7 ± 0.07	0.41 ± 0.17	0.71 ± 0.08	0.84 ± 0.06	0.79 ± 0.09
Slide (65)	0.66 ± 0.11	0.6 ± 0.15	0.75 ± 0.1	0.68 ± 0.1	0.49 ± 0.11	0.83 ± 0.06	0.87 ± 0.05	0.86 ± 0.05
Slide (91)	0.69 ± 0.12	0.6 ± 0.16	0.72 ± 0.08	0.67 ± 0.12	0.51 ± 0.11	0.78 ± 0.07	0.88 ± 0.04	0.89 ± 0.03
Slide (96)	0.65 ± 0.15	0.62 ± 0.15	0.75 ± 0.09	0.71 ± 0.09	0.53 ± 0.12	0.82 ± 0.05	0.9 ± 0.04	0.89 ± 0.04
Slide (DNA-protein complex) (86)	0.55 ± 0.12	0.45 ± 0.17	0.61 ± 0.14	0.58 ± 0.08	0.48 ± 0.14	0.8 ± 0.07	0.82 ± 0.07	0.78 ± 0.09
Slide_slide (46)	-0.32 ± 0.13	-0.36 ± 0.15	-0.37 ± 0.18	-0.5 ± 0.18	-0.47 ± 0.14	-0.86 ± 0.04	-0.44 ± 0.19	-0.75 ± 0.07
Slide_rise (50)	-0.62 ± 0.12	-0.56 ± 0.18	-0.65 ± 0.1	-0.6 ± 0.11	-0.57 ± 0.11	-0.46 ± 0.13	-0.79 ± 0.06	-0.6 ± 0.11
Twist_slide (52)	-0.73 ± 0.13	-0.62 ± 0.2	-0.8 ± 0.08	-0.72 ± 0.1	-0.56 ± 0.17	-0.49 ± 0.13	-0.87 ± 0.04	-0.68 ± 0.14
Roll_slide (58)	0.66 ± 0.13	0.6 ± 0.15	0.73 ± 0.09	0.68 ± 0.1	0.52 ± 0.11	0.84 ± 0.03	0.88 ± 0.06	0.91 ± 0.03
Shift_slide (48)	-0.47 ± 0.15	-0.46 ± 0.13	-0.52 ± 0.12	-0.57 ± 0.11	-0.39 ± 0.11	-0.78 ± 0.08	-0.7 ± 0.08	-0.78 ± 0.07
Shift (DNA-protein complex) (30)	-0.69 ± 0.15	-0.58 ± 0.2	-0.76 ± 0.07	-0.73 ± 0.07	-0.54 ± 0.1	-0.84 ± 0.05	-0.89 ± 0.04	-0.91 ± 0.03
Shift (64)	0.56 ± 0.13	0.51 ± 0.14	0.64 ± 0.09	0.51 ± 0.13	0.43 ± 0.17	0.7 ± 0.08	0.8 ± 0.08	0.69 ± 0.09
Shift (95)	-0.64 ± 0.13	-0.67 ± 0.12	-0.76 ± 0.06	-0.72 ± 0.08	-0.61 ± 0.1	-0.72 ± 0.08	-0.84 ± 0.06	-0.82 ± 0.07
Shift (DNA-protein complex) (85)	0.69 ± 0.16	0.58 ± 0.2	0.75 ± 0.08	0.74 ± 0.06	0.48 ± 0.09	0.83 ± 0.05	0.87 ± 0.06	0.91 ± 0.03
Shift_shift (45)	0.67 ± 0.16	0.63 ± 0.16	0.81 ± 0.07	0.8 ± 0.05	0.6 ± 0.07	0.63 ± 0.12	0.9 ± 0.04	0.84 ± 0.06
Twist_shift (51)	-0.55 ± 0.11	-0.53 ± 0.14	-0.7 ± 0.1	-0.64 ± 0.08	-0.51 ± 0.12	-0.8 ± 0.06	-0.86 ± 0.05	-0.84 ± 0.05
Tilt_shift (54)	-0.73 ± 0.15	-0.72 ± 0.12	-0.79 ± 0.09	-0.74 ± 0.08	-0.51 ± 0.1	-0.81 ± 0.06	-0.89 ± 0.04	-0.9 ± 0.05
Roll_shift (57)	-0.64 ± 0.14	-0.58 ± 0.17	-0.75 ± 0.1	-0.71 ± 0.1	-0.52 ± 0.14	-0.8 ± 0.07	-0.88 ± 0.06	-0.89 ± 0.04
Shift_rise (49)	0.68 ± 0.15	0.27 ± 0.15	0.84 ± 0.07	0.29 ± 0.23	0.38 ± 0.12	-0.22 ± 0.17	0.81 ± 0.09	0.04 ± 0.23
Rise (3)	0.63 ± 0.11	0.64 ± 0.13	0.79 ± 0.07	0.76 ± 0.07	0.54 ± 0.12	0.75 ± 0.1	0.9 ± 0.05	0.88 ± 0.05
Rise (DNA-protein complex) (32)	0.71 ± 0.11	0.63 ± 0.17	0.72 ± 0.13	0.72 ± 0.1	0.54 ± 0.11	0.78 ± 0.1	0.88 ± 0.05	0.89 ± 0.04
Rise (66)	0.65 ±	0.63 ±	0.77 ±	0.73 ±	0.48 ±	0.78 ±	0.88 ±	0.88 ±

	0.11	0.17	0.09	0.1	0.12	0.08	0.05	0.05
Rise (97)	0.71 ± 0.1	0.64 ± 0.14	0.76 ± 0.08	0.78 ± 0.06	0.49 ± 0.12	0.84 ± 0.07	0.9 ± 0.04	0.92 ± 0.03
Rise (DNA-protein complex) (87)	0.62 ± 0.15	0.64 ± 0.14	0.75 ± 0.09	0.77 ± 0.06	0.43 ± 0.12	0.77 ± 0.11	0.9 ± 0.06	0.89 ± 0.05
Rise_rise (47)	-0.71 ± 0.13	-0.63 ± 0.17	-0.84 ± 0.06	-0.8 ± 0.07	-0.55 ± 0.1	-0.76 ± 0.09	-0.89 ± 0.05	-0.91 ± 0.04
Slide_rise (50)	-0.62 ± 0.12	-0.56 ± 0.18	-0.65 ± 0.1	-0.6 ± 0.11	-0.57 ± 0.11	-0.46 ± 0.13	-0.79 ± 0.06	-0.6 ± 0.11
Twist_rise (53)	-0.58 ± 0.12	-0.53 ± 0.23	-0.61 ± 0.11	-0.68 ± 0.08	-0.61 ± 0.11	-0.8 ± 0.07	-0.66 ± 0.09	-0.82 ± 0.04
Tilt_rise (56)	0.6 ± 0.13	0.56 ± 0.14	0.69 ± 0.11	0.69 ± 0.1	0.51 ± 0.11	0.82 ± 0.08	0.87 ± 0.04	0.86 ± 0.05
Roll_rise (59)	0.66 ± 0.15	0.59 ± 0.19	0.75 ± 0.11	0.71 ± 0.11	0.45 ± 0.11	0.81 ± 0.1	0.9 ± 0.05	0.89 ± 0.03
Bend (4)	-0.67 ± 0.11	-0.65 ± 0.12	-0.71 ± 0.1	-0.73 ± 0.06	-0.54 ± 0.1	-0.77 ± 0.09	-0.87 ± 0.04	-0.88 ± 0.04
Tip (5)	-0.67 ± 0.11	-0.63 ± 0.14	-0.71 ± 0.1	-0.69 ± 0.09	-0.5 ± 0.14	-0.78 ± 0.08	-0.87 ± 0.05	-0.86 ± 0.05
Inclination (6)	0.0 ± 0.15	-0.04 ± 0.14	0.02 ± 0.11	-0.07 ± 0.13	0.01 ± 0.17	-0.01 ± 0.19	-0.04 ± 0.17	-0.06 ± 0.16
Propeller Twist (20)	0.62 ± 0.15	0.58 ± 0.18	0.73 ± 0.1	0.67 ± 0.07	0.48 ± 0.11	0.85 ± 0.04	0.88 ± 0.05	0.88 ± 0.05
Stacking energy (2)	0.68 ± 0.13	0.58 ± 0.19	0.69 ± 0.13	0.56 ± 0.15	0.45 ± 0.11	0.31 ± 0.19	0.88 ± 0.04	0.55 ± 0.15
Stacking energy (33)	-0.61 ± 0.12	-0.57 ± 0.15	-0.74 ± 0.08	-0.72 ± 0.09	-0.53 ± 0.11	-0.82 ± 0.11	-0.88 ± 0.05	-0.88 ± 0.04
Stacking energy (60)	0.69 ± 0.14	0.64 ± 0.14	0.79 ± 0.1	0.74 ± 0.09	0.53 ± 0.15	0.72 ± 0.06	0.89 ± 0.06	0.87 ± 0.05
Stacking energy (109)	0.66 ± 0.14	0.62 ± 0.13	0.75 ± 0.1	0.69 ± 0.08	0.5 ± 0.13	0.82 ± 0.06	0.88 ± 0.04	0.88 ± 0.04
Melting Temperature (16)	0.66 ± 0.12	0.59 ± 0.16	0.77 ± 0.08	0.7 ± 0.1	0.48 ± 0.15	0.8 ± 0.07	0.89 ± 0.04	0.89 ± 0.05
Melting Temperature (108)	0.67 ± 0.17	0.62 ± 0.16	0.78 ± 0.1	0.72 ± 0.08	0.48 ± 0.14	0.82 ± 0.05	0.9 ± 0.04	0.9 ± 0.03
Enthalpy (22)	-0.65 ± 0.16	-0.66 ± 0.13	-0.74 ± 0.1	-0.75 ± 0.09	-0.49 ± 0.17	-0.77 ± 0.1	-0.9 ± 0.05	-0.91 ± 0.04
Enthalpy (123)	-0.67 ± 0.1	-0.56 ± 0.17	-0.76 ± 0.1	-0.72 ± 0.08	-0.48 ± 0.13	-0.84 ± 0.05	-0.87 ± 0.06	-0.88 ± 0.05
Entropy (23)	-0.69 ± 0.1	-0.65 ± 0.16	-0.82 ± 0.06	-0.77 ± 0.09	-0.51 ± 0.12	-0.82 ± 0.06	-0.89 ± 0.04	-0.9 ± 0.04
Entropy (124)	-0.68 ± 0.15	-0.48 ± 0.15	-0.73 ± 0.13	-0.69 ± 0.11	-0.47 ± 0.11	-0.87 ± 0.04	-0.84 ± 0.06	-0.89 ± 0.04
Free energy (34)	-0.6 ± 0.12	-0.68 ± 0.1	-0.76 ± 0.07	-0.74 ± 0.1	-0.53 ± 0.13	-0.79 ± 0.07	-0.87 ± 0.07	-0.88 ± 0.04
Free energy (35)	-0.67 ±	-0.64 ±	-0.78 ±	-0.73 ±	-0.47 ±	-0.79 ±	-0.9 ±	-0.9 ±

	0.13	0.19	0.07	0.12	0.1	0.06	0.04	0.05
Free energy (36)	-0.64 ± 0.08	-0.61 ± 0.19	-0.74 ± 0.12	-0.69 ± 0.06	-0.51 ± 0.12	-0.83 ± 0.06	-0.89 ± 0.04	-0.89 ± 0.05
Free energy (38)	-0.65 ± 0.12	-0.66 ± 0.17	-0.71 ± 0.12	-0.75 ± 0.07	-0.53 ± 0.11	-0.83 ± 0.06	-0.89 ± 0.04	-0.9 ± 0.04
Free energy (72)	-0.64 ± 0.12	-0.6 ± 0.16	-0.77 ± 0.09	-0.73 ± 0.11	-0.5 ± 0.11	-0.83 ± 0.05	-0.89 ± 0.04	-0.91 ± 0.03
Free energy (73)	-0.61 ± 0.17	-0.58 ± 0.16	-0.78 ± 0.11	-0.76 ± 0.09	-0.49 ± 0.09	-0.81 ± 0.07	-0.89 ± 0.05	-0.9 ± 0.04
Free energy (74)	-0.64 ± 0.1	-0.6 ± 0.17	-0.78 ± 0.09	-0.76 ± 0.07	-0.56 ± 0.14	-0.82 ± 0.07	-0.89 ± 0.05	-0.9 ± 0.03
Free energy (75)	-0.64 ± 0.12	-0.58 ± 0.18	-0.73 ± 0.1	-0.74 ± 0.08	-0.56 ± 0.13	-0.79 ± 0.07	-0.9 ± 0.03	-0.91 ± 0.04
Free energy (125)	-0.66 ± 0.14	-0.6 ± 0.2	-0.74 ± 0.08	-0.75 ± 0.08	-0.5 ± 0.13	-0.83 ± 0.04	-0.88 ± 0.04	-0.89 ± 0.04
Major Groove Width (7)	0.68 ± 0.14	0.62 ± 0.18	0.8 ± 0.07	0.73 ± 0.08	0.49 ± 0.13	0.78 ± 0.07	0.89 ± 0.04	0.88 ± 0.03
Major Groove Depth (8)	-0.64 ± 0.13	-0.69 ± 0.13	-0.77 ± 0.09	-0.72 ± 0.11	-0.51 ± 0.11	-0.76 ± 0.06	-0.86 ± 0.06	-0.87 ± 0.03
Major Groove Size (9)	-0.08 ± 0.21	-0.0 ± 0.16	0.03 ± 0.18	-0.02 ± 0.17	-0.03 ± 0.14	0.01 ± 0.21	-0.07 ± 0.18	0.0 ± 0.22
Major Groove Distance (10)	-0.65 ± 0.14	-0.63 ± 0.14	-0.74 ± 0.07	-0.77 ± 0.07	-0.6 ± 0.11	-0.67 ± 0.08	-0.88 ± 0.04	-0.78 ± 0.07
Mobility to bend towards major groove (18)	-0.65 ± 0.11	-0.6 ± 0.13	-0.75 ± 0.09	-0.71 ± 0.09	-0.53 ± 0.13	-0.81 ± 0.05	-0.88 ± 0.06	-0.9 ± 0.03
Minor Groove Width (11)	-0.64 ± 0.09	-0.65 ± 0.13	-0.77 ± 0.08	-0.72 ± 0.08	-0.46 ± 0.12	-0.78 ± 0.07	-0.87 ± 0.06	-0.87 ± 0.05
Minor Groove Depth (12)	0.59 ± 0.1	0.19 ± 0.2	0.61 ± 0.11	0.08 ± 0.18	0.53 ± 0.16	-0.01 ± 0.12	0.8 ± 0.05	0.13 ± 0.14
Minor Groove Size (13)	0.72 ± 0.12	0.66 ± 0.14	0.78 ± 0.08	0.73 ± 0.07	0.55 ± 0.12	0.8 ± 0.06	0.9 ± 0.04	0.9 ± 0.05
Minor Groove Distance (14)	-0.66 ± 0.14	-0.65 ± 0.16	-0.76 ± 0.08	-0.62 ± 0.13	-0.63 ± 0.11	-0.46 ± 0.14	-0.84 ± 0.05	-0.64 ± 0.11
Mobility to bend towards minor groove (19)	0.65 ± 0.14	0.6 ± 0.15	0.73 ± 0.11	0.71 ± 0.08	0.5 ± 0.14	0.82 ± 0.05	0.87 ± 0.06	0.88 ± 0.05
Probability contacting nucleosome core (17)	-0.67 ± 0.13	-0.58 ± 0.2	-0.81 ± 0.09	-0.69 ± 0.12	-0.32 ± 0.15	-0.8 ± 0.07	-0.9 ± 0.05	-0.9 ± 0.05
Persistance Length (15)	0.69 ± 0.12	0.62 ± 0.17	0.76 ± 0.08	0.76 ± 0.08	0.48 ± 0.13	0.81 ± 0.07	0.88 ± 0.05	0.89 ± 0.04
Slide stiffness (67)	-0.66 ± 0.14	-0.69 ± 0.11	-0.76 ± 0.07	-0.78 ± 0.07	-0.55 ± 0.09	-0.8 ± 0.04	-0.89 ± 0.04	-0.9 ± 0.04
Flexibility_slide (121)	-0.69 ± 0.12	-0.6 ± 0.11	-0.74 ± 0.09	-0.7 ± 0.09	-0.52 ± 0.11	-0.82 ± 0.05	-0.86 ± 0.07	-0.87 ± 0.04
Shift stiffness (68)	0.57 ± 0.11	0.42 ± 0.16	0.65 ± 0.1	0.42 ± 0.17	0.65 ± 0.12	0.28 ± 0.18	0.75 ± 0.09	0.4 ± 0.22
Flexibility_shift (122)	0.7 ±	0.66 ±	0.78 ±	0.74 ±	0.53 ±	0.84 ±	0.87 ±	0.9 ±

	0.1	0.15	0.06	0.05	0.15	0.05	0.05	0.03
Roll stiffness (69)	0.69 ± 0.11	0.58 ± 0.16	0.69 ± 0.11	0.73 ± 0.07	0.52 ± 0.11	0.87 ± 0.05	0.86 ± 0.06	0.88 ± 0.05
Tilt stiffness (70)	0.71 ± 0.11	0.62 ± 0.15	0.77 ± 0.08	0.74 ± 0.1	0.52 ± 0.13	0.73 ± 0.1	0.87 ± 0.05	0.86 ± 0.05
Twist stiffness (71)	-0.62 ± 0.18	-0.49 ± 0.17	-0.75 ± 0.13	-0.64 ± 0.14	-0.24 ± 0.14	-0.76 ± 0.08	-0.78 ± 0.08	-0.82 ± 0.08
Rise stiffness (107)	0.62 ± 0.14	0.59 ± 0.1	0.75 ± 0.07	0.67 ± 0.1	0.49 ± 0.11	0.64 ± 0.12	0.87 ± 0.06	0.82 ± 0.09
